# Macromolecular condensation buffers intracellular water potential

**DOI:** 10.1038/s41586-023-06626-z

**Published:** 2023-10-18

**Authors:** Joseph L. Watson, Estere Seinkmane, Christine T. Styles, Andrei Mihut, Lara K. Krüger, Kerrie E. McNally, Vicente Jose Planelles-Herrero, Michal Dudek, Patrick M. McCall, Silvia Barbiero, Michael Vanden Oever, Sew Yeu Peak-Chew, Benjamin T. Porebski, Aiwei Zeng, Nina M. Rzechorzek, David C. S. Wong, Andrew D. Beale, Alessandra Stangherlin, Margot Riggi, Janet Iwasa, Jörg Morf, Christos Miliotis, Alina Guna, Alison J. Inglis, Jan Brugués, Rebecca M. Voorhees, Joseph E. Chambers, Qing-Jun Meng, John S. O’Neill, Rachel S. Edgar, Emmanuel Derivery

**Affiliations:** 1 MRC Laboratory of Molecular Biology, Cambridge, UK; 2 Department of Infectious Disease, Imperial College London, London, UK; 3 Wellcome Centre for Cell Matrix Research, University of Manchester, Manchester, UK; 4 Cluster of Excellence Physics of Life, TU Dresden, Dresden, Germany; 5 Max Planck Institute of Molecular Cell Biology and Genetics, Dresden, Germany; 6 Max Planck Institute for the Physics of Complex Systems, Dresden, Germany; 7 Department of Biochemistry, University of Utah, Salt Lake City, UT USA; 8 Laboratory of Nuclear Dynamics, Babraham Institute, Cambridge, UK; 9 California Institute of Technology, Pasadena, CA USA; 10 Cambridge Institute for Medical Research, Cambridge, UK; 11 Present Address: Cluster of Excellence Cellular Stress Responses in Aging-associated Diseases (CECAD), Faculty of Medicine and University Hospital Cologne, University of Cologne, Cologne, Germany

**Keywords:** Cell biology, Protein folding, Post-translational modifications, Intrinsically disordered proteins

## Abstract

Optimum protein function and biochemical activity critically depends on water availability because solvent thermodynamics drive protein folding and macromolecular interactions^1^. Reciprocally, macromolecules restrict the movement of ‘structured’ water molecules within their hydration layers, reducing the available ‘free’ bulk solvent and therefore the total thermodynamic potential energy of water, or water potential. Here, within concentrated macromolecular solutions such as the cytosol, we found that modest changes in temperature greatly affect the water potential, and are counteracted by opposing changes in osmotic strength. This duality of temperature and osmotic strength enables simple manipulations of solvent thermodynamics to prevent cell death after extreme cold or heat shock. Physiologically, cells must sustain their activity against fluctuating temperature, pressure and osmotic strength, which impact water availability within seconds. Yet, established mechanisms of water homeostasis act over much slower timescales^2,3^; we therefore postulated the existence of a rapid compensatory response. We find that this function is performed by water potential-driven changes in macromolecular assembly, particularly biomolecular condensation of intrinsically disordered proteins. The formation and dissolution of biomolecular condensates liberates and captures free water, respectively, quickly counteracting thermal or osmotic perturbations of water potential, which is consequently robustly buffered in the cytoplasm. Our results indicate that biomolecular condensation constitutes an intrinsic biophysical feedback response that rapidly compensates for intracellular osmotic and thermal fluctuations. We suggest that preserving water availability within the concentrated cytosol is an overlooked evolutionary driver of protein (dis)order and function.

## Abstract (web summary)

Water thermodynamics drive changes in macromolecular assembly that rapidly restore intracellular water availability in response to physiological fluctuations in temperature, pressure and osmotic strength.

## Main

Water is critical to life, providing a dynamic hydrogen-bonded environment that supports macromolecule solvation. Far from being a passive solvent, water drives protein folding and macromolecular interactions that optimize the network of H_2_O hydrogen bonds^1^. Protein structure, supramolecular assembly and activity are therefore highly sensitive to changes in water thermodynamics^4,5^, which must be tightly regulated to preserve function over multiple timescales. Reciprocally, macromolecules in solution impose a profound energetic cost on neighbouring water molecules within their hydration layers by lowering their translational and rotational entropy^6^. In other words, water is required to hydrate macromolecules and make them fold properly, but this restricts the movement of water molecules and thereby diminishes their availability. Thus, cells must maintain water availability within an optimal range for protein activity, biochemical efficiency and, ultimately, viability.

Water–macromolecule interactions are integral to every biological process. Here we refer to hydration-layer water molecules with lower entropy as structured, in contrast to the free water molecules that form the bulk solvent. The impact of a macromolecule on water depends on both the size and chemistry of its solvent-accessible surface area^6–10^, as the entropic penalty of hydration can be offset by enthalpically favourable interactions between water and hydrophilic surfaces or accentuated at hydrophobic interfaces^5^.

**Fig. 1:**
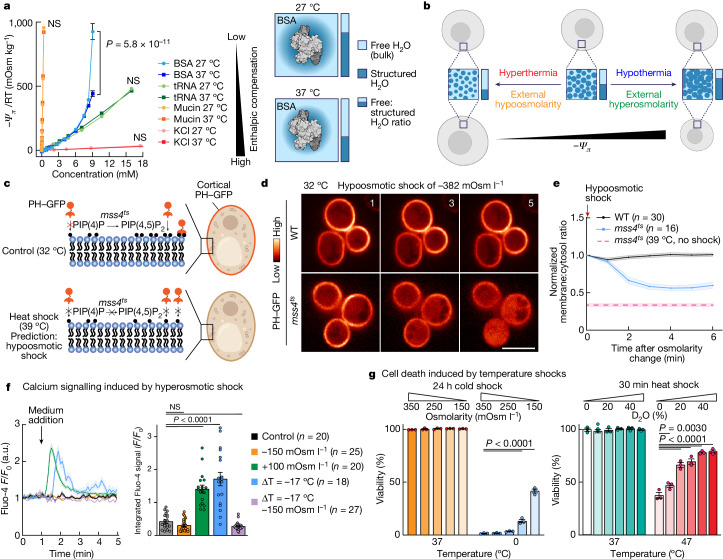
The duality of thermal and osmotic perturbation on water potential and cellular function. **a**, Vapour-pressure osmometry measurements for the indicated solute concentrations and temperatures (left). Data are mean ± s.e.m. *n* = 3. BSA exerts a nonlinear effect on solvent thermodynamics that is accentuated at 27 °C compared with at 37 °C, whereas other macromolecules exhibit temperature-independent quasi-linear relationships, indicating that this is not a crowding effect (Extended Data Fig. 1). Right, instead, we propose that structured water increases as the temperature decreases. Statistical analysis was performed using two-way analysis of variance (ANOVA). **b**, The model for duality of osmotic and thermal perturbations on free:structured water in cells (Supplementary Video 3). **c**–**e**, Hypoosmotic shock phenocopies heat shock for thermosensitive yeast mutants. **c**, Using WT *mss4* and thermosensitive *mss4*^*ts*^ strains of *S. cerevisiae* expressing PIP(4,5)P_2_ GFP probe (PH–GFP) to monitor *mss4* PIP(4) kinase activity, we found that the cortical GFP signal decreased when cells shifted from permissive (32 °C) to restrictive (39 °C) temperatures (Extended Data Fig. 2). We predicted that hypoosmotic shock at 32 °C would mimic 39 °C heat shock. **d**, The PH–GFP signal after hypoosmotic shock was monitored using spinning-disk confocal microscopy (SDCM; single confocal planes). Scale bar, 5 µm. **e**, The normalized cortical to cytosol ratio of the PH–GFP signal. Data are mean ± s.e.m. As predicted, thermosensitive *mss4*^*ts*^ mutants lose the cortical PH–GFP signal after hypoosmotic shock but the WT strains do not, excluding indirect effects on PH–GFP signal or PIP_2_ levels through membrane tension or PIP(4,5)P_2_ phosphatases. **f**, The interaction between osmolarity and temperature on calcium signalling in primary chondrocytes (Fluo-4 *F*/*F*_0_ signal). Data are mean ± s.e.m. *n* values indicate the number of fields of view analysed. See also Extended Data Fig. 3. Statistical analysis was performed using one-way ANOVA followed by Dunnett’s test; *P* values are indicated. **g**, Manipulating water thermodynamics rescued Raji cell viability after cold or heat shock. Data are mean ± s.e.m. *n* = 3. Hypoosmotic conditions increased survival at 0 °C. Similarly, D_2_O increased survival after extreme heat shock as increased H-bonding network strength preserves the hydration layer size. Statistical analysis was performed using two-way ANOVA with Dunnett’s post hoc test; *P* values are indicated. Source data

**Extended Data Fig. 1:**
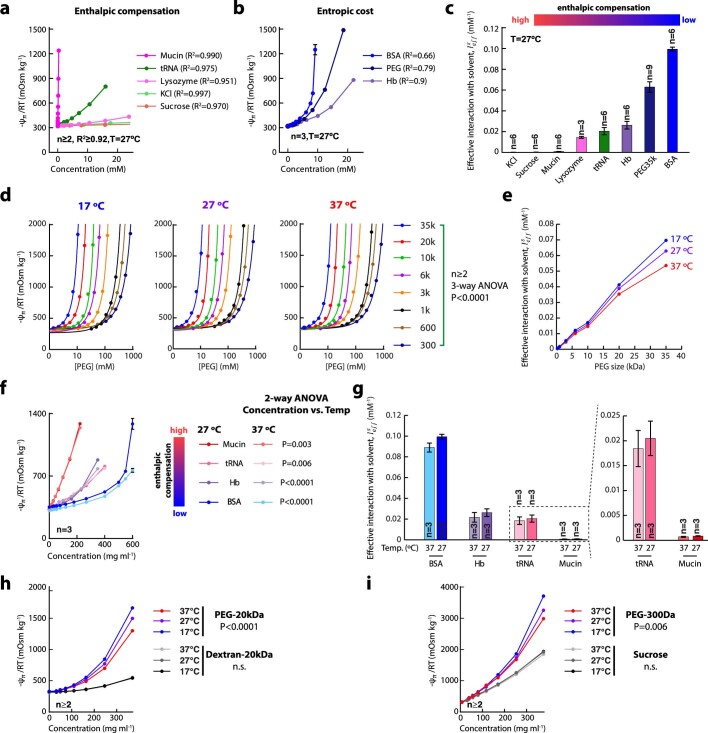
Osmotic potential of simple and macromolecular solutes as a function of concentration and temperature. (**a**-**b**) The osmotic potential (mean ± SEM) of indicated macromolecules at various concentrations was determined at 27 °C by the vapour pressure method, data corresponds to Fig. 1a. Charged or hydrophilic small solutes, as well as macromolecules with predominantly hydrophilic surfaces, partake in enthalpically-compensated interactions with the solvent such that osmotic potential increases linearly with concentration. Conversely, the osmotic potential of macromolecules (BSA, PEG, Hb) with less enthalpically-favourable solvent interactions show a marked departure from linearity as concentration increases. Goodness-of-fit for a centred first order polynomial is shown. (**c**) A single parameter describing each solute’s effective departure from linearity (that is, its effective interaction with the solvent ${I}_{{\rm{eff}}}^{s}$, displayed with 95% confidence interval as error bars) can be derived by fitting the data to Equation 1, as proposed by Fullerton and colleagues^11,13^; see methods for details. The higher the ${I}_{{\rm{eff}}}^{s}$, the more uncompensated or energetically unfavourable “structured” water is generated per unit mass of solute, and the less linear the osmometry curve is with respect to concentration. n: number of osmometry curves fitted simultaneously to evaluate ${I}_{{\rm{eff}}}^{s}$ in each condition (see supplementary discussion). (**d**) PEG models the effect of less enthalpically-favoured biological macromolecules with high ${I}_{{\rm{eff}}}^{s}$ on solvent thermodynamics, since it has no solubility limit and does not partition from the solvent. The osmotic potential of PEG changes as a function of concentration, molecular weight, and temperature, with a highly significant three-way interaction between the three variables. (**e**) ${I}_{{\rm{eff}}}^{s}$ values from the data presented in (**d**) illustrates how solvent thermodynamics become increasingly sensitive to temperature drop as ${I}_{{\rm{eff}}}^{s}$ increases (that is for larger PEG size). (**f**) As predicted by PEG, the temperature sensitivity of a less enthalpically-favoured macromolecule’s effect on solvent thermodynamics is greater for macromolecules with higher ${I}_{{\rm{eff}}}^{s}$. For example, for solutions of mucin and BSA with osmotic potential >1000 mOsm kg^−1^ at 27 °C degrees, a 10 °C increase in temperature halves the osmotic potential of the BSA solution but has minimal effect on the osmotic potential of mucin solution. Statistics indicated. (**g**) ${I}_{{\rm{eff}}}^{s}$ values (±95% confidence interval) from the data presented in (**f**) illustrates how the temperature- sensitivity of a less enthalpically-favoured macromolecule’s effect on solvent thermodynamics is greater for those with higher ${I}_{{\rm{eff}}}^{s}$. n: number of osmometry curves measured at each temperature to evaluate ${I}_{{\rm{eff}}}^{s}$. (**h**,**i**) The concentration and temperature-dependent effect of macromolecules such as PEG on solvent thermodynamics is not attributable to macromolecular crowding or excluded volume effects, since identical concentrations of more enthalpically favourable but similarly-sized carbohydrates (**h**: PEG-20kDa versus dextran-20kDa; **i**: PEG-300Da versus Sucrose 349 Da) have more modest effects on osmotic potential that are not sensitive to temperature over this range. Two-way ANOVA for temperature versus concentration statistics are indicated. Throughout n: number of independent repeats. Please note that absolute osmotic potential is shown here, which includes 320 mOsm kg^−1^ due to the buffer used throughout (20 mM Tris-HCl pH 7.4/150 mM KCl). Whereas, for clarity, baseline-subtracted vapour pressure measurements are presented in main figures. Source data

The total thermodynamic potential energy of water, or water potential (*Ψ*), has pressure, gravimetric and other components, but the most biologically relevant is the solute or osmotic potential (*Ψ*_*π*_ and −*Ψ*_*π*_, respectively), defined as zero for pure water. The addition of solutes lowers the potential energy by reducing the free water available in the system to perform work. In an ideal solution, *Ψ*_*π*_ depends solely on the number of particles in solution, rather than their nature, and can be measured directly from colligative properties such as vapour pressure. However, the behaviour of water in the concentrated intracellular environment is far from ideal. In vitro, hydrophilic macromolecules such as glycosylated mucin or tRNA have a much greater impact on water potential compared with smaller solutes such as KCl or sucrose (Fig. 1a and Extended Data Fig. 1a), as their hydration constrains many more water molecules. For these highly polar or charged molecules, *Ψ*_*π*_ changes linearly with concentration, *C*, as described by van’t Hoff’s law −*Ψ*_*π*_ = *iCRT*, where *i* is the van’t Hoff factor, *R* is the gas constant and *T* is the temperature in kelvin. As expected, a small decrease in temperature from 37 °C (310 K) to 27 °C (300 K) has a minimal impact on the water potential of these solutions.

By contrast, *Ψ*_*π*_ deviates significantly from van’t Hoff’s linearity in concentrated solutions of macromolecules with exposed surfaces that are more hydrophobic and less electrostatically favourable, such as bovine serum albumin (BSA), haemoglobin (Hb) or polyethylene glycol (PEG)^11–13^ (Fig. 1a and Extended Data Fig. 1b). Departure from ideality reflects how much hydrogen bonding (enthalpy) and water movement (entropy) is perturbed compared with pure water. Owing to its size, mucin restricts the movement of thousands of water molecules, but its heavily glycosylated surface provides sufficient enthalpic compensation (Fig. 1a). However, most macromolecular surfaces have both hydrophilic and hydrophobic regions that differentially alter water motion and hydrogen bonding networks compared with bulk solvent. To formally evaluate this effective solute–water interaction, denoted ${I}_{{\rm{eff}}}^{s}$, we followed the work of Fullerton and colleagues and modelled our osmometry curves with the empirical equation (1)^11,13^ (the rationale is provided in the Supplementary Discussion):

1 $${-\varPsi }_{\pi }=\frac{AC}{1-{I}_{{\rm{e}}{\rm{f}}{\rm{f}}}^{s}C},$$

where *C* is the solute concentration and constant *A* is a function of solute mass. A smaller ${I}_{{\rm{eff}}}^{s}C$ component indicates less deviation from van’t Hoff’s linearity (that is, $\mathop{{\rm{lim}}}\limits_{{I}_{{\rm{eff}}}^{s}C\ll 1}A=iRT$), and fitting our data to equation (1) confirmed that proteins such as BSA have very high ${I}_{{\rm{eff}}}^{s}$ values compared with more hydrophilic macromolecules and small solutes (Extended Data Fig. 1c). Furthermore, as expected, ${I}_{{\rm{eff}}}^{s}$ scales with chain size for polymers such as PEG (Extended Data Fig. 1d,e). Notably, the water potential of concentrated protein solutions becomes sensitive to physiologically relevant temperature changes. For example, a modest temperature decrease from 37 °C (310 K) to 27 °C (300 K) alters the *Ψ*_*π*_ of a BSA solution by twofold as bulk solvent becomes limiting (Fig. 1a). Overall, the departure from van’t Hoff’s linearity (${I}_{{\rm{eff}}}^{s}$) of BSA, Hb and PEG was strongly increased as temperature decreased (Extended Data Fig. 1d–i).

Molecular dynamics simulations and studies of protein cold denaturation have both previously suggested that increased macromolecular hydration occurs at lower temperatures and is also consistent with temperature-dependent changes in linear alcohol hydration^14–16^. The notable effect of temperature decrease on *Ψ*_*π*_ was observed only in concentrated colloidal solutions, where the relationship with macromolecule concentration departs from linearity. We infer that this occurs because more water molecules are recruited to hydration layers as temperature falls, as the increased strength of hydrogen bonding extends the structured water surrounding each macromolecule. Similar to higher macromolecule concentrations in which there are more surfaces to hydrate, colder temperatures would increase the proportion of structured water compared with free water to greatly increase −*Ψ*_*π*_. In both cases, the entropic cost of structured water increases disproportionately as the bulk solvent becomes limiting (Fig. 1a).

The cellular interior is a concentrated colloidal solution. From our observations in solution, we predicted that intracellular water potential would be similarly sensitive to acute changes in macromolecular hydration elicited by perturbation of temperature and extracellular osmotic strength, because both affect the ratio of free to structured water (Fig. 1b). For example, an abrupt fall in temperature is expected to decrease the proportion of available free water to structured water, similar to external hyperosmotic conditions in which free bulk water immediately leaves the cell to restore the *Ψ*_*π*_ equilibrium across the cell membrane.

## Duality of thermal and osmotic shocks

**Extended Data Fig. 2:**
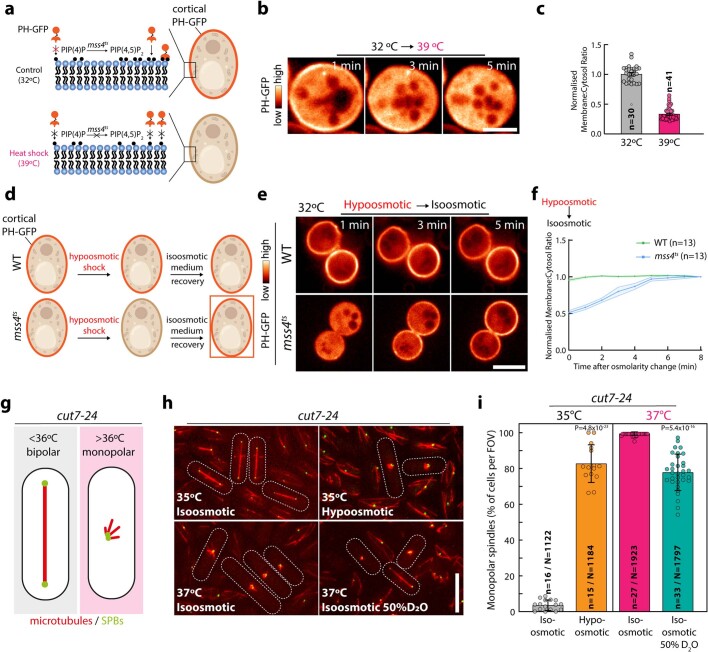
Heat shock and hyperosmotic shocks have similar phenotypes in yeast thermosensitive mutants. During heat denaturation, protein stability (and so activity) decreases as the conformational entropy gain upon unfolding prevails over the entropy loss due to hydrophobic hydration^5^. Thermosensitive mutant proteins simply have lower melting temperatures than their wild type counterparts^92,93^, i.e., they have lower intrinsic stability. In this study we highlight that, conceptually, from the perspective of water, a hypoosmotic treatment has similar thermodynamic consequences to increased temperature. Indeed, increasing temperature increases the ratio of free:structured water because the relative radius of hydration shells decreases and liberates water into the bulk solvent, which is equivalent to what happens under hypoosmotic conditions when water influx means that more bulk water is available compared with water in protein hydration shells. Thus, it is expected that a hypoosmotic treatment recapitulates the consequences of increased temperature for thermosensitive mutants: both treatments increase water availability and so reduce the relative cost of hydrophobic hydration for the solvent as a whole, leading to unfolding of a protein that is already on the threshold of denaturation. (**a**-**f**) Similarity of heat shock and hyperosmotic shock treatment for the *S.cerevisiae* mutant *mss4*^*ts*^. (**a**-**c**) Validation of the *mss4*^*ts*^ mutant used in this study. (**a**) Principle of the experiment: *S. cerevisiae* cells expressing a PIP(4,5)P_2_ GFP probe (PH-GFP) in a thermosensitive mutant background for the PIP(4) kinase *mss4* are rapidly shifted from the permissible temperature (32 °C) to the restrictive temperature (39 °C). Inactivation of *mss4* leads to loss of cortical PIP(4,5)P_2_ and thus of cortical GFP signal. (**b**) PH-GFP signal over time (SDCM) upon rapid heat shock as depicted in (**a**). Elapsed time after temperature change in min. (**c**) Quantification of the effects seen in (**b**), see methods. Heat shock induces a quantitative loss of PH-GFP signal from the plasma membrane. (**d**-**f**) Hypoosmotic shock phenocopies heat shock for *mss4*^*ts*^. (**d**) Principle of the experiment: *S. cerevisiae* cells expressing PH-GFP in a *wildtype* or *mss4*^*ts*^ mutant background were subjected to a hypoosmotic shock (405 mOsm l^−1^ to 23 mOsm l^−1^), then shifted back to isoosmotic medium and the cortical signal of PH-GFP was monitored by SDCM during the latter phase. This is the recovery time-course of the experiment presented in Fig. 1d–f. Experiment was performed at the permissive temperature (32 °C). (**e**) PH-GFP signal over time during recovery into isoosmotic medium as described in (**a**). Elapsed time after medium change indicated in min. (**f**) Quantification of the effects seen in (**e**). Note that on the contrary to WT, in the *mss4*^*ts*^ background, the PH-GFP signal is absent from the membrane in hypoosmotic medium, mimicking the heat shock treatment, but that this is alleviated upon shifting the cells into isoosmotic medium. (**g**-**i**) Similarity of heat shock and hyperosmotic shock treatment for the *S. pombe* mutant *cut7-24*. (**g**) Principle of the experiment: *S. pombe* cells expressing a spindle pole body probe (Sid4-GFP) and a microtubule probe (mCherry-Atb2, yeast homologue of α-tubulin) in a thermosensitive mutant background for the mitotic kinesin Cut7, *cut7-24*. Cut7 is a kinesin-5 motor, which is essential for the formation of a bipolar mitotic spindle by promoting the separation of the two spindle poles (yeast equivalent of centrosomes), and thus crucial for cell division^67,94^. Consequently, temperature-induced degradation of Cut7 by shifting thermosensitive *cut7-24* from the permissive (<36 °C) to the restrictive (>36 °C) temperature leads to the formation of monopolar spindles in almost all cells, while at the permissive temperature most *cut7-24* mutant cells form bipolar spindles and proceed through cell division^18,67^. (**h**) Representative field of views of *cut7-24* cells expressing mCherry-Atb2 and Sid4-GFP in indicated conditions imagined by SDCM. (**i**) Quantification of the effect seen in (h) by manually scoring cells showing monopolar spindles in movies (see methods; Mean ± SD; n: number of fields of view (FOVs) quantified in 2–4 independent experiments pooled. Statistics unpaired t-test with respect to respective isoosmotic control. ****: P < 0.0001; N: total number of cells scored in each condition without averaging per FOV). Upon temperature shift to 37 °C almost 100% of mitotic *cut7-24* cells form monopolar spindles that never reach bipolarity (mean ± SD 99 ± 1%, n = 17 FOVs), compared with 35 °C. As with *S. cerevisae mss4*^*ts*^, exposing the *cut7-24* cells at the permissive temperature to hypoosmotic condition (5% vol:vol of YES medium in water) mimicked the phenotype observed at the restrictive temperature and resulted in a dramatic increase of monopolar spindles. Moreover, the effects of the restrictive temperature were partially rescued by growing the cells overnight in medium supplemented with 50% D_2_O. Note that hypoosmotic shock did not elicit any monopolar spindle in WT cells (of 422 cells, 0 cells formed monopolar spindles; data not shown). All panels in (b, e) correspond to single confocal planes while panels in (h) corresponds to maximum intensity projections (7 planes, Δz = 1 µm). Scale bars: 3 µm (b), 5 µm (e) and 10 µm (h). Source data

In light of our findings, we hypothesized that acute changes in temperature could rapidly affect macromolecular structure and enzymatic activity indirectly by altering water availability and thermodynamics, in addition to direct kinetic effects. If true, decreased external osmotic strength would have an equivalent effect on intracellular *Ψ*_*π*_ to increased temperature (Fig. 1b). Initially, we tested this prediction using temperature-sensitive yeast mutants. Thermosensitive mutations are thought to modify protein stability so that a slight elevation in temperature causes the protein to reversibly unfold^17^. After transfer to the restrictive temperature of 39 °C, the well-established thermosensitive mutant of the phosphatidylinositol 4,5-bisphosphate (PIP(4,5)P_2_) kinase Mms4p in *Saccharomyces cerevisiae*, *mms4*^*ts*^, becomes inactive and PIP(4,5)P_2_ is therefore lost from the plasma membrane^17^ (Fig. 1c and Extended Data Fig. 2a–c). Notably, an external hypoosmotic shock mimicked the temperature phenotype and led to a similar loss in PIP(4,5)P_2_ signal at the membrane in the *mms4*^*ts*^ mutant, but not in the wild-type (WT) controls, over comparable timescales (Fig. 1d,e and Extended Data Fig. 2d–f for recovery control). We validated this concept in another thermosensitive mutant in another species. The established *Schizosaccharomyces pombe* thermosensitive mutant of the spindle assembly kinesin-5 Cut7, *Cut7-24*, induces monopolar spindle formation at the restrictive temperature^18^. As we predicted, this phenotype was also observed at the permissive temperature after external hypoosmotic shock (Extended Data Fig. 2g–i).

**Extended Data Fig. 3:**
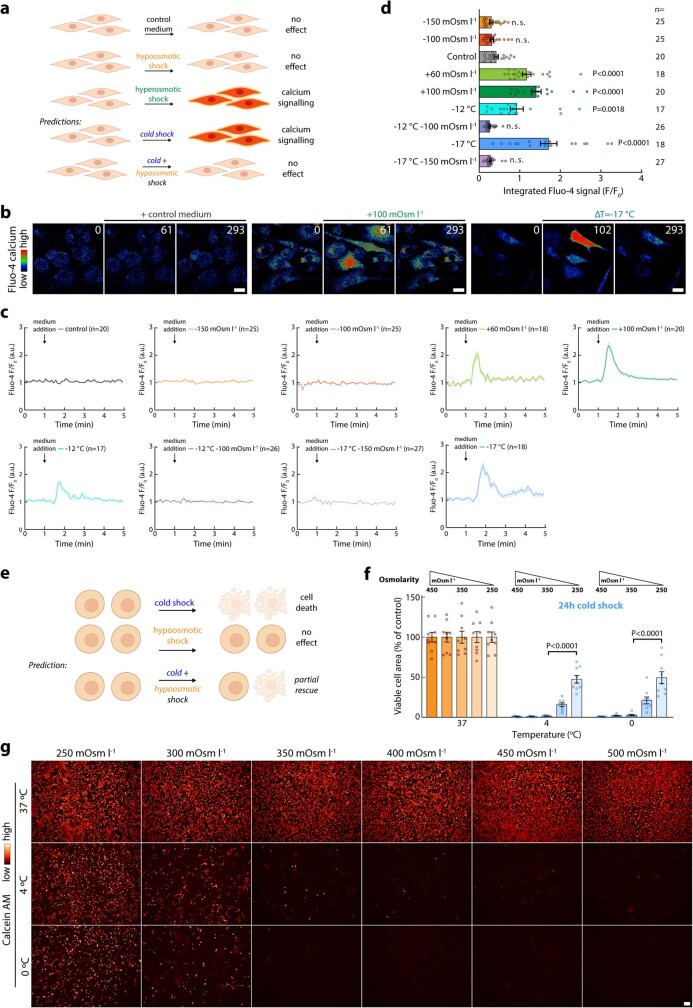
Compensation of osmotic shock by temperature shifts in calcium signalling and hypoosmotic treatment attenuates cell death from prolonged cold. (**a**) Rationale of the experiment. On the contrary to hypoosmotic shocks, hyperosmotic shocks trigger calcium signalling in primary chondrocytes. Our model thus predicts that a cold shock should trigger calcium signalling, and that a hypoosmotic shock should compensate for this. (**b**) Calcium dynamics were analysed live in primary chondrocytes using the Fluo-4 dye and confocal microscopy. Osmotic and/or temperature challenge was applied 1 min after the start of the acquisition. (**c**) Fluo-4 F/F_0_ signal curves for indicated conditions (mean ± SEM. n = number of fields of view analysed). Note that some curves also presented in Fig. 1f, are reproduced here for clarity. (**d**) Peak change of the Fluo-4 F/F_0_ signal in the conditions presented in (**c**) (mean + SEM). Statistics one-way ANOVA followed by Dunnett’s post-hoc test (P value indicated; n = number of fields of view analysed). (**e**) Rationale of the experiment. Cells were exposed to osmotic or temperature shocks, or a combination of both, then the viability was measured. The model predicts that hypoosmotic shock should rescue cold-induced cell death. (**f-g**) Representative images and quantification of human foreskin fibroblasts, treated or not with a 24 h cold shock in media of the indicated osmolarity. Cells were stained with Calcein AM dye to assess viability. (**f**) Cell viability quantification in samples presented in (**g**). Mean ± SEM; n = 9 fields of view analysed per condition; Statistics: two-way ANOVA followed by Dunnett’s post-hoc test (P value indicated). Panel representative of N = 2 repeats. Scale bars: 20 µm (**b**), 200 µm (**g**). Source data

We next investigated the duality of thermal and osmotic perturbation in primary mouse chondrocytes, in which Ca^2+^ signalling in response to changes in osmotic strength is well established in vivo^19^. Using Fluo-4 imaging, we confirmed that acute hyperosmotic treatment evoked a dose-dependent increase in Ca^2+^ signalling and validated our prediction that an acute temperature decrease would evoke a similar response, whereas hypoosmotic treatment had no effect (Fig. 1f and Extended Data Fig. 3a–d). Critically, when hypoosmotic treatment and temperature decrease were applied simultaneously, the Ca^2+^-signalling response was completely abolished (Fig. 1f). This suggests that the observed Ca^2+^ signalling in chondrocytes is regulated by the ratio of intracellular free:structured water. Given the fast, second-scale kinetics of this response, we propose that membrane Ca^2+^-channel opening may directly respond to *Ψ*_*π*_, as opposed to indirect modulation by sensors of solute concentration or membrane tension.

Finally, we tested this duality hypothesis at the global level of the cell, focusing on the viability of mammalian cells during stress. Cellular responses to osmotic and thermal stress are thought to involve different pathways and mechanisms^20,21^, but our previous observations suggested a shared component that senses and responds to resultant changes in water availability. We reasoned that overwhelming the cell’s ability to buffer intracellular *Ψ*_*π*_ could contribute to cell death when exposed to temperature extremes. Consequently, we predicted that manipulation of solvent thermodynamics to oppose temperature-driven changes in the ratio of free:structured water would attenuate the effect of heat or cold shock. Consistent with this prediction, by combining hypoosmotic shock with prolonged exposure to 0 °C, we observed that viability was markedly increased for both suspension and adherent cells (Fig. 1g (left) and Extended Data Fig. 3e–g). According to our model, the deleterious increase in the proportional amount of structured water at low temperatures was counteracted by increased uptake and availability of bulk free water under hypoosmotic conditions (Fig. 1b).

**Extended Data Fig. 4:**
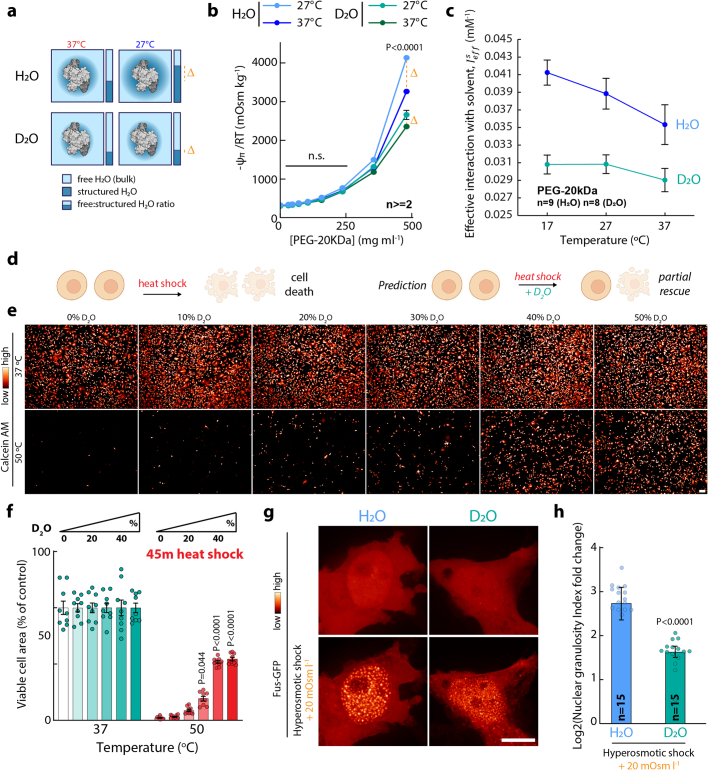
Co-treatment with D_2_O attenuates the effects of acute temperature and external osmolarity change, and attenuates cell death from heat shock. (**a**-**c**) D_2_O attenuates variation in osmotic potential as a function of PEG concentration and temperature. (**a**) since D_2_O forms stronger H-bonds compared to H_2_O, proteins should have a reduced impact on the structured/free water ratio in the presence of D_2_O, and substitution of H_2_O with D_2_O should counter the effects of lowering temperature on the thickening of hydration shells by increasing the enthalpic favourability of hydration shells that incorporate heavy water. (**b**) Osmotic potential measured by vapour pressure for solutions of PEG-20kDa at indicated concentration in buffer containing 100% water (noted H_2_O) or 50% heavy water/50% water (noted D_2_O) solvent at indicated temperature. Note that the H_2_O curves are identical to those presented in Extended Data Fig. 1, shown here for reference. Statistics: three-way ANOVA considering the solvent (D_2_O vs. H_2_O), PEG concentration and temperature as variables followed by a Tukey post-hoc test (significance indicated; ****: P < 0.0001, mean ± SEM). Note that the D_2_O attenuates the effect of PEG concentration on osmotic potential, and that the effect of temperature is lower in D_2_O versus H_2_O. Both observations are consistent with increased enthalpic favourability of macromolecular-solvent interactions when H_2_O is exchanged for D_2_O. n = 3 independent osmometry curves measured for all conditions except D_2_O/37 °C where n = 2; (**c**) Reduced sensitivity of ${I}_{{\rm{eff}}}^{s}$ to temperature for PEG-20kDa in D_2_O (${I}_{{\rm{eff}}}^{s}$ ± 95% confidence intervals are plotted). n: number of independent osmometry curves fitted simultaneously to evaluate ${I}_{{\rm{eff}}}^{s}$ in each condition. (**d**) The main consequence of a 47 °C heat shock on mammalian cells is the thermal denaturation of many different proteins. Thermally denatured proteins aggregate because they overload the cellular capacity to refold and degrade them; where aggregation is a second order process dependent on the concentration of denatured and partially unfolded proteins, and the failure to resolve this results in cell death^22,95^. Protein unfolding occurs for two reasons: (1) macromolecules acquire sufficient kinetic energy to overcome the energy barrier for the entropic cost of hydrophobic hydration, and (2) because the relative cost of hydrophobic hydration falls as temperatures increase because the average number and strength of hydration bonds in bulk solvent is temperature dependent^5^. Both of these kinetic factors can therefore be understood in terms of solvent thermodynamics and so reducing water availability through increased solute concentration would be expected to increase protein thermal stability. However, for cells, such supraphysiological hyperosmotic treatment has two major consequences: the loss of cellular water^29^, and an increased concentration of cellular macromolecules^50^. Whilst the first should disfavour protein unfolding by lowering intracellular Ψ_π_, the second will drastically favour the aggregation of proteins that have unfolded and so render cells at least as liable to cell death, if not more so. Similar to high sucrose concentrations, a stabilizing effect of D_2_O on protein structure in solution is well established in the biochemistry field^24^. The classical way to explain this effect is that D_2_O forms stronger hydrogen bonds than H_2_O (heavy ice melts at 3.8 °C). We therefore employed D_2_O to demonstrate the solvent-dependence of cell death upon heat shock because D_2_O immediately equilibrates over the cell membrane and so cannot affect cell volume. (**e**) Representative fields of view of adherent human foreskin fibroblasts cells, treated or not with a 45 min 50 °C heat shock in media containing the indicated percentages of D_2_O (v/v), and stained with Calcein AM dye to assess viability. (**f**) Cell viability quantification in samples presented in (**e**). Mean ± SEM; n = 9 fields of view analysed per condition. Panel representative of N = 2 repeats. Statistics: two-way ANOVA followed by Dunnett’s post-hoc test (P value indicated). (**g**) D_2_O attenuates protein condensation induced by acute hyperosmotic treatment. U2OS cells transiently expressing FusLC-GFP were equilibrated in media containing 0% or 50% D_2_O for two min, then subjected to a 20 mOsm l^−1^ hyperosmotic treatment in media containing 0% or 50% D_2_O, respectively. The degree of FusLC-GFP was assessed by live SDCM before and after the treatment. (**h**) Log_2_ of the fold change in granulosity index for nuclear FusLC-GFP upon osmotic challenge in the presence or absence of D_2_O (median ± 95% confidence interval). Statistics: Mann-Whitney test. n: number of cells analysed. Note that condensation upon hyperosmotic shock is partially alleviated in the presence of D_2_O. Scale bars: 10 µm (**e**), 200 µm (**g**).

At supraphysiological temperatures (>43 °C), protein denaturation arises through a combination of increased kinetic energy and decreased effective strength of hydrogen bonds. The resulting aggregation of unfolded proteins leads to cell death^22^. In dilute solutions, protein thermal stability can be rescued by *Ψ*_*π*_ manipulations, such as greatly increasing osmolarity with small solutes (for example, sucrose^23^) or by using heavy water (D_2_O) instead of H_2_O (ref. ^24^). In cells, the high extracellular osmolarities that would be needed to increase protein stability also lead to decreased cell volume that increases the aggregation of thermally denatured proteins and cell death. By contrast, replacing hydrogen with deuterium has complex effects but, importantly, cell volume is unchanged and hydrogen bonds are stronger in D_2_O compared with in H_2_O (refs. ^25–27^). Consequently, the water potential of PEG/D_2_O solutions are less sensitive to changes in macromolecule concentration and temperature compared with H_2_O solutions (Extended Data Fig. 4a,b), that is, the effective interaction between solute and D_2_O (${I}_{{\rm{eff}}}^{s}$) is lower than for H_2_O and the temperature dependency of ${I}_{{\rm{eff}}}^{s}$ is attenuated for D_2_O (Extended Data Fig. 4c). We therefore predicted that substitution of H_2_O with D_2_O in macromolecule hydration layers would mitigate the effect of high temperature and preserve protein stability. In agreement with this conceptual framework, we found that D_2_O substantially rescued cell viability from an otherwise cytotoxic heat shock (Fig. 1g (right) and Extended Data Fig. 4d–f). Similarly, D_2_O partially rescued the effects of the restrictive temperature on monopolar spindle formation in the *S. pombe* thermosensitive mutant *Cut7-24* (Extended Data Fig. 2h,i).

We conclude that, while temperature and osmotic shock clearly have many different effects on cell biology, their intersection with regards to solvent thermodynamics (*Ψ*_*π*_) impacts fundamental properties of life such as protein structure and function and, ultimately, cell survival.

## *Ψ*_*π*_ homeostasis involves condensates

Our results highlight the need for cells to maintain *Ψ*_*π*_ homeostasis over different challenges and timescales. In the body, cells must tolerate and adapt to anatomical and temporal variation in temperature and osmotic strength. The osmolality of plasma is around 290 mOsm l^−1^ compared with 1,200 mOsm l^−1^ in parts of the human kidney, for example, while the temperature of the dermal tissue is around 30 °C whereas that of the deep brain can exceed 40 °C in healthy individuals^28^. Yet, cell volume is robust against physiological fluctuations in osmolarity, temperature, pressure and intracellular macromolecule concentration^29^, the effect of which on intracellular water potential is almost instantaneous. This suggests that intracellular *Ψ*_*π*_ is defended over subsecond timeframes to maintain the optimum balance of free:structured water for protein function and biochemical activity.

**Fig. 2:**
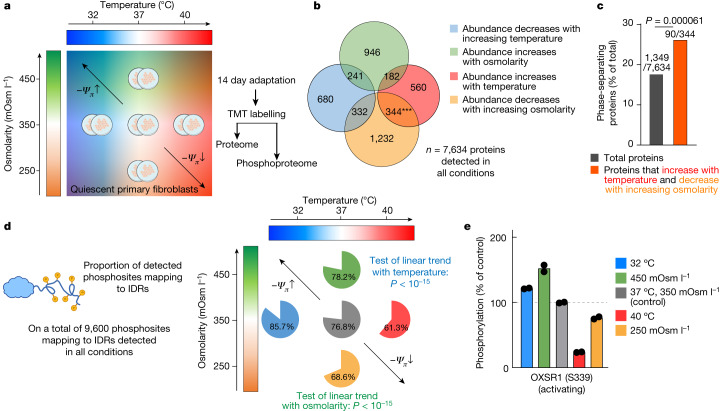
Long-term thermal and osmotic adaptation of the proteome and phosphoproteome. **a**, The (phospho)proteomics experimental design. Quiescent primary fibroblasts were cultured in duplicate for 14 days under the indicated conditions, for adaptation to increased or decreased temperature/osmotic strength. Quantitative proteomics (tandem mass tag (TMT)-MS/MS) was then performed to analyse the proteome and phosphoproteome differences between samples. **b**, The number of proteins of which the abundance changed significantly in a particular direction, and the overlap between conditions. Green, proteins of which the abundance significantly increased with increasing osmolarity (directly correlated with external osmolarity); orange, proteins of which the abundance significantly decreased with increasing osmolarity (inversely correlated with external osmolarity); red, proteins of which the abundance increased with increasing temperature (directly correlated with temperature); blue, proteins of which the abundance decreased with increasing temperature (inversely correlated with temperature). Statistical analysis was performed using Fisher’s exact tests comparing the overlap between different sets of proteins, given the total number of proteins detected. **c**, The percentage of proteins reported as phase separating in the PhaSepDB high-throughput database v.1. Statistical analysis was performed using a one-proportion *z*-test. **d**, The proportion of phosphosites predicted to map to IDRs, comparing subsets of phosphosites that change significantly in a particular direction against the overall percentage of IDR phosphorylation (76.8%). Phosphopeptides that increase with temperature and decrease with osmolarity have a significantly lower proportion of IDR phosphorylation, whereas phosphopeptides that increase with temperature and decrease with osmolarity have significantly higher proportion of IDR phosphorylation (proportion *z*-test, Benjamini–Hochberg-adjusted *P* < 1 × 10^−15^ for both temperature and osmolarity). Predicted disorder information was available for 12,495 out of 14,530 detected phosphopeptides. **e**, Representative example of an IDR phosphosite, at which the phosphorylation level changed in a manner consistent with *Ψ*_*π*_ homeostasis. *n* = 2. OXSR1 kinase is a key effector of osmotic balance, activated by Ser339 phosphorylation. The effect of hyperosmotic challenge on OXSR1 phosphorylation is fully consistent with recent results^50^.

**Extended Data Fig. 5:**
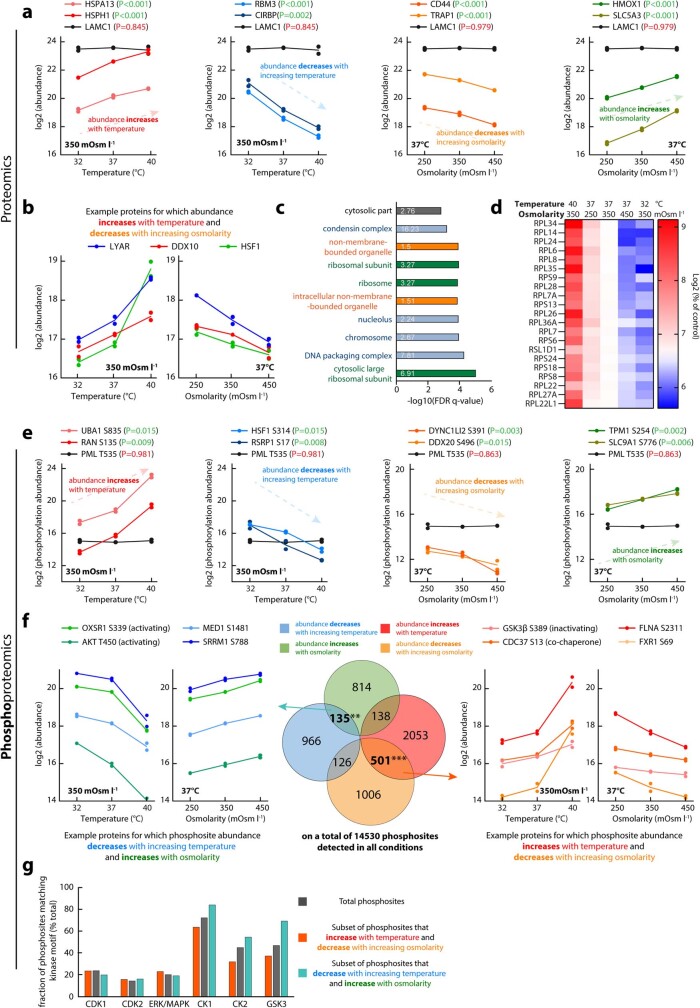
Proteome and phosphoproteome adaptation to sustained change in temperature or external osmotic potential. (**a**) Representative examples of proteins whose relative abundance correlates or inversely correlates with temperature or external osmolarity after two weeks adaptation. The adjusted p-value of linear fit by LIMMA was used to determine significance (with threshold p < 0.05), and is reported for the representative examples; Lamc1 is shown as an example of a protein whose abundance does not change with either osmolarity or temperature. Corresponds to Fig. 2b. (**b**) Examples of proteins whose abundance both increases with increasing temperature and decreases with increasing osmolarity, i.e. could be Ψ_π_-regulated. (**c**) Gene Ontology compartment enrichment for the 344 proteins that are oppositely regulated by temperature and osmolarity, against the background of all detected proteins. Number on the bars represents fold-change enrichment, colour of the bars highlights semantically close terms. (**d**) Validation of enrichment for ribosomal subunits in (**c**), strongly indicates a co-ordinated change in ribosomal subunit expression that is oppositely affected by temperature vs osmolarity during long-term adaptation, consistent with previous findings^96^. (**e**, **f**) Corresponding to Fig. 2d: representative examples of phosphopeptides whose relative abundance correlates or inversely correlates with temperature or osmolarity (**e**), as well as phosphopeptides that are oppositely regulated by temperature and osmolarity (**f**), i.e. change significantly with decrease (left) or increase (right) as a function of challenge to Ψ_π_. (**g**) Kinase motif prediction analysis. All detected phosphopeptide sequences were queried for matches to known phosphorylation consensus motifs for a panel of kinases (based on the PHOSIDA database); proportion of phosphopeptides matching motifs for the six selected kinases is presented, comparing overall levels (grey bar) to the subset of phosphopeptides putatively regulated by Ψ_π_ (proportion z-test: all differences are significant with adjusted p-value < 0.0001 for CK1, CK2, and GSK3, not significant for CDK1, CDK2, ERK/MAPK). Note that Ψ_π_-responsive phosphosites are enriched for motifs recognized by promiscuous kinases with established preference for IDRs (casein kinase 1, casein kinase 2, glycogen synthase kinase 3, see also refs. ^97,98^). Source data

We hypothesized that cells possess a fast-acting compensatory mechanism to preserve water availability and reasoned that since some proteins can strongly affect *Ψ*_*π*_ (Fig. 1a), this response could be mediated by proteins themselves. If true, we expected that proteins involved in water potential homeostasis would show consistent changes in expression or activity after long-term exposure to either thermal or osmotic stresses, to defend against any further perturbations from their new *Ψ*_*π*_ setpoint. Specifically, we sought to identify proteins and phosphoproteins of which the abundance varies not only with external osmotic strength, but also inversely with temperature, due to their antagonistic impact on the free:structured water ratio in concentrated colloidal solutions (Fig. 1b). To this end, confluent (quiescent) cultures of primary mouse fibroblasts were allowed to adapt over 2 weeks to conditions of lower/higher external osmolarity (±100 mOsm l^−1^) or temperature (32 °C or 40 °C). The cellular (phospho)proteome composition was then compared with the controls (37 °C, 350 mOsm l^−1^) using quantitative mass spectrometry (MS; Fig. 2a). Validating our approach, we observed expected changes in the abundance and/or phosphorylation of known heat-shock proteins (such as HSPA13 and HSPH1), cold-shock proteins (for example, CIRBP and RBM3) and osmoregulated proteins (such as HMOX1 and SLC5A3) for each relevant stressor, as reported previously^30–32^ (Extended Data Fig. 5 and Supplementary Tables 1 and 2).

We found a significant over-representation of proteins for which abundance and phosphorylation was both postively correlated with temperature and negatively correlated with osmotic strength (Fig. 2b, Methods and Extended Data Fig. 5b–f). Gene Ontology analysis of these putatively *Ψ*_*π*_-responsive proteins revealed significant enrichment for localization to membraneless organelles (MLOs), including the nucleolus (Extended Data Fig. 5c). Furthermore, querying published databases of phase-separating proteins confirmed significant enrichment of proteins known to participate in the formation of biomolecular condensates (*P* = 3.6 × 10^−5^; Fig. 2c). This suggests that protein condensation may be involved in the response and adaptation to changes in *Ψ*_*π*_.

MLOs are biomolecular condensates that behave as liquid–liquid phase-separated compartments and are associated with the presence of intrinsically disordered regions (IDRs) within their constituent proteins^33–35^. IDRs in solution have a greater effect on solvent entropy compared with soluble globular proteins because their higher ratio of surface area:volume requires proportionally more hydration water^36^. IDR-containing proteins, such as fused in sarcoma (FUS), can reversibly form condensates depending on their local environment and post-translational modifications^33–35^. The entropic cost of IDR hydration can be enthalpically compensated by electrostatic factors—such as phosphorylation—and, throughout the proteome, most protein phosphorylation indeed occurs within IDRs^37^. On the basis of previous research in yeast^38,39^, it therefore seemed plausible that global changes in IDR phosphorylation might provide one means for modulating the effect of intracellular proteins on *Ψ*_*π*_, thereby buffering *Ψ*_*π*_ against applied thermal or osmotic changes. Our analysis of the putative *Ψ*_*π*_-responsive phosphoproteome supported this paradigm: the relative proportion of IDR phosphorylation increased during adaptation to both hyperosmolarity and lower temperature, and vice versa (Fig. 2d). Notably, temperature had the opposite effects to external osmolarity on the phosphorylation of OXSR1 kinase, a key osmo-effector, at a known regulatory site within its IDR^40^ (Fig. 2e and Extended Data Fig. 5f). Saliently, *Ψ*_*π*_-responsive phosphosites were enriched for motifs recognized by promiscuous kinases with established preference for IDRs (casein kinase 1, casein kinase 2, glycogen synthase kinase 3; Extended Data Fig. 5g). Collectively these results show that chronic osmotic or thermal perturbations elicit similar adaptations in the (phospho)proteome that implicate MLOs and intrinsically disordered proteins as frontline defenders of intracellular *Ψ*_*π*_.

## Cellular control of condensates by *Ψ*_*π*_

Macromolecules within condensates are predicted to be less hydrated compared with in bulk solvent^8^. Given the involvement of IDR-containing proteins during osmotic and thermal adaptation (Fig. 2), and that condensation of the intrinsically disordered protein FUS releases entropically unfavourable hydration water in vitro^41^, we hypothesized that changes in biomolecular condensation could buffer intracellular water potential. For example, under acute hypoosmotic or hyperthermal challenge, a transient increase in free H_2_O molecules available for protein hydration could provide the bioenergetic drive to liberate IDR-containing proteins from condensates, hydration of which would proportionally decrease free water and thereby minimize the impact of the challenge on cellular water potential.

**Fig. 3:**
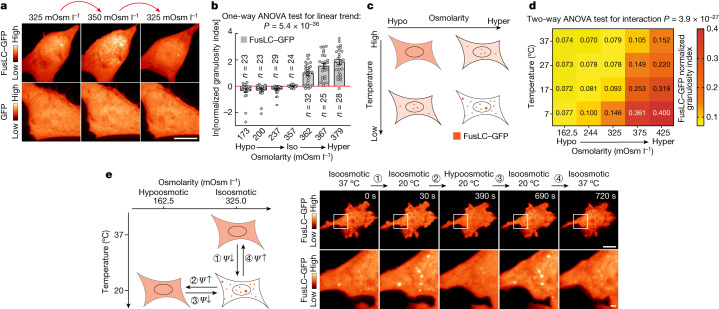
Duality of thermal and osmotic perturbation on FusLC condensation in cells. **a**, Representative maximum-intensity *z*-projections of SH-SY5Y cells transiently expressing GFP or FusLC–GFP, subjected to the indicated mild osmotic challenge, imaged using SDCM within 1 min of perturbation. Note that there is no condensation of GFP alone, and condensation of FusLC–GFP is reversible. Quantification is shown in Extended Data Fig. 8a,b. **b**, The change in the granulosity index for FusLC–GFP in U2OS cell nuclei after osmotic challenge (details of the deep-learning-based segmentation method are provided in the Methods). Data are normalized to granulosity per cell before challenge. Statistical analysis was performed using one-way ANOVA with test for linear trend; the *P* value is shown. **c**, Prediction if the change in external osmolarity affects FusLC–GFP condensation through changes in *Ψ*_*π*_, rather than directly: both low temperature and high osmolarity should induce condensation of FusLC–GFP in cells, but also compensate for each other. **d**, SH-SY5Y cells transiently expressing FusLC–GFP were changed to medium of the indicated osmolarity and imaged using SDCM. The temperature was then quickly shifted to lower values using microfluidics (Methods) while imaging. The FusLC–GFP condensation state was then automatically measured and plotted as a function of temperature and external osmolarity (mean granulosity index). Statistical analysis was performed using two-way ANOVA to test the interaction between temperature and osmolarity; *P* < 0.0001. *n* = 10–42 cells analysed per condition. **e**, The principle of the experiment (left). A single SH-SY5Y cell transiently expressing FusLC–GFP was moved along the temperature/osmolarity phase transition curve using dual-layer microfluidic chips (Methods), which permit the rapid change of temperature and/or the osmolarity of the medium while keeping the cell in focus on the microscope. Right, images of a representative experiment as described for the left panel (maximum-intensity *z*-projections using SDCM). High-magnification images of the area indicated by a white square are shown at the bottom. The elapsed time is indicated in seconds. For **a** and **e**, scale bars, 10 µm (**a** and **e** (top)) and 1 µm (**e** (bottom)). Source data

**Extended Data Fig. 6:**
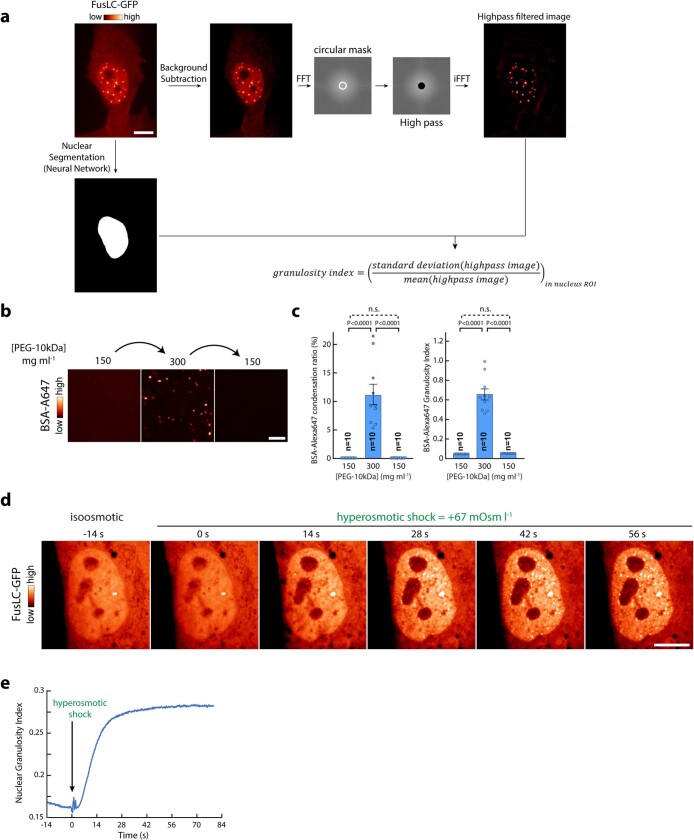
Imaging pipelines used for automated quantification of condensation in live cells. (**a**) Imaging pipeline to quantify the fraction of signal that is condensed in live cell experiments. The granulosity index is computed as follows: Raw images were processed for homogenous background subtraction, then Fast Fourier Transform (FFT), then high-pass filter via a circular mask and inverse FFT. The ratio between the standard deviation and the mean of the signal in the high-pass-filtered image is then computed in specific ROIs (for instance, the nucleus). Note that the granulosity index is measured in real space, while the condensation ratio is measured in the Fourier space. (**b**-**c**) Comparison of the results obtained by the granulosity index and the condensation ratio. BSA-Alexa-647 (1 µM) was shifted from 150 mg ml^−1^ PEG-10kDa to 300 mg ml^−1^ PEG-10kDa before dilution back to 150 mg ml^−1^ PEG-10kDa. The state of condensation of BSA was imaged by SDCM (b) and quantified (**c**; mean ± SEM of condensation ratio). For the granulosity index, the whole image was used as ROI. Statistics: one-way ANOVA followed by Tukey’s post-hoc test (P value indicated, n: number of images per sample). Note that the two methods give similar results. The images in (**b**) and the left panel of (**c**) are the same as in Extended Data Fig. 9f-g, reproduced here for convenience. (**d-e**) Illustration of the capacity of the above-described imaging pipeline to resolve dynamic changes in protein condensation in live cells. (**d**) U2OS cells transiently expressing FusLC-GFP were imaged live at high-speed by SDCM (single plane, stream at 140 ms per frame), and a hyperosmotic shock was induced after 100 frames. (**e**) The granulosity index of the nucleus was then computed over time. Note that condensation induced by hyperosmotic shock is fast (in the timeframe of seconds) and homogenous. See also Supplementary Video 2. Scale bar: 5 µm (**b**) and 10 µm (**a**,**c**).

**Extended Data Fig. 7:**
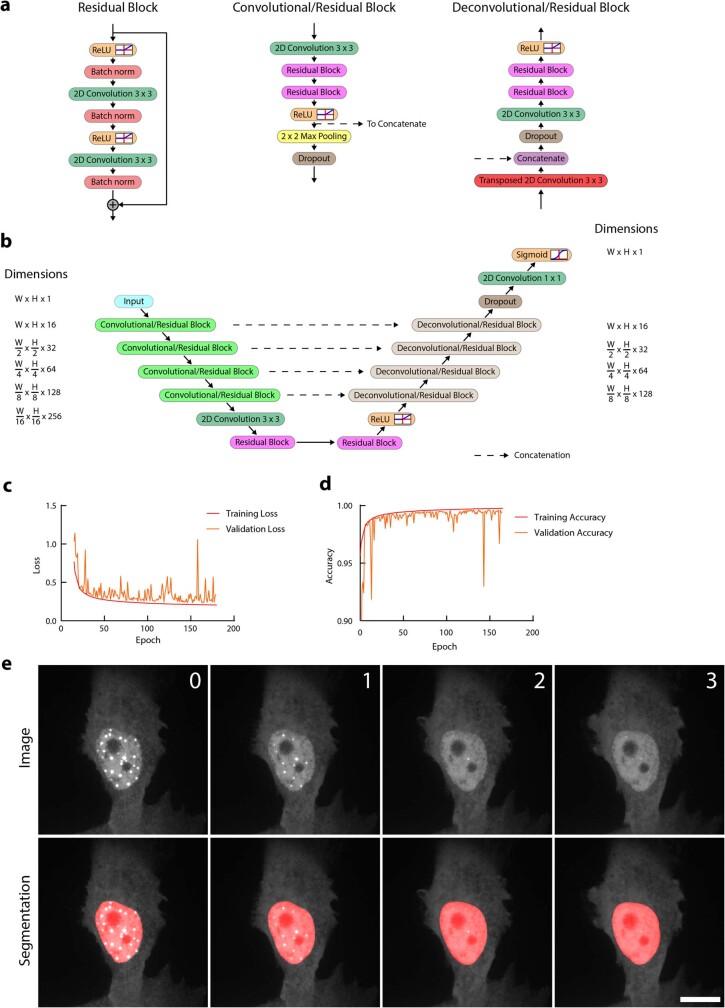
Description and validation of the neural network used to automatically segment the nucleus in FusLC-GFP images. (**a**,**b**) Overview of the network architecture used (see methods for more details). Briefly, the network has a fully connected convolutional ‘U-net’ (ref. ^82^) architecture, comprising a contracting stack of convolutional/residual blocks and an expansive path involving up-convolutions and concatenations from the contracting path. (**c**,**d**) Network training. The network was initially trained on a dataset of 132 SDCM images of SH-SY5Y cells transiently expressing FusLC-GFP and stained with Hoechst (maximum intensity Z-projections). The Hoechst signal was then thresholded to establish the nucleus ‘groundtruth’. After initial training of the network on the FusLC-GFP images, predictions were manually refined to yield a larger training dataset (598 images). This dataset was then used to retrain the network, until both the loss and accuracy had plateaued. Loss/accuracy curves from this second training run are depicted in **c** and **d**. The loss represents the binary cross entropy loss used to train the model. The dropout rate was set at 0.25 for the first and convolutional/residual block and the last deconvolutional/residual block, and 0.5 for all other blocks, and a batch size of 8 images was used. (**e**) Raw data (top panels) and overlay between raw data and nuclear segmentation (bottom panels) of SH-SY5Y cells transiently expressing FusLC-GFP during recovery from a transient (+50 mOsm l^−1^) hyperosmotic shock. Elapsed time in min. Scale bar: 10 µm. Source data

**Extended Data Fig. 8:**
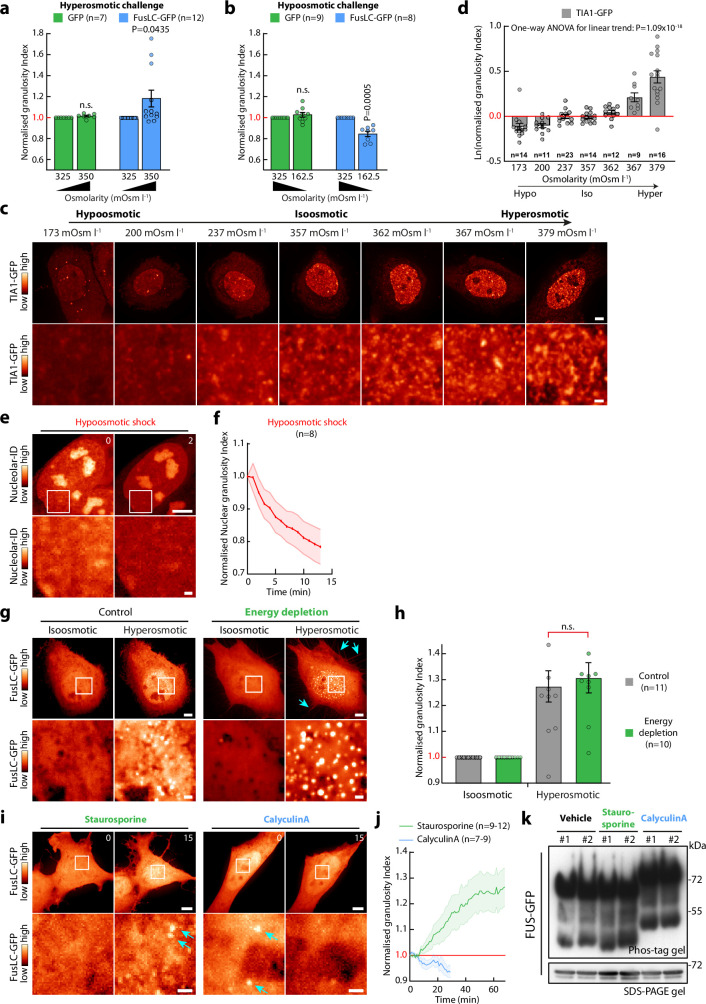
Condensation state of cellular marker as a function of osmotic challenge and changes in the global phosphorylation of the proteome. (**a**,**b**) SH-SY5Y cells transiently expressing GFP or FusLC-GFP were subjected to indicated mild osmotic challenge and the condensation level of GFP or FusLC-GFP was quantified (granulosity index, see methods) upon hyperosmotic challenge (**a**) or hypoosmotic challenge (**b**). Mean ± SEM (n: number of independent cells analysed per condition). Data were normalized to the value of the granulosity before the osmotic challenge in each cell. Statistics: one sample t-test with a hypothetical mean value of 1 (P value as indicated). (**c**,**d**) Condensation of TIA1-GFP in U2OS cells upon osmotic challenge. (**c**) U2OS cells transiently expressing TIA1-GFP subjected to indicated osmotic challenge and imaged by SDCM (images correspond to maximum intensity projections of entire cell). Data were denoised using a wavelet “à trous” filter (see methods). Bottom panels correspond to zoom cropped views in the nucleus of different cells in the same conditions. (**d**) Variation of the granulosity index (mean ± SEM) of TIA1-GFP in the nucleus of U2OS cells upon osmotic challenge (see methods). Data were normalized to the value of the granulosity before the osmotic challenge in each cell. Statistics: one-way ANOVA with test for linear trend (P value as indicated). (**e**,**f**) Nucleolar condensation in response to a hypoosmotic challenge. (**e**) SH-SY5Y cells were stained with Nucleolar-ID for 15 min before being exposed to hypoosmotic shock (325 mOsm l^−1^ to 162.5 mOsm l^−1^) and imaged by SDCM. Elapsed time after hypoosmotic shock indicated in min. (**f**) Mean granulosity index over time (±SEM) in the condition described in (**c**). n: number of cells analysed. Granulosity was measured in the nucleus after deep-learning based nucleus segmentation (see methods). (**g**-**h**) FusLC-GFP condensation upon hyperosmotic shock is a passive process. (**g**) U2OS cells transiently expressing FusLC-GFP were treated (or not) with an established energy-depletion medium then subjected to a hyperosmotic shock (see methods). Blue arrows indicate spiky protrusions characteristic of energy depletion^77^. (**h**) Granulosity index after the hyperosmotic shock normalized by its value in isosmotic conditions (mean ± SEM). Statistics, unpaired t-test, P = 0.7, n: number of cells analysed. FusLC-GFP condensation still occurs in energy-depleted cells. (**i**-**k**) FusLC-GFP condensation in response to global changes in protein phosphorylation. (**i**) SH-SY5Y cells transiently expressing FusLC-GFP were treated with 10 µM Staurosporine or 3 nM CalyculinA and FusLC-GFP condensation was monitored by SDCM at constant temperature and external osmolarity. Elapsed time after treatment indicated in min. Note the appearance of FusLC-GFP foci (arrows) upon global dephosphorylation by Staurosporin, and conversely, their disappearance upon global phosphorylation by CalyculinA. (**j**) Mean granulosity index over time (±SEM) in the condition described in (**i**). n: number of cells analysed. (**k**) SH-SY5Y cells transiently expressing FusLC-GFP were treated with Staurosporine, CalyculinA or DMSO vehicle for 50 min at 37 °C then FusLC-GFP was immunoprecipitated and analysed by GFP western blot after transfer from Phos-tag gels. Two technical replicates are shown. Note that FusLC-GFP runs at higher apparent molecular weight on the Phos-tag gel upon CalyculinA treatment, indicative of increased phosphorylation. Scale bars: 5 µm (**c**,**e**,**g**,**i** top panels); 1 µm (**c**,**e**,**g**,**i** bottom panels).

To test this hypothesis, we used FusLC–GFP—a fusion of the FUS N-terminal IDR with the GFP fluorescent reporter that is an established model for phase separation^33–35,41^. If formation or dissolution of biomolecular condensates acts to oppose variations in *Ψ*_*π*_, then one would expect a rapid increase in condensation after acute hyperosmotic challenge, releasing previously structured water into the bulk solvent to counteract the externally applied change. Our prediction was confirmed by experimental observation: a modest, transient increase in extracellular osmotic strength elicited a rapid (within seconds) and a reversible increase in FusLC–GFP condensation; by contrast, GFP alone showed no significant change and remained diffuse throughout (Fig. 3a,b; see the Methods and Extended Data Figs. 6 and 7 for automated, deep-learning-based quantification and Extended Data Fig. 6d,e and Supplementary Video 2 for automated analysis of the dynamics of FusLC–GFP condensation after hyperosmotic shock as an example). Conversely, decreased condensation was observed in response to hypoosmotic challenge (Extended Data Fig. 8a,b). In both cases, the level of condensation varied with the magnitude of the applied change (Fig. 3b) both for FusLC–GFP and TIA1–GFP, another well-characterized phase-separating protein (Fig. 3b and Extended Data Fig. 8c,d). To confirm that our findings were not attributable to protein overexpression, we considered a naturally condensed cellular structure, the nucleolus. The nucleolar condensation state was similarly responsive to an acute increase in extracellular osmolarity (Extended Data Fig. 8e,f), as previously reported^42^. Importantly, the hyperosmotic condensation of FusLC–GFP was similar in energy-depleted cells treated with an established medium containing 2-deoxyglucose and azide, suggesting that condensation can occur passively as the system rapidly restores water equilibrium after challenge (Methods and Extended Data Fig. 8g,h).

The effect of certain macromolecules on *Ψ*_*π*_ becomes nonlinear in concentrated solutions (for example, BSA, Hb, PEG), with a magnitude that is temperature dependent (Fig. 1a and Extended Data Fig. 1). If the same holds true for IDR- containing proteins in cells, an acute decrease in temperature should phenocopy an external hyperosmotic challenge due to their convergent effect on intracellular *Ψ*_*π*_. Without compensatory responses, both stimuli would rapidly decrease the free:structured water ratio, through net H_2_O efflux from cells for hyperosmotic challenge or increased size of hydration layers for hypothermal challenge (Fig. 1b). By extension, if changes in the macromolecular condensation truly act to defend *Ψ*_*π*_, then phase separation should increase as temperature decreases, liberating free water to counteract the temperature-induced change in water availability. Furthermore, hypo- and hyperosmotic challenges would be expected to attenuate and amplify, respectively, the effect on cellular *Ψ*_*π*_ caused by lowering temperature. These predictions were validated using FusLC–GFP: temperature decrease stimulated condensation that acted synergistically with hyperosmotic treatments, but was mitigated by hypoosmotic treatments (Fig. 3c,d). The highly significant interaction (*P* < 0.0001) between external osmotic strength and temperature for the condensation phenotype strongly indicates the interdependence of these two factors. In other words, osmotic and temperature challenges are not acting on Fus condensation independently, rather, the effect of an osmotic perturbation depends on temperature, as both affect the free:structured water ratio. We validated this by inducing rapid (around 5 s) temperature changes in a shear-stress-free manner, taking single cells along the thermo-osmotic phase transition curve while monitoring FusLC–GFP, using dual-layer microfluidics to simultaneously control both extracellular osmotic strength and temperature at a high precision and temporal resolution (Fig. 3e, Methods and Supplementary Video 1). Moreover, D_2_O attenuated the increase in FusLC–GFP condensation that was normally elicited by an acute increase in extracellular osmotic strength (Extended Data Fig. 4g,h), consistent with changes in condensation being *Ψ*_*π*_ mediated.

Finally, for any given *Ψ*_*π*_, if the extent of condensation is intrinsic to each protein’s surface biochemistry, then an increase in enthalpically favourable surfaces on FusLC–GFP should reduce its condensation level, whereas a decrease in surface charges should increase condensation. As with all phosphoproteins, the level of FusLC–GFP phosphorylation depends on the relative rate of phosphorylation versus dephosphorylation, both of which are readily amenable to pharmacological inhibition. Consistent with a previous report^43^, condensation was rapidly increased by acute broad-range kinase inhibition, whereas condensation was rapidly decreased by acute phosphatase inhibition, compared with the controls (Extended Data Fig. 8i–k). It has previously been suggested that phase separation relies on the balance of entropic and enthalpic thermodynamic driving forces^7^, but the biological importance and physiological function of biomolecular condensation in cells is subject to much debate^44^. Collectively, our observations indicate that rapid changes in protein condensation buffer intracellular *Ψ*_*π*_ against physiological fluctuations in temperature, osmolarity and, we predict, hydrostatic pressure: a built-in biophysical solution for the strict requirement to maintain *Ψ*_*π*_ over short timescales, enabling cells to homeostatically respond and adapt in the longer term.

## *Ψ*_*π*_ tunes condensate formation in vitro

**Fig. 4:**
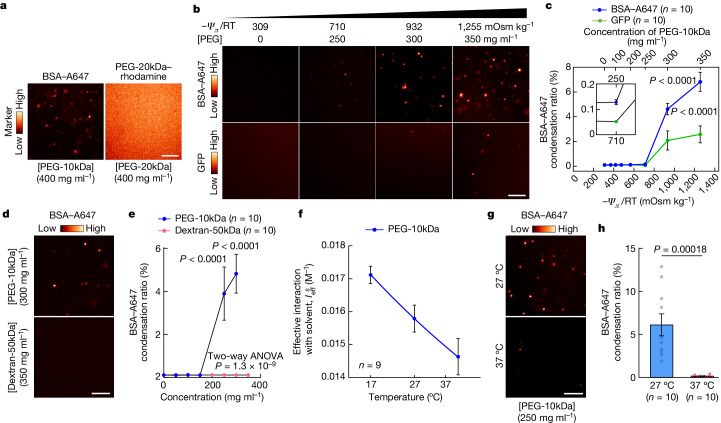
Protein condensation in solution rapidly responds to acute changes of *Ψ*_*π*_. **a**, The condensation state of BSA–Alexa Fluor 647 (A647) (1 µM) and PEG–20kDa-rhodamine (1 mg ml^−1^) in solutions in which free water availability was reduced by non-fluorescent PEG, assessed by SDCM (single confocal planes). Compared with BSA, PEG does not form condensates when the osmotic potential is low. **b**,**c**, Differential condensation of BSA and GFP as free-water availability decreases with increasing PEG concentration. BSA–A647 (1 µM) or GFP (1 µM) were imaged by SDCM (**b**) (27 °C) in PEG solutions of different osmotic potentials (Extended Data Fig. 1) and automatically quantified (**c**). For **c**, data are mean ± s.e.m. of the condensation ratio (Methods). Statistical analysis was performed using two-way ANOVA with Šídák’s test comparing BSA versus GFP; *P* values are indicated. The *n* values indicate the number of images per sample (Extended Data Fig. 9e). **d**,**e**, BSA condensation is not due to simple crowding effects. BSA–A647 (100 nM) was incubated at 27 °C in solutions of PEG (300 mg ml^−1^) or dextran (350 mg ml^−1^) of similar size, with the condensation state imaged by SDCM (**d**) and quantified (**e**). For **e**, data are mean ± s.e.m. Statistical analysis was performed using two-way ANOVA followed by Šídák’s test comparing PEG versus dextran; *P* values are indicated. *n* values indicate the number of images per sample. Dextran increases molecular crowding without causing BSA to form condensates, in contrast to PEG, which lowers free-water availability significantly more than dextran (Extended Data Fig. 1). **f**, Plot of ${I}_{{\rm{eff}}}^{s}$ (±95% confidence interval) versus temperature for PEG-10kDa. The *n* values indicate the number of independent osmometry curves fitted simultaneously to evaluate ${I}_{{\rm{eff}}}^{s}$ in each condition. PEG–solvent interactions increase as the temperature decreases (Extended Data Fig. 1). **g**,**h**, The effect of temperature on BSA condensation. BSA–A647 (100 nM) in PEG-10kDa (250 mg ml^−1^) at the indicated temperatures was imaged using SDCM (**g**) and the condensation ratio was calculated (**h**). For **h**, data are mean ± s.e.m. Statistical analysis was performed using Mann–Whitney *U*-tests. The *n* values indicate the number of images analysed. For **a**, **b**, **d** and **g**, scale bars, 5 µm. Source data

To investigate the capacity of protein condensation to buffer *Ψ*_*π*_ within the physiological range, we used the temperature-dependent nonlinear relationship of PEG with osmotic potential (see Extended Data Fig. 1 for measurements of ${I}_{{\rm{eff}}}^{s}$ as a function of temperature and polymer size). PEG has no solubility limit and does not phase separate across physiological temperatures (Fig. 4a). We therefore used PEG to generate low free:structured water ratios to examine the effect on protein–protein interactions in solution without requiring high concentrations of the protein itself.

**Extended Data Fig. 9:**
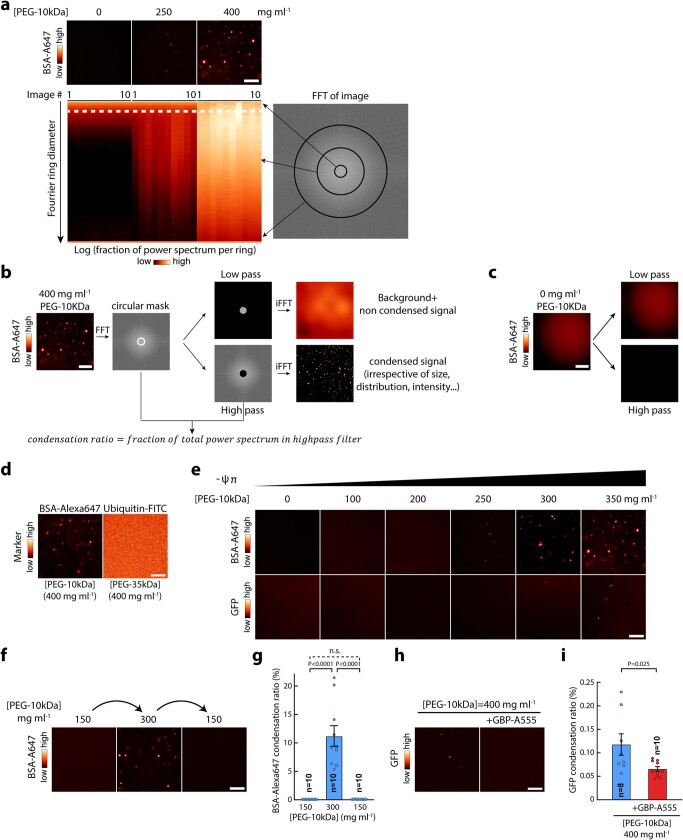
Imaging pipelines used for automated quantification of condensation in vitro, and associated controls. (**a**) Top panel: BSA-Alexa-647 (1 µM) in indicated PEG concentration imaged by SDCM. Bottom panel: the FFT was computed for the images presented in the top panel (right-hand panel), and the fraction of the power spectrum in rings of increasing diameter was measured and plotted on a log scale (left-hand panel, see methods). Each pixel-wide column corresponds to a single image to evaluate variability within the sample (10 images per PEG concentration). Note that an increased presence of spots in the image due to condensation results in higher signal in rings of larger diameters. Also note the similarity of the power spectrum of images in the same condition. (**b**) Imaging pipeline to quantify the fraction of signal that is condensed in in vitro experiments. Raw images were processed for FFT, then a circular mask was applied. The corresponding low pass image (after inverse FFT, iFFT) corresponds to the background and the signal from the non-condensed protein, while the high pass image corresponds to the signal from the condensed protein. The condensation ratio is defined as the fraction of the power spectrum in the high pass filter. (**c**) Low pass and high pass filtered image using the mask as in (**b**) but for an image without condensates (BSA-Alexa-647 (1 µM) alone), showing the absence of signal in the high pass image. (**d**) The state of condensation of BSA-Alexa647 (1 µM) and Ubiquitin-FITC (100 nM) in solutions where the availability of free water is reduced by non-fluorescent PEG (400 mg ml^−1^) was assessed by spinning disk microscopy. Images correspond to single confocal planes. The “PopRed” LUT was applied after the dynamic range was adjusted between minimum and maximum grey values of each images (note that the dynamic range was not kept identical between images). Note that the effect on osmotic potential of the three PEGs of different length is similar at the 400 mg ml^−1^ concentration used here (see Extended Data Fig. 1). Note also that the BSA-Alexa647 panel is the same as in Fig. 4a, reproduced here for comparison. On the contrary to BSA, Ubiquitin does not phase separate when the osmotic potential is high. (**e**) Differential condensation of BSA and GFP as a function of the decreased availability of free water when macromolecule concentration is increased. BSA-Alexa647 (1 µM) or GFP (1 µM) were imaged in indicated PEG solution at 27 °C by SDCM. (**f**,**g**) Reversibility of the condensation of BSA as a function of the availability of free water. BSA-Alexa-647 (1 µM) was shifted from 150 mg ml^−1^ PEG-10kDa to 300 mg ml^−1^ before dilution back to 150 mg ml^−1^, all at 27 °C. The state of condensation of BSA was imaged by SDCM (**f**) and quantified (**g**; mean ± SEM of condensation ratio) in each step. Statistics: one-way ANOVA followed by Tukey’s post-hoc test (P value indicated, n: number of images per sample). Note that (**f**) and (**g**) are the same data as in Extended Data Fig. 6b-c. (**h**-**i**) The state of condensation of GFP (100 nM) in PEG in the presence or absence of 100 nM anti GFP nanobody (GBP-Alexa555) was assessed by spinning disk microscopy (**h**) and quantified (**i**), see methods. Statistics: t-test (n: number of images analysed). Scale bars: 5 µm. Source data

BSA is a secreted soluble globular serum protein with surface-exposed IDRs^45^ that does not form condensates physiologically^46^. We therefore used BSA to test our hypothesis that *Ψ*_*π*_ is a primary determinant of protein condensation. Using microscopy, we observed that 1 µM fluorescently labelled BSA rapidly formed condensates at PEG concentrations that mimic macromolecule concentrations in the cytoplasm (300–550 mg ml^−1^)^47^, but not at concentrations found in the serum (60–80 mg ml^−1^) (Fig. 4b,c and Extended Data Fig. 9a–e; see the Methods for image-based quantitative measurements of protein condensation). We confirmed that BSA forms bona fide condensates through their rapid reversibility (Extended Data Fig. 9f,g). We also verified that condensation of BSA at high concentrations of PEG was not a consequence of macromolecular crowding or excluded volume, as a similar concentration of dextran, which has little effect on *Ψ*_*π*_ (Extended Data Fig. 1h), did not cause BSA to condense (Fig. 4d,e). Furthermore, BSA condensed more readily than GFP, which lacks IDRs (Fig. 4b,c), and ubiquitin, a protein with exceptionally enthalpically favourable surface interactions with water, which did not condense at any water potential that we could measure (up to 4,000 mOsm kg^−1^; Extended Data Fig. 9d). Moreover, prior incubation of GFP with stoichiometric amounts of an anti-GFP nanobody (Extended Data Fig. 9h,i) abolished GFP condensation at high PEG concentrations. Considering that, as circulating proteins, antibodies have evolved to be extremely soluble, this can be rationalized if nanobody binding masks part of GFP’s surface that is energetically unfavourable for hydration at a high PEG concentration. Nanobody binding thereby converts an unfavourable GFP into a favourable GFP–nanobody complex. This further supports the hypothesis that the *Ψ*_*π*_ at which condensation is energetically favourable is intrinsic to each protein’s surface biochemistry, where pH, ionic strength and post-translational modifications are secondary considerations that modulate the relative favourability of a given macromolecule’s electrostatic interactions with the solvent versus other solutes.

Density transitions such as biomolecular condensation and complex assembly are entropically unfavourable with regard to the participating macromolecules, and constitute a complex phenomenon that varies with protein and co-solute identity and concentration, but also temperature^33–35^. Reasoning from the solvent’s perspective provides a simpler conceptual framework—all of these variables affect *Ψ*_*π*_, which provides the major thermodynamic driving force for most macromolecular interactions in cells including condensation^7,8^. Considering the system as a whole, complex assembly and biomolecular condensation occur to maximize the entropy of the cell’s most abundant component, water, by burying unfavourable hydration surfaces. The free energy of surface hydration is affected by many factors, with hydrophobicity being dominant^5^. The entropic cost of macromolecular assembly, stimulated by temperature decrease or osmolarity increase, is redeemed by the liberation of unfavourable structured hydration water into the bulk solvent, counteracting the applied change to restore equilibrium. We tested this using an osmotically negligible BSA concentration (100 nM; Fig. 1a) at PEG concentrations at which water potential is most sensitive to temperature and a 10 °C temperature decrease elicits a large decrease in potential energy, reflecting the proportional reduction in entropically favourable free-water availability (Fig. 4f and Extended Data Fig. 1). Critically, at PEG concentrations at which BSA readily condenses at 27 °C, increasing the temperature to 37 °C all but abolished condensation (Fig. 4g,h). This is consistent with solvent thermodynamics being the primary driver of protein condensation, rather than the concentration of any specific macromolecule.

## Biomolecular condensation buffers *Ψ*_*π*_

**Fig. 5:**
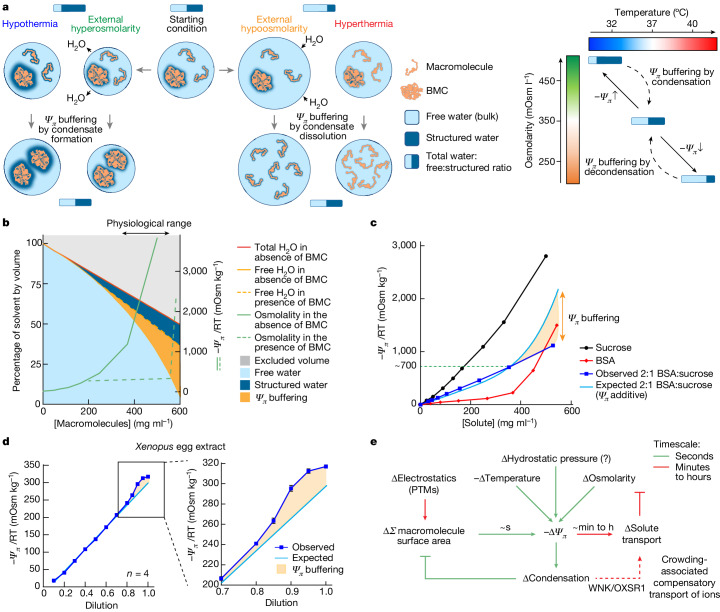
Macromolecular condensation buffers free-water availability. **a**, Schematic illustrating how changes in condensation elicited by *Ψ*_*π *_challenge would alter the free:structured water ratio to minimize the free-energy change. BMC, biomolecular condensate. **b**, After accounting for solute-excluded volume, the hydration of cytoplasmic macromolecules would result in a much lower free:structured water ratio (higher −*Ψ*_*π*_) than is observed physiologically. This is because most macromolecules participate in complexes and condensates, which minimizes their total solvent-exposed surface area. Considering those proteins for which physiological variation in *Ψ*_*π*_ alters the relative favourability of solvent versus macromolecular interactions, changes in the proportion of protein within biomolecular condensates effectively buffers *Ψ*_*π*_ by liberating or sequestering free water. Note that this simplified computation does not take small osmolytes into account. Further discussion is provided in Extended Data Fig. 10a–d. **c**, The change in the osmotic potential of BSA or sucrose solutions, and BSA:sucrose 2:1 co-solution, with solute concentration. Data are mean ± s.e.m. *n* = 3. **d**, The osmotic potential of dilution series in water of freeze-thawed extracts from *Xenopus* eggs. **e**, In concentrated colloidal solutions found within cells, physiological challenges to *Ψ*_*π*_ occur frequently through fluctuations in external osmolarity, temperature or hydrostatic pressure. Our findings suggest that changes in macromolecular condensation occur rapidly in response to *Ψ*_*π*_ challenge, minimizing the applied change in solvent thermodynamics by sequestering or liberating water within hydration layers. PTMs, post-translational modifications. Source data

**Extended Data Fig. 10:**
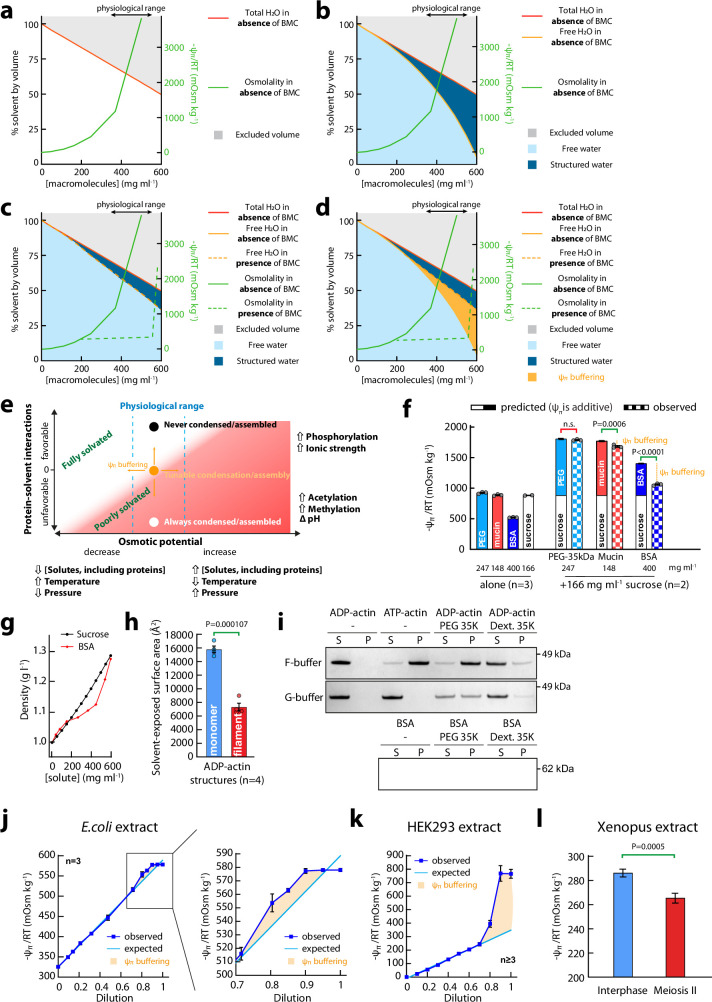
Osmotic potential buffering by proteins in cells. (**a**) The relationship between macromolecule concentration, excluded volume and osmotic potential for PEG-20kDa as a model protein is shown, incorporating direct measurements^99^. (**b**) The proportion of structured water was estimated by assuming 2373 water molecules per molecule of PEG, equivalent to a hydration shell that extends ~1 nm from each macromolecular surface^100^, and that each water molecule can be either free or structured at any moment. (**c**) Dotted lines represent a qualitative approximation of the predicted effect of biomolecular condensation (BMC) on the system. In this case, the relative favourability of solute-solute over solute-solvent interactions increases with osmotic potential. The result is that condensation increases more gradually as the concentration of macromolecules increases compared with if all macromolecules remained fully hydrated. (**d**) The difference in how free H_2_O (and osmotic potential) change as a function of [macromolecules] due to condensation is represented, i.e., the proportion of structured water in cells changes quite modestly over the physiological range of [macromolecules] due to a progressive increase in solute-solute interactions. Please note that, in modelling the colloidal osmotic potential of the cytosol, we use PEG to approximate the consequence of every macromolecular surface being solvent-exposed, where cytosol normally contains at least 350 mg ml^−1^ protein (equivalent to >3 moles l^−1^ of free amino acids). In these calculations, however, we do not consider any other osmolyte. This is obviously a simplification, since cytosol also contains >150 mM small osmolytes. This would further increase the >2000 mOsm kg^−1^ that would be expected if the cytosol were composed from PEG by at least an additional 150 mOsm kg^−1^ (osmolytes follow the van’t Hoff equation, unlike non-ideal proteins). This further illustrates how cells achieve the measured, physiological osmotic potential of the cytosol (~300 mOsm kg^−1^), by folding macromolecules and assembling them into higher order structures and condensates that minimize their total solvent-accessible surface area. In other words, buffering of intracellular water potential through macromolecular assembly and condensation is the only way such a high concentration of heterogeneous proteins can be maintained whilst remaining enzymatically active. Our observations in this investigation simply suggest that, for some proteins, the free energy difference between condensation and full hydration is so close to zero that they function as buffers of water potential upon physiologically-relevant challenge. (**e**) Whether by complex assembly or biomolecular condensation, the osmotic potential at which any given protein partakes in homo- or heterotypic interactions with other macromolecules depends on the favourability of its interactions with H_2_O, and so is sensitive to factors that affect Ψ_π_ (x-axis). Protein-solvent interactions can be modulated by changes in surface electrostatics (y-axis), through phosphorylation^38,63^ or histidine protonation^57^, for example. (**f**) Osmotic potential buffering by macromolecular phase separation. The osmotic potential of indicated mixtures of macromolecules was determined at 27 °C and compared to their expected values (sum of the osmotic potential of the constituents). Statistics: two-way ANOVA followed by Šídák’s post-hoc test (P value indicated, n: number of repeats). Compared with PEG, which does not phase separate (see Fig. 4a), intermediate concentrations of BSA effectively ‘buffer’ the osmotic potential of the solution against a further increase in osmotic potential (also seen with Mucin to a lower extent). (**g**) Density of BSA and sucrose solutions at indicated concentration. (**h**) Comparison of solvent-exposed surface (mean ± SEM) between filamentous and monomeric ADP-actin. Statistics: paired t-test comparing ADP-actin structures available in the PDB (n = 4, pdb codes 4A7N, 5ONV, 7BT7, 8A2Z). (**i**) Top two panels: Actin pelleting assay in indicated buffer conditions using 0.7 µM actin, which is above the Critical concentration (Cc) for ATP-actin, but below that of ADP-actin. As a negative control, the same pelleting assay was performed with 0.7 µM BSA in F-buffer (bottom panel). Depleting free water by addition of PEG triggers ADP-actin polymerization below the Cc. This is not a simple crowding effect, as it is not observed with dextran of the same size at the same concentration, (see Extended Data Fig. 1h). Note that the effect is observed in both G- and F-buffer, albeit to a lower extent in G-buffer. (**j**) left: Osmotic potential of dilution series with extracts from *E. coli* Right: magnified view of inset in left panel (mean ± SEM; n: number of independent dilution series). (**k**) Osmotic potential of dilution series with neat extract of HEK293 cells (mean ± SEM; n: number of independent dilution series). (**l**) Osmotic potential of cell-cycle arrested xenopus extracts measured at indicated point of the cell cycle (mean ± 95% Confidence Interval). To ensure precise measurements, a dilution series was measured, and the linear part of this curve was used to estimate the value of the undiluted extract (see methods). Statistics: Welch t-test (n = 2 dilution series per condition with at least 7 datapoints per dilution series). Note that panels (**j**,**k**) and all other such measurements in this manuscript were obtained by vapour-pressure osmometry, while panel (**l**) was measured by freezing-point-depression osmometry. Source data

Our observations suggest that changes in biomolecular condensation may function as a biophysical feedback mechanism between *Ψ*_*π*_ and solvent-exposed macromolecular surface area to buffer intracellular water availability against perturbation (Fig. 5a,b; see Extended Data Fig. 10a–d for sequential construction of the graph presented in Fig. 5b). This buffering capacity may be inherent to any IDR-containing protein for which the entropic penalty incurred by structured hydration water is finely balanced by the enthalpic gain from favourable water–protein surface interactions, and environmental conditions or surface modifications can switch these small thermodynamic margins (Extended Data Fig. 10e). Conversely, proteins with very favourable surface interactions with water, such as highly glycosylated mucin, would not be expected to phase separate under physiological conditions.

To experimentally demonstrate that phase-separating proteins can alter the free:structured water ratio, we set out to measure the amount of additional free water that becomes available when condensates are formed. We analysed the water potential of dilution series with one solute that does not phase separate, sucrose, and one that does, BSA (Fig. 5c (black curve and red curve, respectively)). If the protein condensation does not return structured water molecules to the free bulk solvent, then the water potential of a 2:1 co-solution between BSA and sucrose should be additive (Fig. 5c (light blue curve)). The observed water potential of the co-solution was significantly less negative than expected (Fig. 5c (dark blue curve)) above the critical threshold of around 700 mOsm kg^−1^ at which we start to observe BSA condensation in solution (Fig. 4c), reflecting a proportional increase in free-water availability. In essence, sucrose pushes the *Ψ*_*π*_ of the co-solution beyond the condensation threshold for BSA, leading to the formation of condensates and the release of structured hydration water into the free bulk. Further supporting this conceptual framework, the water potential of a sucrose–mucin co-solution deviated from the additive prediction by only 5% compared with 25% for IDR-containing BSA (Extended Data Fig. 10f). PEG does not form condensates (Fig. 4a) and the water potential of sucrose–PEG co-solutions followed the expected additive prediction (Extended Data Fig. 10f). Moreover, there was an inflection in the observed density of BSA solutions between 200–400 mg ml^−1^, at which increased BSA concentration had a modest impact on density compared with more dilute or concentrated solutions either side (Extended Data Fig. 10g (red curve)). By contrast, the density of simple solutions such as sucrose varied linearly with solute concentration as expected (Extended Data Fig. 10g (black curve)).

To test the generality of our findings, we examined whether *Ψ*_*π*_ could be buffered by other macromolecular assembly transitions besides phase separation. It is well established from crystallography studies that conversion from tertiary to higher-order structures, during filament formation, for example, can be induced by depleting free water. This is because higher-order structures have less solvent-exposed surface and therefore require less hydration water per monomer. In other words, reduced water availability renders higher-order structures the most energetically favourable state. For example, ADP–actin polymerization is not energetically favourable below the critical concentration at either end when water is not limiting. However, below the critical concentration, we found that incubation of ADP–actin with 100 mg ml^−1^ PEG-35kDa was sufficient to drive its polymerization, consistent with previous findings^48^ (Extended Data Fig. 10h,i), whereas 100 mg ml^−1^ dextran-35kDa did not, discounting simple crowding or excluded volume explanations. This is consistent with the fact that filamentous actin has half the solvent-exposed surface area per monomer compared with globular actin (Extended Data Fig. 10h). Thus, in theory, any macromolecular assembly process can buffer *Ψ*_*π*_. This being said, it is probable that IDR-containing proteins are the major effector of *Ψ*_*π*_ buffering in cells. Indeed, under physiological conditions, the finely balanced entropic cost of their hydration versus condensation renders them metastable, and also tuneable by post-translational modifications, and so well-suited to this function (Extended Data Fig. 10e).

We have provided strong correlational evidence that biomolecular condensation collegially performs a fundamental physiological function: buffering intracellular water potential to maintain optimum water availability. Given the essential roles, high abundance and very large number of different IDR-containing proteins in cells, it is impossible to formally prove that rapid changes in the global level of biomolecular condensates enable cells to accommodate acute physiological fluctuations in temperature, osmolarity and, presumably, hydrostatic pressure. However, it is possible to test a non-intuitive prediction informed by this hypothesis: that isolated cytoplasm would inherently resist applied changes to its water potential. To test the *Ψ*_*π*_ buffering capacity, we prepared neat cytoplasmic extract from *Xenopus* eggs, human embryonic kidney cells and *Escherichia coli* cells, and measured the change in water potential after dilution with pure water. Without inherent buffering, the water potential would linearly increase in proportion to the dilution. Conversely, *Ψ*_*π*_ buffering involving biomolecular condensates predicts a departure from linearity, as available free water would proportionally increase after cytoplasmic dilution and drive immediate compensatory dissolution of condensates. Such dissolution would subsume additional free water into hydration layers of newly released IDR-containing proteins, thus preserving the free:structured water ratio and resisting change in *Ψ*_*π*_ after dilution. We consistently observed the latter (Fig. 5d (*Xenopus*) and Extended Data Fig. 10j,k (*E. coli* and human embryonic kidney cell extracts)). Notably, we found the osmotic potential of fresh interphase *Xenopus* egg extracts was significantly lower than during mitosis (Extended Data Fig. 10l), when global increases in proteome phosphorylation are also observed, suggesting the possibility of dynamic post-translational *Ψ*_*π*_ regulation during cell division. Thus, isolated cytoplasm robustly buffers *Ψ*_*π*_, most likely through a rapid biophysical feedback response involving biomolecular condensation (Fig. 5e).

**Extended Data Fig. 11:**
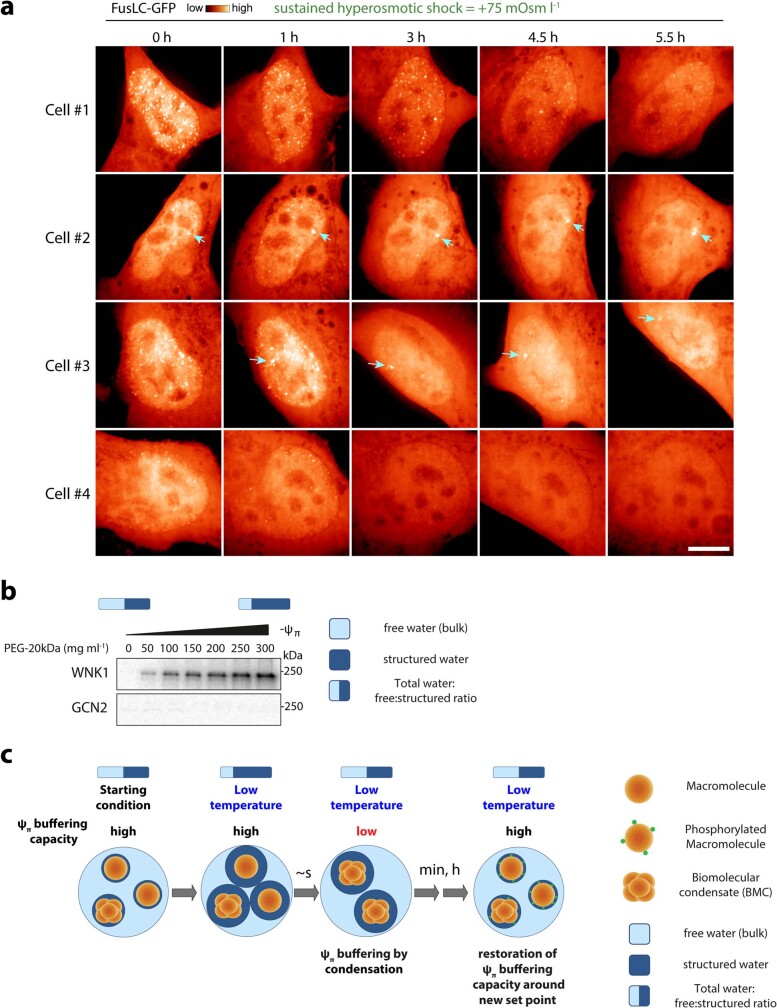
Timescales of osmotic potential buffering. Gradual changes in temperature, external osmolarity, and the intracellular concentration of soluble cytosolic macromolecules are countered by the active transport of small solutes, which maintain Ψ_π_ without appreciable volume change^2^. Over shorter timescales, severe external osmotic stress stimulates net movement of water over the plasma membrane and accompanying volume adjustments, with cell volume eventually restored by the concerted activity of various solute transport systems (so called Regulated Volume Increase or Decrease, RVI/D)^21^ in the longer term. We propose that rapid changes in biomolecular condensation of many different IDR proteins is the mechanism by which cells accommodate acute physiological fluctuations in temperature, hydrostatic and osmotic pressure, that normally help to maintain cell volume homeostasis. The following addresses the likely mechanisms that operate over longer timescales, where an acute challenge to intracellular water potential is sustained, supported by some relevant observations. (**a**) Timescale of relaxation of condensation upon sustained challenge to intracellular Ψ_π_ by a mild external hyperosmotic shock. Human U2OS cells transiently expressing FusLC-GFP were subjected to a sustained +75 mOsm l^−1^ hyperosmotic treatment and the degree of FusLC-GFP was assessed qualitatively by live SDCM over long timescales (see methods). The behaviour of four different cells is shown. Notice the heterogeneity of the long-term adaptation of cells: condensates in some cells completely dissolved within one hour (cell #4), while it can take >5 h for other cells (cell #1). This is expected as the level of protein condensation is a continuous variable, not a binary process, that results from changes in the equilibrium between fully hydrated and co-localized, partially hydrated monomers, and thus variations are expected in the cell population over long timescales according to differences in cell cycle or metabolic activity for instance. Also note that some condensates remain stable even when all the other condensates in the cell have disappeared (blue arrows). Nevertheless, the timescale of relaxation of phase separation is consistent with established mechanisms of osmoregulation and the behaviour of other IDR proteins^43,50,78,79^. Images correspond to maximum intensity z-projections (20 planes; Δ0.75 µm). Scale bar: 10 µm. (**b**) In vitro assays of kinase activity for purified WNK1 and General Control Nonderepressible 2 (GCN2) kinases upon varying osmotic potential via addition of indicated concentration of PEG-20kDa. In contrast with the GCN2 control, WNK1 activity increases with osmotic potential, as the free:structured water ratio decreases (see refs. ^2,49,101^ for more details). (**c**) Proposed model of short- and long-term buffering of water potential in cells using a rapid but sustained temperature decrease as an example. At short time scales (sub-second), protein condensation quickly buffers the free:structured water ratio in the cytosol whilst maintaining cell volume. At this point, the cell has reduced capacity to buffer Ψ_π_ and volume against further hypothermal or external hyperosmotic challenges, where cessation of the challenge would allow a rapid (passive) return to the starting condition. If the challenge is maintained (>minutes), it will elicit a change in the activity of Ψ_π_-sensitive proteins, such as WNK1 kinase whose auto-phosphorylation is stimulated by a fall in free:structured water ratio, leading to increased phosphorylation and activity of substrate effectors such as OXSR1/SPAK kinases^2,21^. The resultant change in phosphoproteome composition acts to restore Ψ_π_-buffering capacity around a new set point by: 1) phosphorylation of Ψ_π_-buffering proteins observed by mass spectrometry (Fig. 2, Extended Data Fig. 5); 2) compensatory electroneutral transport of small solutes via changes in transporter activity e.g. SLC12A family members are regulated by WNK-OXRS/SPAK signalling^2,21^; 3) longer term changes in gene expression and proteome composition. Together these processes function to restore the condensation state of the Ψ_π_-buffering proteins, and therefore the ability of the cell to buffer around the new osmotic setpoint that has been established. Challenges that exceed the cell’s innate Ψ_π_-buffering capacity are expected to additionally result in a cell volume change that is resolved by the same established mechanisms^2,29^. This framework of thinking is fully consistent with the recent results from Boyd-Shiwarski and colleagues^50^. Source data

After a sustained challenge to *Ψ*_*π*_, cells must restore their buffering capacity around the new setpoint that has de facto been established (Fig. 2). Under sustained hyperosmotic treatment, some cells exhibit qualitative relaxation of FusLC–GFP over time (Methods and Extended Data Fig. 11a). This is quite variable within the population, but generally occurs gradually over hours, a timescale that is consistent with established mechanisms of osmoregulation and the behaviour of other IDR proteins^43^. These slow recovery dynamics (hours) are in sharp contrast to the fast dynamics of condensation (seconds; Extended Data Fig. 6d,e). The mechanisms facilitating the return to baseline levels of condensation probably involve concerted phosphorylation of *Ψ*_*π*_-buffering proteins (Fig. 2), and phosphorylation-mediated changes in the activity of solute transporters such as SLC12A and VRAC by *Ψ*_*π*_-sensitive kinase/phosphatases, as well as longer-term changes in (phospho)proteome composition (Extended Data Fig. 5). In favour of this hypothesis, it was recently reported that WNK kinase activity is regulated by water potential rather than crowding or ion concentrations^2,49^ (Extended Data Fig. 11b). By our model, a decrease in temperature and/or hyperosmotic conditions would increase WNK kinase activity and the phosphorylation of its targets over time. Indeed, OXSR1, a well-established target of WNK^2^ and key effector of regulatory volume changes in response to acute osmotic perturbation, showed activating Ser339 phosphorylation after sustained challenges of *Ψ*_*π*_ that is entirely consistent with this model (Fig. 2e), as well as recent observations on WNK^50^. Progressive IDR phosphorylation would facilitate the dissolution of proteins from condensates during sustained hypothermal or hyperosmotic challenge (Extended Data Fig. 10e), and thereby restore the buffering capacity of the system (Extended Data. Fig. 11c).

## Conclusion

Water is often described as the matrix of life, in which biological molecules influence the structure and dynamics of surrounding water^51^. In cells, the potential energy of water is therefore distributed between structured water within hydration layers and free bulk solvent. The dynamics and availability of water within the crowded cytosol are not well characterized^7^. By considering the impact of temperature on the free:structured water ratio, we predicted and demonstrated the equivalence of thermal and osmotic perturbations on water thermodynamics within concentrated macromolecule solutions, with far-reaching consequences for cellular activity, viability and adaptation (Figs. 1 and 2).

Aqueous environments support complex molecular behaviours such as macromolecular assembly and phase separation^52,53^. Recent studies of biomolecular condensation have investigated the thermodynamics of phase transitions and the specific nature of macromolecules involved in this process, but the physiological relevance of phase separation in cells is hotly debated^44,54,55^. Our findings provide insights into both: the water potential ultimately drives the formation/assembly and dissolution/disassembly of protein condensates and multiprotein complexes, as previously suggested^7^ but, critically, this process impacts the water potential by releasing and sequestering free water, respectively. Osmotic imbalance and temperature extremes have posed existential threats since membrane-bound protocells first emerged, resulting in the evolution of conserved osmoregulatory and thermally responsive mechanisms^20,21^. Given its faster assembly kinetics compared with higher-ordered complex formation, we propose that biomolecular condensation is the primary buffer of intracellular *Ψ*_*π*_, providing cells with a thermodynamically driven rapid defence against acute fluctuations in osmotic strength, temperature and pressure that does not require specific osmosensors. Cellular proteins for which the equilibria between free and condensed states lie within the physiological *Ψ*_*π*_ range thereby act as a first line of defence, buying time for the cell to respond through post-translational regulation of solute transport systems (Fig. 5e and Supplementary Video 3 (summary animation)). In support of this model, dynamic changes in protein condensation counterbalanced physiologically relevant challenges to *Ψ*_*π*_ (Figs. 3 and 4), and the cytosol of different model systems showed inherent buffering capacity (Fig. 5). Widespread changes in osmotic composition, as well as protein assembly, condensation and solubility are observed under stress conditions and during ageing, as well as over the circadian cycle^2,3,56,57^. Future research should examine buffering capacity in stressed and aged cells, and test whether sensitivity to *Ψ*_*π*_ challenge varies with the time of day in healthy cells.

Importantly, we do not discount thermodynamic idiosyncrasies or specific functional roles for the reversible condensation of individual proteins. Rather, considering all proteins for which the energetics of macromolecular condensation/assembly lie close to parity, our findings suggest a global, collegial function to maintain intracellular *Ψ*_*π*_ that echoes Le Chatelier’s principle. In other words, changes in condensation occur to minimize the perturbation to the thermodynamic equilibrium of the system as a whole. When an osmotic or thermal perturbation is sustained over minutes, we predict that post-translational modification of IDRs acts in concert with well-characterized mechanisms of cell volume and osmotic homeostasis, such as *Ψ*_*π*_-sensitive WNK/OXSR1 signalling^2^, to restore water potential buffering capacity (Fig. 5e and Extended Data Fig. 11). Over longer durations, changes in gene expression and protein surface biochemistry (for example, phosphorylation) modify proteome composition to ensure that an appropriate panel of IDR-containing proteins can defend cellular *Ψ*_*π*_ around a new homeostatic set point (Fig. 2). The cell’s intrinsic *Ψ*_*π*_-buffering capacity arises from those IDR and other protein–protein interactions for which favourability is finely balanced with that of hydration. This may explain why many macromolecular assemblies are difficult to purify or reconstitute in solution and account for the ubiquity of IDRs throughout the tree of life^58^.

We propose that water potential and availability provide a unifying signal that communicates temperature and intracellular macromolecular status and, therefore, is essential for the cell’s intrinsic capacity to accommodate and survive external stressors.

## Methods

### Plasmids

The plasmid encoding GFP-tagged TIA1 was obtained from Addgene (106094). The plasmid for bacterial expression of the anti-GFP nanobody, named GBP (for GFP-binding peptide) was previously described^59^. To generate a plasmid encoding GFP-tagged FUS low-complexity domain (noted FUS-LCGF; corresponding to the FUS reference sequence NM_004960.4), purified RNA from human SH-SY5Y cells was reverse-transcribed using a FUS-specific primer (5′–3′, CGCCGCCGCCACCACTGCC). cDNA was then PCR-amplified using FUS low-complexity-spanning forward and reverse primers with EcoRI and BamHI sites, respectively (forward-EcoRI, 5′–3′, ATCTATGAATTCGCCACCATGGCCTCAAACGATTATACCCAAC; reverse-BamHI, 5′–3′, ATATGGATCCCCTCCACGGTCCTGCT). After EcoRI/BamHI digestion, the FUS low-complexity fragment was cloned into the pEGFP-N1 vector (BD Biosciences; GenBank: U55762). FUS-LC-EGFP plasmid was verified by Sanger-sequencing.

### Protein and reagents

Unless stated otherwise, reagents and purified proteins were purchased from Sigma-Aldrich. Dextran-35kDa was from *Leuconostoc mesenteroides* (Sigma-Aldrich, D1662). Rhodamine-PEG-20kDa was from Creative PEG works. FITC-labelled ubiquitin was purchased from Thermo Fisher Scientific. Tag-free GFP was expressed and purified as described previously^59^.

To generate BSA–A647, purified BSA (4 mg ml^−1^ in 0.1 M sodium bicarbonate, pH 8.3) was reacted for 1 h with a tenfold molar excess of Alexa Fluor 647 NHS Ester (Thermo Fisher Scientific, A20006). Excess dye was removed using the Zeba Spin column (Pierce) equilibrated in 20 mM HEPES, 150 mM KCl, pH 7.6.

To generate GBP–A555, purified GBP (0.5 mg ml^−1^ in 0.1 M sodium bicarbonate, pH 8.3, purified as previously described)^60^, was reacted for 1 h with a fourfold molar excess of Alexa Fluor 555 NHS Ester (Thermo Fisher Scientific). Excess dye was removed by a homemade G25 column equilibrated in PBS. Protein was flash-frozen in liquid nitrogen and stored at −80 °C.

### Osmometry

Vapour pressure osmometry was performed on the Vapro 5600 (ELITechGroup) system according to the manufacturer’s instructions, with temperature controlled by varying the ambient room temperature. The instrument was allowed to equilibrate to ambient temperature overnight, and the reading stability was validated before measurements were performed. Osmometry readings were assessed for solutions of varying composition and confirmed to be normally distributed with the following tests: Anderson–Darling, D’Agostino–Pearson, Shapiro–Wilk, Kolmogorov–Smirnov. A buffer containing 20 mM Tris-HCl pH 7.4, 150 mM KCl was used for preparation of all solutions and dilution series, except for *Xenopus* and human cell extracts, which were diluted with pure water because their neat osmotic potential was similar to that of the buffer. Vapour pressure osmometry of *Xenopus* extracts (Fig. 5d) was performed on thawed frozen samples (that is, not fresh). Human cell extracts and dilutions were maintained on ice until immediately before each reading. Stock macromolecular solutions were incubated overnight at 37 °C, with agitation at 300 rpm, to ensure complete dissolution before measurements were performed, and used to make dilution series. For the main figures, presented values were baseline-corrected by subtracting the mean osmotic potential of the buffer during each set of osmometry measurements; whereas, absolute osmotic potential values are shown in the Extended Data Figures. The error of predicted values of the osmotic potential of sucrose mixtures (Extended Data Fig. 10f) was computed by error propagation (that is, the square root of the sum of the squares of the s.e.m. of relevant samples). Osmotic potential data were fitted using MATLAB 2020b (Supplementary Discussion 1).

Osmometry measurements of fresh, cell-cycle arrested, *Xenopus* egg extracts (Extended Data Fig. 10l) were performed using the Osmomat 3000 freezing-point-depression osmometer (Gonotec). Before measurements on extract samples, a three-point calibration of the osmometer was performed using ultrapure water and certified 100 and 300 mOsm kg^−1^ solutions. To ensure accurate measurement of the difference between the two cell cycle states, we measured the osmotic potential across a dilution series, and extrapolated values from the part of the curve that shows linear behaviour (the osmotic potential as a function of dilution shows nonlinearity, as expected because the cytosol is composed of a mixture of ideal and non-ideal proteins like BSA; Extended Data Fig. 1). Undiluted extract was kept in a water bath at 18  °C or on ice during preparation of dilutions, and we did not observe a systematic influence of the measured osmotic pressure on the incubation temperature before measurement. To reduce the time spent in a diluted state before measurement, each dilution was prepared individually immediately before measurement, typically during the measurement of the preceding sample. The samples were prepared at the desired dilution by addition of ultrapure water and extract to a final volume of 35 µl in a fresh osmometer tube, and pipette-mixed with a P200 tip until visually homogeneous. Care was taken to avoid the introduction of small air bubbles in the solution during preparation. Between measurements, the osmometer probe was wiped twice with a dry Kimwipe, rinsed with ultrapure water and wiped twice again with a fresh Kimwipe. Dilutions were prepared and measured in non-sequential order (for example, 100%, 50%, 10%, 90%, 20% and so on) to avoid superimposing any potential time dependence to the titration data.

Note that, to measure the free-water buffering capacity of extracts (Fig. 5d and Extended Data Fig. 10j,k), it is important to use a vapour-pressure osmometer rather than a freezing-point-depression osmometer, to ensure that temperature changes during measurement, which affect condensation, do not affect the results.

### Cell culture

SH-SY5Y cells (Sigma) were maintained in Dulbecco’s modified Eagle’s medium (DMEM) supplemented with 20% fetal bovine serum (FBS), 1× non-essential amino acids (Gibco) and penicillin–streptomycin (100 U ml^−1^) at 37 °C and 5% CO_2_. Human U2OS osteosarcoma cells (ATCC) or mouse primary lung fibroblasts were maintained in DMEM (Thermo Fisher Scientific) supplemented with penicillin–streptomycin (100 U ml^−1^) and 10% Hyclone Fetalclone II or 10% Hyclone III, respectively. GlutaMAX (Gibco) was used when medium did not contain glutamine for long-term culture. For transient transfections, cells were transfected with Lipofectamine-2000 (SH-SY5Y cells) or −3000 (U2OS cells) 24 h before an experiment, according to the manufacturer’s instructions. For cell viability analysis, lymphoblastoid Raji cells^61^ (gift from P. Farrell, described previously^62^) or human foreskin fibroblasts (HFFs, from ATCC) were cultured in RPMI or DMEM, respectively, with 10% FCS, GlutaMAX (Gibco) and penicillin–streptomycin in a 37 °C humidified incubator with 5% CO_2_. All cell lines were tested and found negative for mycoplasma contamination.

### Cell extracts

To generate concentrated bacterial extracts (Extended Data Fig. 10j), BL21 bacteria were grown overnight in Terrific Broth medium at 25 °C. A bacteria pellet corresponding to around 20 ml was washed once with 20 mM Tris, 150 mM KCl, pH 7.4, then resuspended in 7 ml of 20 mM Tris, 150 mM KCl, pH 7.4 enriched with protease inhibitors (Roche Complete), lysozyme (0.8 mg ml^−1^ final), DNase I (4.2 µg ml^−1^ final), pefabloc (0.5 mg ml^−1^ final), benzamidine (5.3 mM final) and PMSF (0.6 mM final). The extract was incubated on ice for 10 min, sonicated, then centrifuged at 20,000*g* four times until a clear solution was obtained.

For human cell extract, Expi293F suspension cells (Thermo Fisher Scientific) were grown to a density of 2.5 × 10^6^ ml^−1^ in Freestyle serum and protein-free culture medium (Thermo Fisher Scientific) in a shaking incubator at 37 °C with 5% CO_2_. A total of 1.25 × 10^9^ cells was collected by centrifugation at 2,000*g* for 20 min and the culture medium was aspirated. The cell pellet was added to liquid nitrogen and homogenized to a powder under cryogenic conditions using pestle and mortar with 30 mg (1/2 tablet) of cOmplete EDTA-free protease inhibitor cocktail (Roche). Powdered lysate was stored at −80 °C, then thawed at 0 °C. The lysate was sonicated 10 times with 30 s cycles at 4 °C, then clarified by two rounds of centrifugation at 4 °C for 10 min at 1,000*g*, with the supernatant transferred to a new tube on ice each time and gently triturated.

Cytostatic-factor (CSF)-arrested *Xenopus laevis* egg extracts were prepared as previously described^63,64^, with the exception that cytochalasin D was replaced with cytochalasin B. In brief, unfertilized eggs were collected, dejellied and crushed by centrifugation. After extraction of the cytoplasmic fraction, a 1/1,000 volume of a protease inhibitor mixture (leupeptin, pepstatin and chymostatin) as well as a 1/1,000 volume of cytochalasin B were added to the extract, giving final concentrations of 10 µg ml^−1^ each. This CSF-arrested extract was stored on ice. To check the state of the CSF-arrested extract, a test reaction was prepared by addition of a small volume of frog sperm to 25 µl of the extract (to a final concentration of approximately 200 sperm per µl). After incubation at 18 °C for 30 min, arrest in meiosis II was confirmed by visualization of half-spindles using polarized-light microscopy as described previously^65^.

Interphase-arrested *X. laevis* egg extracts were prepared as previously described^66^, with minor modifications. In brief, unfertilized eggs were dejellied and then activated by incubation for 2–4 min in 0.2× Marc’s Modified Ringers (MMR) buffer supplemented with 0.5 µg ml^−1^ calcium-ionophore (A23817, Sigma-Aldrich). Activated eggs were then washed and crushed by centrifugation. As for the CSF-arrested extract, protease inhibitors and cytochalasin B were added to the cytoplasmic fraction to 10 µg ml^−1^ each. This cycling extract was stored on ice. To arrest in interphase, cycloheximide was added to a final concentration of 200 µg ml^−1^. After a 20 min incubation on ice, this interphase-arrested extract was subsequently stored on ice and used for osmometry. When an extract preparation yielded significantly more fresh extract than could be used that day, the excess was spin-filtered, frozen slowly (approximately 1 °C min^−1^) and stored at −80 °C.

### Yeast

*S. cerevisiae* stably expressing PLCδ-2xPH-GFP in the WT or *mss4*^*ts*^ in the *mss4-*null background (noted simply *mss4*^*ts*^ in the following detailed genotype below), were generated for us by C. Godlee and M. Kaksonen. The original plasmids expressing 2×PH-GFP and *mss4*^*ts*^ were a gift from the laboratory of S. Emr^17^. Yeast cells were grown overnight in liquid medium lacking Ura and Trp supplemented with 2% glucose. Cells were subsequently diluted to an optical density at 600 nm (OD_600_) of 0.1 and allowed to recover to an OD_600_ of 0.4. The yeast was then allowed to lightly adhere for 15 min to glass coverslips coated with concanavalin A (50 μg ml^−1^ in PBS, 30 min) before imaging. Imaging was performed in liquid medium lacking Ura and Trp supplemented with 2% glucose in the spinning-disk set-up described below. For hypoosmotic shocks, 50 μl of yeast medium was diluted with 950 μl of deionized water, giving a hypoosmotic shock of 405 mOsm l^−1^ to 23 mOsm l^−1^. Note that the frame acquired immediately after hypoosmotic shock was removed, due to a transient change in membrane signal, presumably due to the change in ionic gradient across the membrane. The temperature was maintained at 32 °C (hypoosmotic shock experiments; Figs. 1d–e and Extended Data Fig. 2d–f) or 39 °C (heat-shock experiments; Extended Data Fig. 2a–c) using a heated stage (TOKAI HIT INUB-ZILCS-F1).

*S. pombe* strains expressing the microtubule marker alpha tubulin (Atb2) fused to mCherry and the spindle pole body (SPB) marker Sid4 fused to GFP in the *cut7-24* background were maintained at 30 °C on yeast-extract-supplemented (YES) plates, and maintained every third day. *Cut7-24* induces monopolar spindle formation at the restrictive temperature^18,67^. For live-cell imaging, cells were transferred into liquid YES medium and imaged the next day during exponential growth. All *S. pombe* live-cell imaging was performed in eight-well μ-slides (Ibidi, IB-80807), preincubated over night with lectin (soybean, L1395, Merck) in the spinning-disk instrument described below, equipped with a heating chamber to maintain precise temperature (35 °C or 37 °C). Cells were incubated on the microscope stage for 30 min to ensure thermal equilibrium before the images were acquired. Stacks of seven planes (Δ*z* = 1 μm) were acquired for each channel with 100 ms exposure time for 488 nm and 200 ms for the 561 nm laser. For each time-lapse video, an image was taken every 5 min for 180 min. Cells treated with deuterium oxide (D_2_O) were grown in 50% 2× YES + 50% D_2_O over night before imaging. Exposure to hypoosmotic condition (5% YES + 95% H_2_O) was performed directly in the imaging dish at the microscope, with imaging starting directly after medium exchange.

Detailed genotypes were as follows: WT: MATa, his3Δ200, leu2–3, 112, ura3–52, lys2–801, pRS426-PLCdelta-2xPH-GFP, and *mss4*^*ts*^: MATa, his3Δ200, leu2–3, 112, ura3–52, lys2–801, mss4::hphNT1, YCplac111-mss4-2ts (CEN LEU2), pRS426-PLCdelta-2xPH-GFP (Fig. 1c–e); *mss4*^*ts*^: MATa, his3Δ200, leu2–3, 112, ura3–52, lys2–801, mss4::hphNT1, YCplac111-mss4-2ts (CEN LEU2), pRS426-PLCdelta-2xPH-GFP (Extended Data Fig. 2a–c); WT: MATa, his3Δ200, leu2–3, 112, ura3–52, lys2–801, pRS426-PLCdelta-2xPH-GFP, and *mss4*^*ts*^: MATa, his3Δ200, leu2–3, 112, ura3–52, lys2–801, mss4::hphNT1, YCplac111-mss4-2ts (CEN LEU2), pRS426-PLCdelta-2xPH-GFP (Extended Data Fig. 2d–f); and *cut7-24*: h+ , cut7–24, Sid4-GFP:Leu2+ , mCherry-Atb2:Hph (Extended Data Fig. 2g–i).

### Mouse primary fibroblasts and chondrocytes

All animal work was licensed by the Home Office under the Animals (Scientific Procedures) Act 1986, with Local Ethical Review by the Medical Research Council and University of Manchester. Primary lung fibroblasts were isolated as described previously^68^. Primary chondrocytes were isolated from 5-day-old mice using a previously described protocol^69^. In brief, 5-day-old C57Bl/6 PER2::Luc mice were euthanized by decapitation. The knee, hip and shoulder joints were dissected, and any soft tissue removed. Joint cartilage was subjected to pre-digestion with collagenase D 3 mg ml^−1^ in DMEM twice for 30 min at 37 °C with intermittent vortexing to remove soft-tissue leftovers. Subsequently, the cartilage was diced using a scalpel and digested overnight at 37 °C. Cells were dispersed by pipetting and passed through a 70 µM cell strainer. The cell suspension was then centrifuged and the pellet was resuspended in DMEM/F12 with 10% FBS and plated in T75 flasks. Cells were passaged only once before performing experiments.

For calcium imaging, primary chondrocytes were plated onto four-chamber 35-mm glass-bottomed dishes (Greiner Bio-One). A total of 0.5 µl of 1 mM Fluo-4 AM calcium dye (Thermo Fisher Scientific) was added to each chamber containing cells cultured in 500 µl DMEM/F12 medium and incubated for 20 min. Image acquisition was performed by confocal microscopy (see below). Hyperosmotic shock was induced by addition of sorbitol to the culture medium and hypoosmotic by addition of distilled water. Temperature changes were achieved by adding ice cold medium to the cell culture chamber at a mass calculated according to the following formula:

$$t=({m}_{1}{c}_{1}{t}_{1}+{m}_{2}{c}_{2}{t}_{2})/({m}_{1}{c}_{1}+{m}_{2}{c}_{2})$$

where *t* is the final temperature (°C), *c*_1,...,*n*_ is the specific heat of substances (kJ kg^−1^ °C) and *t*_1,...*n*_ is the temperatures of the substances (°C).

The correct temperature was confirmed using a handheld infrared thermometer.

### Viability assays

For viability experiments, Raji cells were seeded at 3 × 10^5^ ml^−1^ then collected and resuspended 24 h later in challenge RPMI. HFFs were seeded in six-well plates and confluent monolayers were placed in challenge DMEM. For hypothermic treatment, cells were transferred to precooled challenge RPMI or DMEM medium made by varying the NaCl concentration to achieve a final osmolality of between 150 and 450 mOsm l^−1^, placed into a precooled metal block and maintained at 4 °C or on ice for 24 h. For hyperthermic treatment cells were transferred to preheated challenge RPMI or DMEM made using 0–50% D_2_O, placed into a preheated metal block and incubated for 30 min at 47 °C (Raji suspension) or 45 min at 50 °C (adherent HFFs) before transferring to 0% D_2_O control medium and culturing for a further 24 h at 37 °C before viability analysis. For both hypo- and hyperthermic experiments, control cells were incubated in parallel in a metal block at 37 °C.

Raji suspension cell viability was assessed by flow cytometry. In brief, cells were centrifuged, washed twice in PBS then consecutively stained with Calcein AM (eBioscience) and DRAQ7 (Biostatus) according to manufacturer’s instructions before flow cytometry analysis (Fortessa instrument followed by analysis using FlowJo v.10.6). The viability was evaluated for 10,000 suspension cells per replicate. For HFF adherent cell viability, cells were similarly stained and the monolayer viability was assessed by imaging stained cells using an Evos FL Auto2 microscope then measuring live cells using Fiji^70^.

### Kinase assay

Full-length human WNK1 (residues 1–2,382) was expressed in Expi293 cells and purified as we previously reported^71^. GCN2 was expressed in Sf9 cells and purified as previously reported^72^. Kinase assays were performed with 50 ng protein and 0.5 µM kinase in assay buffer (50 mM HEPES, 100 mM KOAc, 2 mM MgAc_2_, 0.1 mM ATP, 0.2 mCi ml^−1^ γ-32P-ATP, 1× Phos-STOP, pH 7.5) with the indicated concentrations of PEG-20kDa (Sigma-Aldrich, 81300-1KG). Reactions were incubated at 32 °C for 30 min and then quenched with sample buffer and boiled. The samples were then diluted sevenfold before electrophoresis due to the high concentrations of PEG. Incorporation of γ-^32^P was analysed by exposing the gel to a phosphor screen.

### Actin polymerization assays

ADP-actin preparation was adapted from ref. ^48^. In brief, purified actin (Cytoskeleton) was resuspended according to the manufacturer’s instructions. Resuspended Ca^2+^-ATP-actin was then diluted to 50 µM in G-buffer (5 mM Tris-HCl pH 8.0, 0.2 mM CaCl_2_, 0.2 mM ATP) and incubated with 80 µM MgCl_2_ and 200 µM EGTA for 3 min on ice to exchange into Mg^2+^-ATP-actin. To ensure depletion of all of the remaining ATP in solution, 20 U ml^−1^ of hexokinase (H6380, Sigma-Aldrich), 0.2 mM ADP and 1 mM glucose were added to the solution and incubated for 3 h on ice. Finally, the solution was centrifuged at 100,000*g* for 1 h at 4 °C to remove actin filaments, and the supernatant containing Mg^2+^-ADP-actin was used for the rest of the experiments. The actin concentration was measured using the BCA assay (Pierce).

Actin polymerization was induced by preparing 50 µl solution of 0.7 µM Mg^2+^-ADP-actin in F-buffer (50 mM KCl, 1 mM MgCl_2_, 1 mM EGTA, 1 mM DTT, 10 mM imidazole pH 7.0) or G-buffer (5 mM Tris-HCl pH 8.0, 0.2 mM CaCl_2_, 1 mM DTT) supplemented with either 0.2 mM ADP or 1 mM ATP depending on the condition tested, and 100 mg ml^−1^ PEG-35kDa or 100 mg ml^−1^ dextran-35kDa. Note that 0.7 µM is above the critical concentration (*C*_c_) for ATP-actin, but below that of ADP-actin^73^. The solutions were allowed to polymerize for 1 h at room temperature and then centrifuged at 100,000*g* for 1 h at 4 °C. From each condition, 40 µl of the top of the supernatant was removed, saved and labelled as ‘supernatant’. The remaining 10 µl were discarded and the pellet was carefully washed three times with 3 × 50 µl of polymerization buffer. Finally, the pellet was resuspended in 50 µl of polymerization buffer, and 40 µl was saved and labelled as ‘pellet’. The samples were then analysed by SDS–PAGE on NuPAGE 4–12% Bis-Tris gels (Life Technologies) according to the manufacturer’s instructions. Instant Blue (Sigma-Aldrich) was used for total protein staining of gels (Extended Data Fig. 10i). Note that, to avoid artefacts during SDS–PAGE due to the high PEG-35kDa/dextran-35kDa concentrations, all samples were diluted two times before loading in the gel. As a control, we performed the same experiment with BSA in F-buffer to verify that proteins of similar size to monomeric actin do not pellet in the presence of dextran or PEG (Extended Data Fig. 10i (bottom lane)).

### Structural analysis

To estimate the water liberated by assembly of ADP-actin (Extended Data Fig. 10h), we used the surface area of actin as a proxy. The solvent-accessible surface area was measured using Pyrosetta^74^ with a probe radius of 2 Å. An existing Cryo-EM structure of F-actin was used (Protein Data Bank (PDB): 5ONV), with the solvent-accessible surface area (SASA) measured in the monomeric and assembled state. This analysis was repeated for other available actin structures (PDB: 4A7N, 7BT7, 8A2Z), and the SASA averaged and compared between the two states.

### GFP–FUS phosphorylation analysis

To analyse the specific effects of global modulations of the phospho-proteome on FusLC–GFP (Extended Data Fig. 8k), SH-SY5Y cells were transiently transfected with FusLC–GFP. Then, 24 h after transfection, cells were treated with Calyculin A (3 nM) for 15 min, or Staurosporine (10 µM) for 50 min at 37 °C under 5% CO_2_ (one confluent 10 cm dish per condition, per replicate). Cells were then washed twice with ice-cold PBS, scraped and collected in Falcon tubes. After centrifugation at 1,800 rpm for 5 min 4 °C, the pelleted cells were lysed for 20 min in lysis buffer (40 mM HEPES, 150 mM KOAc, 2.5 mM Mg(OAc)_2_, 1.0% Triton X-100, 0.1% SDS, pH 7.4) supplemented with protease inhibitors (Complete, Roche), phosphatase inhibitors (PhosSTOP, Roche) and DNase (D4513, Sigma-Aldrich). Lysates were then sonicated using a Bioruptor sonicator (Diagenode) at 4 °C for 3 cycles 30 s on/30 s off and cleared at 20,000*g* for 10 min in preparation for FusLC–GFP immunoprecipitation. Then, 25 µl of GFP-Trap Resin (gta-20, Chromotek) slurry was aliquoted and pre-equilibrated by washing in the lysis buffer three times. After equilibration, the lysates were loaded onto the beads and incubated for 2 h at 4 °C with rotation. The beads were then washed three times in wash buffer (20 mM HEPES, 150 mM KOAc, 2.5 mM Mg(OAc)_2_, 0.5% Triton X-100, pH 7.4, supplemented with protease and phosphatase inhibitors). The beads were then resuspended in 2× NuPage LDS sample buffer (Thermo Fisher Scientific), 5 mM DTT and boiled at 95 °C for 5 min. The samples were then analysed on regular, NuPage 4–12% Bis-Tris gels (NP0321BOX, invitrogen) or SuperSep Phos-tag 50 μmol l^−1^, 7.5% gels (192-18001, FUJIFILM) according to the manufacturer’s protocol. For immunoblotting, gels were briefly washed in Millipore water and protein transfer onto nitrocellulose membranes was performed using the dry transfer iBlot2 system (IB21001, Thermo Fisher Scientific) with a standard (P0, 7 min) protocol. The membranes were briefly washed in water, then blocked in 5% (w/w) non-fat dried milk (Marvel) TBS containing 0.1% Tween-20 (TBST) for 1 h at room temperature. The membranes were then incubated with primary anti-GFP antibodies (ab290, Abcam, 1:10 000 dilution) diluted in blocking buffer (5% milk, TBST), at 4 °C overnight. Subsequently, the membranes were washed three times for 10 min (in TBST) and then incubated with anti-rabbit HRP-conjugated secondary antibodies (A6154-1ML, Sigma-Aldrich, 1:10,000 dilution) diluted in blocking buffer for 1 h at room temperature. Excess secondary antibody was removed by washing three times for 10 min (in TBST). Chemiluminescence detection was performed by incubating the membranes with the Immobilon reagent (Millipore) and imaging using the ChemiDoc MP (Bio-Rad) system.

### Microscopy

#### Microscope hardware

Most imaging (except for that in Extended Data Figs. 3b,g and 4e) was performed using a custom TIRF/spinning-disk confocal microscope, comprising a Nikon Ti stand equipped with a perfect focus system and a fast-piezo stage (ASI). All data were collected with a ×100 PLAN Apo Lambda NA 1.45 or a ×60 PLAN Apo Lambda NA 1.4 objective. The confocal imaging arm consisted of a Yokogawa CSU-X1 spinning-disc head and a Photometrics 95B back-illuminated sCMOS camera, operated in pseudo global shutter mode (synchronized with the spinning-disc rotation). Camera binning was set to 1, and electronic gain was set to 1. Illumination was provided by 405 nm, 488 nm (150 mW OBIS LX), 561 nm (100 mW OBIS LS) or 630 nm (140 mW OBIS LX) coherent lasers mounted in a Cairn laser launch. Single band-pass filters (Chroma 525/50 for GFP, Chroma 595/50 for mCherry and Chroma ET655lp for A647/JF-646) or a quad bandpass filter (Chroma ZET405/488/561/640 m for Hoescht) were mounted within a fast Cairn Optospin filter wheel. To enable fast four-dimensional acquisition, an FPGA module (National Instrument sbRIO-9637 running custom codes) was used for hardware-based synchronization of the microscope. For *z*-axis acquisitions, the FPGA ensured that the piezo *z*-stage moved only during the readout period of the sCMOS camera. The whole system was controlled by Metamorph software, and the sample temperature was maintained using a custom heating enclosure (https://MicroscopeHeaters.com). For long-term videos (>2 h), open water bottles were added to the heating enclosure to increase humidity and decrease evaporation.

Alternatively, calcium imaging (Extended Data Fig. 3b) was performed using the Zeiss Exciter confocal microscope equipped with a 37 °C stage in humidified 5% CO_2_. Imaging was performed using a 488 nm excitation wavelength and 520 nm band-pass filter for emission and Fluar 40× NA 1.3 (oil-immersion) lens. Image capture was performed using the Zeiss software Aim v.4.2 with the Autofocus macro^75^. For adherent cell viability measurements, a Evos FL Auto2 microscope was used (Extended Data Figs. 3g and 4e).

#### In vitro condensation assays

To image condensation, we assembled home-made sealed chambers by sandwiching a 0.5-mm-thick PDMS insert between two glass coverslips. Inserts, with a 5-mm-diameter hole, were cut out of PDMS sheets (Silex Silicone) with a Graphtec CE6000 cutting plotter. Both glass surfaces were passivated with PLL-PEG (SUSOS, 0.5 mg ml^−1^ in 20 mM Tris, pH 7.4, 1 h, at room temperature) to prevent protein adsorption to the glass surface. Solutions were added to the well and the second coverslip was added to prevent the development of an air–water interface. Images were acquired within 2 min of protein addition, with protein added at the indicated concentration. PEG solutions were made up in 20 mM Tris + 150 mM KCl.

#### Imaging condensation in cells under various challenges

SH-SY5Y (Fig. 3a,c–e and Extended Data Fig. 8a,b,i,j) or U2OS (Fig. 3b and Extended Data Fig. 8c,d,g,h) cells were transiently transfected with plasmids expressing FusLC–GFP or TIA1-GFP 24 h before imaging. Cells were spread for 1 h on glass-bottom imaging dishes (fluorodishes, World precision instruments) coated with fibronectin (50 μg ml^−1^ in PBS, 1 h) before imaging. SH-SY5Y cells were imaged at 37 °C in Leibowitz’s L15 medium supplemented with 20% FBS and 20 mM HEPES pH 7.6. U2OS cells were imaged in DMEM high glucose, 10% Hyclone II and penicillin–streptomycin supplemented with 25 mM HEPES at 37 °C. In both cell lines, the extracellular osmolarity was altered by the addition of distilled water or sucrose. Cells were imaged before and after osmotic challenge by confocal *z*-stack imaging, and a maximum-intensity projection was computed. Then, the granulosity index, a measure of the condensation of the GFP signal, was measured in the automatically or manually segmented nucleus (see below). For each cell, data were normalized to the value of the granulosity index cell before osmotic challenge. We consistently incubated cells with fresh, prewarmed medium for pre-osmotic control measurements to avoid accumulation of osmolytes and cell byproducts in the medium over time that might otherwise change extracellular osmolarity and therefore alter the response of the cell. Unless stated otherwise, hyperosmotic shocks were performed by adding an identical volume of warm medium with twice the desired sucrose concentration onto the cells.

For imaging of nucleolar condensation in response to a hypoosmotic challenge (Extended Data Fig. 8e,f), untransfected SH-SY5Y cells were stained with Nucleolar-ID (Enzo) for 15 min according to the manufacturer’s instructions, before imaging over time after hypoosmotic challenge (50% distilled water in Leibowitz’s L15 medium supplemented with 20% FBS and 20 mM HEPES pH 7.6).

For imaging of FusLC–GFP condensation in response to global changes in protein phosphorylation (Extended Data Fig. 8i,j), SH-SY5Y cells transfected with FusLC–GFP plasmid were spread onto fibronectin-coated glass-bottom imaging dishes as described above and imaged over time in Leibowitz’s L15 medium supplemented with 20% FBS and 20 mM HEPES pH 7.6, after addition of Calyculin A (3 nM) or 10 µM Staurosporine.

For imaging of FusLC–GFP condensation in energy-depleted cells (Extended Data Fig. 8g,h), we adapted a previously established protocol for energy depletion^76^. Specifically, U2OS cells were transfected with FusLC–GFP for 24 h and plated onto fibronectin-coated glass-bottom imaging dishes (ibidi 8-well dishes) in full growth medium for 2 h at 37 °C. The medium was then exchanged for prewarmed glucose/sodium pyruvate/glutamine-free DMEM (Thermo Fisher Scientific, A1443001) with the addition of 5% FBS, 1× GlutaMAX (Thermo Fisher Scientific), 10 mM 2-deoxy-d-glucose (Sigma-Aldrich, D6134), 10 mM sodium azide (Sigma-Aldrich, 08591) and 10 mM HEPES. Incubation for 30 min in this medium at 37 °C induced the characteristic absence of lamellipodia/ruffles (Extended Data Fig. 8g), as well as characteristic spike protrusions as previously reported^77^ (Extended Data Fig. 8g (blue arrows)), demonstrating the efficacy of the energy-depletion treatment. As non-energy-depleted controls, cells were incubated in glucose/sodium pyruvate/glutamine-free DMEM (Thermo Fisher Scientific, A1443001) with the addition of 5% FBS, 1× GlutaMAX (Thermo Fisher Scientific), 10 mM d-glucose, 10 mM HEPES and 1× sodium pyruvate (Thermo Fisher Scientific) for 30 min at 37 °C. After acquisition of *z*-stacks under these isosmotic conditions, a hyperosmotic shock was induced by addition of one-tenth of total volume of a 584 mM concentrated sucrose solution (20 µl in 200 µl) and *z*-stacks of the same cells were reacquired.

For imaging of FusLC–GFP condensation in response to hyperosmotic shock in the presence of D_2_O (Extended Data Fig. 4g,h), U2OS cells were transiently transfected with FusLC–GFP plasmid 24 h before imaging. Confocal *z*-stacks of cells were acquired in Leibowitz’s L15 medium supplemented with 20% FBS and 20 mM HEPES pH 7.6, before the extracellular osmolarity was altered by the addition of sucrose (+20 mOsm l^−1^) in the presence or absence of 50% D_2_O. The granulosity index was then measured in the nucleus and, for each cell, data were normalized to the value of the granulosity index cell before osmotic challenge.

For imaging of the long-term decrease in FusLC–GFP condensation in response to sustained hyperosmotic shocks (Extended Data Fig. 11a), U2OS cells were transiently transfected with FusLC–GFP plasmid 24 h before imaging. Cells were then plated onto fibronectin-coated, glass-bottom 35 mm imaging dishes as described above for 2 h at 37 °C under 5%CO_2_ in DMEM high glucose supplemented with 10% Hyclone II and penicillin–streptomycin. Cells were then washed in DMEM high glucose supplemented with 10% Hyclone II, penicillin–streptomycin and 20 mM HEPES and 1 ml of this medium was added to the dish. Hyperosmotic shock was induced by adding 1 ml of DMEM high glucose supplemented with 10% Hyclone II, penicillin–streptomycin, 20 mM HEPES 150 mM sucrose, leading to a +75 mOsm osmotic shock (verified by osmometry). The dish was then sealed using a 40 mm coverslip and vacuum grease, and confocal imaging was initiated. Confocal *z*-stacks of cells were acquired every 30 min with a large *z*-spacing (20 planes, Δ*z* = 0.75 µm) and at the minimum laser power to minimize photobleaching. At these long timescales, only qualitative analysis of condensation is possible, as multiple other variables come into play that (may) affect condensation (cell migration, cell-cycle state), as well as further expression of the transgene which will change the global fluorescence of the cell, which will bias our measurements. But, when recovery is observed, this occurs over hour timescales, which is consistent with established mechanisms of osmoregulation and the behaviour of other IDR proteins^43,50,78,79^.

For fast imaging of short-term increase of FusLC–GFP condensation in response to hyperosmotic shock (Extended Data Fig. 6d,e and Supplementary Video 2), U2OS cells transfected with FusLC–GFP for 24 h were plated onto fibronectin-coated glass-bottom imaging dishes (ibidi 8-well dishes) in full growth medium (DMEM high glucose, 10% Hyclone II, sodium pyruvate, penicillin–streptomycin) for 2 h at 37 °C. Cells were washed once in full growth medium supplemented with 25 mM HEPES and left in 250 µl of this medium. Fast confocal acquisition was then started (single confocal plane, perfect focus enabled, camera operating in streaming mode, effective time between frames 140 ms), and 125 µl of prewarmed full growth medium enriched with 25 mM HEPES and 200 mM sucrose was added after 100 frames. This results in a +67 mOsm l^−1^ shock.

#### Microfluidic control of temperature and osmolarity while imaging condensation

For imaging of cells undergoing both rapid temperature change in various osmotic conditions (Fig. 3c,d), we used the established Cherry Temp system microfluidics system (Cherry Biotech). With this system, it takes about 4 s for a temperature change from 37 °C to 20 °C at the sample^80^. In brief, cells were spread in custom-made chambers made of a coverslip coated for 1 h with cell-adhesive fibronectin (Sigma-Aldrich, 50 μg ml^−1^ in PBS), a PDMS spacer (0.5 mm thick) and a Thermalization chip (Cherry Biotech) at the top. Cells were imaged at different temperatures, in Leibowitz’s L15 medium supplemented with 20% FBS and 20 mM HEPES pH 7.6, osmolarity adjusted with sucrose or distilled water. The same cells were imaged within each temperature series, but different cells were imaged at the different extracellular osmolarities.

For simultaneous imaging of FusLC–GFP condensation within single cells in response to temperature and external osmolarity changes (Fig. 3e and Supplementary Video 1), we used dual-layer microfluidics devices (Thermaflow chips, Cherry Biotech). This combines fast temperature changes using the Cherry Temp system (see above), and fast medium changing using in an independent microfluidics loop (Elvesys). This cell medium microfluidics loop is composed of an OB1-positive pressure regulator (Elvesys), a ten-input MUX distributor valve (Elvesys) to select the different medium to add to cells, a bubble trap and a flow sensor positioned just at the entrance of the flow chamber. Flow was maintained at a constant low value of 114 µl min^−1^ to avoid shear stress to the cells. The glass bottom of these microfluidics chips was coated with fibronectin (50 μg ml^−1^ in PBS, 1 h), then SH-SY5Y cells transfected with FusLC–GFP were allowed to adhere in the flow chamber, before imaging using SDCM (21 *z*-planes, Δ*z* = 0.5 µm) in Leibowitz’s L15 medium supplemented with 20% FBS and 20 mM HEPES pH 7.4. The temperature was then independently controlled using the Cherry Niotech top layer, while the external osmolarity was controlled by changing the medium going into the chamber by changing the input in the bottom layer for a 50:50 (vol:vol) dilution of medium in distilled water (resulting in a 325 to 162.5 mOsm l^−1^ hypoosmotic shock). Cells were kept in focus using hardware autofocus (perfect focus, Nikon).

### Image processing

Images were processed using Fiji^70^ and MATLAB 2020b (MathWorks) using custom codes that are available on request. For visualization purposes, the PopRed lookup table from the J. Manton collection (https://github.com/jdmanton/ImageJ_LUTs) was applied to most monochrome images after the dynamic range was adjusted between minimum and maximum grey values of each image (note that the dynamic range was not kept identical between images when presenting different conditions). Figures were assembled in Adobe Illustrator 2021. Videos were edited in Adobe Premiere 2021.

Where appropriate, spatial drift during acquisition of videos was corrected using a custom GPU-accelerated registration code based on cross-correlation between successive frames. For representation purposes, a wavelet ‘à trous’ denoising filter was applied to Extended Data Fig. 8c (custom GPU-accelerated MATLAB port of a code originally developed by F. Cordeliere for the Improve Kymo ImageJ plugin^81^). The raw images were averaged with the filtered video. Both codes are available at our GitHub page (https://github.com/deriverylab), as well as the codes for quantification of protein condensation and nuclear segmentation described below.

#### Quantification of condensation in vitro

Our pipeline to objectively assess the degree of condensation in microscopy images for in vitro assays (Fig. 4 and Extended Data Fig. 9) is presented in Extended Data Fig. 9a,b.

In brief, we computed the fast Fourier transform (FFT) of the source images (all 600 × 600 pixels, resulting in a 1,024 × 1,024 Fourier-transformed image), and the fraction of the power spectrum found in rings of increasing diameter (3 px increment in Fourier space) was then measured and plotted onto a log scale (Extended Data Fig. 9a). As can be seen in Extended Data Fig. 9a, as the fraction of condensed signal increases in the source image, a larger fraction of the power spectrum is found in rings of larger diameter (in other words, high spatial frequency components increasingly appear in the image). This was highly consistent for various samples of a given condition (Extended Data Fig. 9a (bottom left)). As the first two rings contained mostly the image background (low spatial frequencies), we set a threshold of 6 px in the Fourier space and defined the condensation ratio as the fraction of the total power spectrum in the high-frequency portion of the spectrum (that is, >6 px in Fourier space). This 6 px threshold was efficient at separating the condensed signal from the non-condensed signal and background (Extended Data Fig. 9b,c).

#### Quantification of condensation in live cells

Our pipeline to objectively assess the degree of condensation in microscopy images for live-cell assays is presented in Extended Data Fig. 6a. As the condensation in cells had to be measured in specific regions of interests (ROIs), rather than the full image, we could not use the condensation ratio described above, as that calculation is done in the Fourier space, not the real space, in which ROIs can be made.

In brief, raw images were processed for homogenous background subtraction, then FFT was computed, then a high-pass filter was applied using a circular mask followed by inverse FFT. This mask was kept constant for all images (all source data were cropped to have the same size). We next computed the granulosity ratio as the ratio between the s.d. and the mean of the signal in the high-pass-filtered image. As the granulosity index is measured in the real space, which can be done in specific ROIs (for example, the nucleus). The automated nucleus segmentation using an ad hoc neural network that we developed for this report is described in a dedicated section below.

Importantly, we confirmed that both the condensation ratio (measured in the Fourier space) and the granulosity index (measured in the real space) are giving quantitatively similar results using input images from GFP condensation in vitro (Extended Data Fig. 6b,c; for the granulosity index, the whole image was used as the ROI).

#### Quantification of cortical PIP_2_ recruitment

For the quantification of the yeast thermosensitive mutant (Fig. 1d,e and Extended Data Fig. 2), semi-automated analysis was performed using custom scripts in Fiji. *z*-Stacks were acquired by SDCM at a stable temperature (Δ*z* = 0.5 μm, Δ*t* = 1 min). Intensity thresholds were applied manually to each cell independently, and the thresholded region was converted to a region of interest. These regions were smoothed and filled, and the peripheral-most 3 px were segmented out and used to measure membrane-bound GFP intensity. The region within this was used for the cytosolic intensity. The ratio of the median membrane-bound and cytosolic signal, after homogenous background subtraction, was then computed for each cell.

#### Neural network for nuclear segmentation

For automated nuclear segmentation, SH-SY5Y cells transiently expressing FusLC–GFP were stained with Hoechst (5 min, 13.6 μM) and imaged using SDCM. Maximum-intensity *z*-projections were then thresholded on the basis of the Hoechst signal to establish the initial nuclear ground truth. After initial training of the network on these 132 FusLC–GFP images, predictions were manually refined to increase the training dataset size to 598 images. This dataset was then used to retrain the network. Image augmentation was performed on the training dataset, by splitting the images into four overlapping tiles, flipping or rotating each tile (90°, 180° and 270°) and duplicating images and randomly resetting the contrast of these duplicate images.

The network architecture used is a residual convolutional U-net^82^ and is depicted in Extended Data Fig. 7a,b. It comprises a residual convolutional network^83^, with concatenation steps between down- and up-sampling layers of the network. Each layer of the network comprised two residual blocks, each of which comprise two 2D 3 × 3 convolutional layers with batch normalization and a ReLU activation function. 2 × 2 max pooling layers were used to reduce dimensionality of the network, before subsequent upsampling of the image with transposed 2D convolutional layers. A final 1 × 1 2D convolutional layer and sigmoid activation outputted a probability of each pixel being with a nucleus, and these probabilities were thresholded to output a binary segmentation. The dropout rate was set at 0.25 for the first and convolutional/residual block and the last deconvolutional/residual block, and 0.5 for all other blocks, and a batch size of 8 images was used. A binary cross entropy loss was used, with Adam optimizer and a learning rate of 1 × 10^−4^. The final training took approximately 2 days on two Nvidia Titan V GPUs. The final network was used for the automatic segmentation of all cellular condensation data imaged with a ×100 objective (Extended Data Fig. 7e). For Fig. 3d, which was imaged using a ×60 objective, and Extended Data Fig. 8a, nuclei were manually segmented using Fiji.

#### Calcium signalling

Fluorescence analysis was performed by measuring the intensity of the Fluo-4 probe on all cells in the field of view using the Zeiss software Aim v.4.2.

#### Monopolar spindle quantification

For quantification of monopolar spindle occurrence (Extended Data Fig. 2g–i), maximum projections of each stack were generated using Fiji. The number of monopolar and bipolar spindles was manually scored within the time frame of 60–180 min of each video (6–10 videos were acquired in each condition per experiment).

### Proteomics and phosphoproteomics

#### Culture conditions

Primary mouse lung fibroblasts were grown to confluence in 10 cm dishes in DMEM supplemented 10% Hyclone III and penicillin–streptomycin (100 U ml^−1^), and in duplicates transferred to the following conditions for two weeks: control (37 °C, 350 mOsm l^−1^ medium), hypoosmotic (37 °C, 450 mOsm l^−1^ medium), hyperosmotic (37 °C, 250 mOsm l^−1^ medium), low temperature (32 °C, 350 mOsm l^−1^ medium) and high temperature (40 °C, 350 mOsm l^−1^ medium), with the medium changed every 3–4 days. Medium osmolarity was adjusted with water or sucrose. Cells were washed twice in ice-cold PBS and then lysed at room temperature in 1 ml freshly prepared lysis buffer (8 M urea, 20 mM Tris, pH 8) for 20 min. After lysis, the dishes were scraped and the cell lysates were immediately flash-frozen in liquid nitrogen and then stored at −80 °C. All of the samples were simultaneously defrosted and sonicated for 2 min and the protein concentration was then measured using the BCA assay (Pierce).

#### MS analysis

##### Enzymatic digestion

Each sample (500 µg) was reduced with 5 mM DTT at 56 °C for 30 min and then alkylated with 10 mM iodoacetamide in the dark at room temperature for 30 min. They were then digested using MS-grade Lys-C (Promega) at a protein:Lys-C ratio of 100:1 (w/w) for 4 h at 25 °C. Next, the samples were diluted to 1.5 M urea using 20 mM HEPES (pH 8.5) and digested at 30 °C overnight with trypsin (Promega) at a ratio of 70:1 (w/w). Digestion was quenched by the addition of formic acid (FA) to a final concentration of 1%. Any precipitates were removed by centrifugation at 16,000*g* for 10 min. The supernatants were desalted using home-made C18 stage tips containing 3 M Empore extraction disks (Sigma-Aldrich) and 8 mg of Poros R3 resin (Thermo Fisher Scientific). Bound peptides were eluted with 30–80% acetonitrile (MeCN) in 0.5% formic acid and lyophilized.

##### TMT peptide labelling

The lyophilized peptides from each sample were resuspended in 100 µl of 2.5% MeCN and 250 mM triethylammonium bicarbonate. Then, 60 µl (1.2 mg) of each TMT10plex reagent (Thermo Fisher Scientific), reconstituted in anhydrous MeCN according to the manufacturer’s instructions, was added. Peptides from each timepoint were labelled with a distinct TMT tag for 60 min at room temperature. The labelling reaction was quenched by incubation with 11 µl 5% hydroxylamine for 30 min. The set of 10 labelled peptides (5 conditions, duplicates) were combined into a single sample and partially dried to remove MeCN in a SpeedVac (Savant). The sample was then acidified and centrifuged at 16,000*g* for 10 min. The supernatant was desalted using Sep-Pak Plus Short tC18 cartridges (Waters). Bound peptides were eluted with 60% acetonitrile in 0.5% acetic acid and lyophilized.

##### Titanium dioxide enrichment of phosphopeptides

Phosphopeptides were enriched using TiO_2_ titansphere-chromatography (GL Science). Lyophilized peptides were resolubilized in 50% MeCN containing 2 M lactic acid (loading buffer) and incubated with TiO_2_ beads (1:4, peptides:TiO_2_(w/w)) that were prewashed with loading buffer. After 1 h, the TiO_2_ beads were centrifuged at 10,000*g* for 2 min, the supernatant was added to fresh TiO_2_ beads for a second round of enrichment and the procedure was repeated for a third time. After incubation, the TiO_2_ beads with enriched phosphopeptides were loaded onto C8 stage tips (3 M Empore) and washed twice with loading buffer and once with 50% MeCN, 0.1%TFA. Phosphopeptides were eluted sequentially with 0.4 M ammonia solution, 30% MeCN in 0.4 M ammonia solution and 50% MeCN in 0.1% TFA. The eluates were acidified with formic acid, and partially dried down using a SpeedVac (Savant). The samples were then desalted using C18 Stage tips (3 M Empore) and lyophilized.

##### Basic pH reverse-phase HPLC fractionation

The TMT-labelled peptides and phosphopeptides were processed for off-line high performance liquid chromatography (HPLC) fractionation, using an XBridge BEH130 C18, 5 μm, 2.1 mm x 150 mm column with an XBridge BEH C18 5 μm Van Guard cartridge (Waters), connected to an Ultimate 3000 Nano/Capillary LC System (Dionex). Peptide mixtures were resolubilized in solvent A (5% MeCN, 95% 10 mM ammonium bicarbonate, pH 8) and separated with a gradient of 1–90% solvent B (90% MeCN, 10% 10 mM ammonium bicarbonate, pH 8) over 60 min at a flow rate of 250 μl min^−1^. Eluted peptides were collected at 1 min per fraction, combined into 18 fractions for proteomics experiments and 14 fractions for phosphoproteomics and lyophilized. For proteomic experiments only, the fractions were desalted using C18 stage tips and partially dried down by vacuum centrifugation before analysis using liquid chromatography (LC)–MS/MS.

##### LC–MS/MS analysis

The fractionated peptides were analysed by LC–MS/MS using the fully automated Ultimate 3000 RSLC nano System (Thermo Fisher Scientific) fitted with a 100 μm × 2 cm PepMap100 C18 nano trap column and a 75 μm × 25 cm nanoEase M/Z HSS C18 T3 column (Waters). The samples were separated using a binary gradient consisting of buffer A (2% MeCN, 0.1% formic acid) and buffer B (80% MeCN, 0.1% formic acid), and eluted at 300 nl min^−1^ with an acetonitrile gradient. The outlet of the nano column was directly interfaced through a nanospray ion source to the Q Exactive Plus mass spectrometer (Thermo Fisher Scientific). The mass spectrometer was operated in standard data-dependent mode, performing a MS full-scan in the *m*/*z* range of 380–1,600, with a resolution of 70,000. This was followed by MS2 acquisitions of the 15 most intense ions at a resolution of 35,000 and normalized collision energy of 33%. MS target values of 3 × 10^6^ and MS2 target values of 1 × 10^5^ were used. The isolation window of precursor ion was set at 0.7 Da and sequenced peptides were excluded for 40 s.

##### Spectral processing and peptide and protein identification

The acquired raw files from LC–MS/MS were processed using MaxQuant^84^ with the integrated Andromeda search engine (v.1.6.6.0). MS/MS spectra were quantified with reporter-ion MS2 from TMT10plex experiments and searched against the *Mus musculus* UniProt Fasta database (March 2019). Carbamidomethylation of cysteines was set as fixed modification, whereas methionine oxidation, N-terminal acetylation and phosphorylation (STY) (for the phosphoproteomics group only) were set as variable modifications. Protein quantification requirements were set at 1 unique and razor peptide. In the identification tab, second peptides and match between runs were not selected. The other parameters in MaxQuant were set to the default values.

The MaxQuant output file was then processed using Perseus (v1.6.6.0). Reporter-ion intensities for the protein group table were uploaded to Perseus. The data were filtered: identifications from the reverse database were removed, only identified by site, potential contaminants were removed. Then, all columns with an intensity of less than or equal to zero were converted to ‘not a number’ (NAN) and exported. The MaxQuant output file with phospho (STY) sites table was also processed using Perseus software (v.1.6.6.0). The data were filtered: identifications from the reverse database, potential contaminants were removed and we only considered phosphopeptides with localization probability ≥0.75. Then all columns with an intensity of less than or equal to zero were converted to NAN and exported.

#### MS data analysis

Data processing was performed in R v.3.6.1 using R Studio v.1.2. For proteomics data, reporter-ion intensities were normalized for input (equalizing total intensities) and log_2_-transformed. To determine proteins that change significantly with temperature or external osmolarity, linear model fitting with the empirical Bayes method was performed using the LIMMA package^85^, separately for temperature and osmolarity data, with predictors treated as continuous variables. A Benjamini–Hochberg-adjusted *P* value of 0.05 was used as the significance threshold. The GeneOverlap R package^86^ was used to aid determination of overlap significance between different protein groups. Gene Ontology enrichment was performed using the GOrilla online tool^87^ with two unranked gene lists (overlaps versus all detected proteins). A list of phase-separating proteins was taken from PhaSepDB (v.1)^88^, using the list of proteins annotated as phase-separating and/or belonging to different MLOs from high-throughput screens.

For phosphoproteomics data, reporter-ion intensities for each phosphosites were normalized to its respective protein abundance (obtained from original proteomics data). LIMMA analysis was performed as described above. D2P2 database^89^ was queried for all detected phosphoproteins to determine their predicted IDRs, as a consensus between the different predictor algorithms used in the database; the position of each phosphosite was used to determine whether or not it is predicted to be in an IDR. A proportion *z*-test was then used to determine whether phopshopeptides that change significantly with external osmolarity and/or temperature have the same or different level of IDR phosphorylation compared with the background. Prediction of kinases that might phosphorylate the detected phosphopeptides was performed by counting predicted motifs for a panel of 25 kinases present in the PHOSIDA database^90^.

### Statistics and reproducibility

Statistical analyses were performed using GraphPad Prism v.9.4.0 (673). Data are presented as mean ± s.e.m. unless otherwise stated. No randomization methods were used in this study. No blind experiments were conducted in this study. Normality of variables was verified using Kolmogorov–Smirnov tests. Homoscedasticity of variables was always verified when conducting parametric tests. Unless stated otherwise, no adjustments for multiple comparisons were performed (ANOVA tests were performed when comparing more than two samples, with adequate adjusted post hoc tests indicated in their respective figure legends). Unless stated otherwise, *n* values indicate independent biological replicates. The following statistical tests were two-sided: Figs. 1a,f,g, 2c, 3b,d and 4c,e,h and Extended Data Figs. 2i, 3d,f, 4b,f,h, 6c, 8a,b,d,h, 9g,i and 10f,l. Unless stated otherwise, *P* values are indicated by asterisks: *P ≤ 0.05, **P ≤ 0.01, ***P ≤ 0.001, ****P ≤ 0.0001; NS, *P* > 0.05.

Details on number of repeats when representative images/panels are shown are as follows: Fig. 1d: *n* = 30 (WT) and *n* = 16 (*mss4*^*ts*^) (quantified in Fig. 1e); Fig. 3a: *n* = 7 (GFP) and *n* = 12 (FusLC–GFP) (quantified in Extended Data Fig. 8a); Fig. 3e: *n* = 1 (quantified in Fig. 3d on *n* = 10–42 cells per condition); Fig. 4a: *n* = 10 per condition; Fig. 4b: *n* = 10 per condition (quantified in Fig. 4c); Fig. 4d: *n* = 10 per condition (quantified in Fig. 4e); Fig. 4g: *n* = 10 per condition (quantified in Fig. 4h); Extended Data Fig. 2b: *n *= 30 (32 °C) and *n* = 41 (39 °C) (quantified in Extended Data Fig. 2c); Extended Data Fig. 2e: *n* = 13 for each sample (quantified in Extended Data Fig. 2f); Extended Data Fig. 2h: respective sample size is indicated in the quantification in Extended Data Fig. 2i; Extended Data Fig. 3b: *n* = 20 (control), *n* = 20 (+100 mOsm l^−1^) and *n* = 18 (−17 °C) (Extended Data Fig. 3d); Extended Data Fig. 3g: *n* = 9 fields of view analysed per condition. Experiment representative of 3 biological replicates; Extended Data Fig. 4e: *n* = 9 fields of view analysed per condition. Experiment representative of 2 biological replicates; Extended Data Fig. 4g: *n* = 15 cells per condition (quantified in Extended Data Fig. 5h); Extended Data Fig. 6b: *n* = 10 (quantified in Extended Data Fig. 6c); Extended Data Fig. 6d: *n* = 1 (shown only for illustrative purpose); Extended Data Fig. 7e: *n* = 46 images in the validation set (best model accuracy = 0.996; loss, 0.065); Extended Data Fig. 8c: *n* = 9–23 (quantified in Extended Data Fig. 8d); Extended Data Fig. 8e: *n* = 8 (quantified in Extended Data Fig. 8f); Extended Data Fig. 8g: *n* = 10–11 (quantified in Extended Data Fig. 8h); Extended Data Fig. 8i: *n* = 9–12 (Staurosporine) and *n* = 7–9 (CalyculinA) (quantified in Extended Data Fig. 8j); Extended Data Fig. 8k: *n* = 2 (both independent biological repeats shown); Extended Data Fig. 9c: *n* = 10 images per condition; Extended Data Fig. 9d: *n* = 10 images per condition; Extended Data Fig. 9e: *n* = 10 images per condition (quantified in Fig. 4c); Extended Data Fig. 9f: *n* = 10 images per condition (quantified in Extended Data Fig. 10g), note that this is the same quantification as in Extended Data Fig. 6c (left panel); Extended Data Fig. 9h: *n* = 8 (GFP) and *n* = 10 (GFP + GBP) (quantified in Extended Data Fig. 9i); Extended Data Fig. 10i: *n* = 2; Extended Data Fig. 11a: dataset comprising *n* = 17 cells in 2 independent experiments; and Extended Data Fig. 11b: *n* = 4 (WNK1) and *n* = 2 (GCN2).

### Reporting summary

Further information on research design is available in the Nature Portfolio Reporting Summary linked to this article.

## Online content

Any methods, additional references, Nature Portfolio reporting summaries, source data, extended data, supplementary information, acknowledgements, peer review information; details of author contributions and competing interests; and statements of data and code availability are available at 10.1038/s41586-023-06626-z.

### Supplementary information

Supplementary Information Supplementary Discussion and references specific to the Supplementary Information.

Reporting Summary

Supplemental Fig. 1 Full gels for all western blot, Coomassie-stained and autoradiography gels presented in this study.

Supplementary Table 1 Proteome adaptation to temperature and external osmolarity challenges. This table was used to make the diagrams in Fig. 2b (see also Extended Data Fig. 6a–d). Quiescent primary fibroblasts were cultured in duplicate for 14 days in the indicated conditions, corresponding to adaptation to increased or decreased temperature/osmolarity and subjected to quantitative proteomics (TMT-MS/MS). The table shows the variation in abundance for the 7,634 proteins detected in all conditions. Also includes volcano plots of proteins that were upregulated (slope > 0) or downregulated (slope < 0) with increasing temperature or external osmolarity. Thresholds for slope (log_2_[FC], *x* axis) and non-adjusted *P* value (*y* axis) are for visualization purposes only; for the downstream analysis (determining which proteins change significantly in either direction), the threshold was set at a Benjamini–Hochberg-adjusted *P* value of 0.05.

Supplementary Table 2 Phosphoproteome adaptation to temperature and external osmolarity challenges. This table was used to make the diagram in Fig. 2d (see also Extended Data Fig. 6e–g). The same cell population used for Supplementary Table 1 also subjected to quantitative phosphoproteomics (TMT-MS/MS). The table shows the variation in abundance for the 14,530 phosphosites detected in all conditions. Also includes volcano plots of phosphoproteins that were upregulated (slope > 0) or downregulated (slope < 0) regulated with increasing external osmolarity or temperature. Thresholds for slope (log_2_[FC], *x* axis) and non-adjusted *P* value (*y* axis) are for visualization purposes only; for the downstream analysis (determining which phosphoproteins change significantly in either direction), the threshold was set at a Benjamini–Hochberg-adjusted *P* value of 0.05.

Supplementary Video 1 FusLC–GFP condensation in response to temperature and osmotic changes. SH-SY5Y transiently expressing FusLC–GFP were plated onto fibronectin-coated coverslips and imaged by SDCM (21 *z*-planes, Δ*z* = 0.5 µm), during fast changes of temperature (Δ*T* = 17 °C) or external osmolarity (Δ = −162.5 mOsm l^−1^) using dual-layer microfluidic chips (Methods). The images correspond to maximum-intensity *z*-projections. Scale bars, 10 µm (middle) and 1 µm (right).

Supplementary Video 2 Fast dynamics of FusLC–GFP condensation in response to hyperosmotic changes. Left, U2OS cells transiently expressing FusLC–GFP were plated onto fibronectin-coated coverslips and imaged by fast SDCM. After 100 frames (14 s), a +67 mOsm l^−1^ osmotic shock was induced (Methods). Images correspond to a single confocal plane. Scale bar, 10 µm. Right, dynamics of condensation automatically quantified using the granulosity index (Methods and Extended Data Figs. 7 and 8).

Supplementary Video 3 Narrated animation summarizing the paradigm proposed by this study, namely that macromolecular condensation buffers intracellular water potential.

### Source data

Source Data Fig. 1

Source Data Fig. 3

Source Data Fig. 4

Source Data Fig. 5

Source Data Extended Data Fig. 1

Source Data Extended Data Fig. 2

Source Data Extended Data Fig. 3

Source Data Extended Data Fig. 5

Source Data Extended Data Fig. 7

Source Data Extended Data Fig. 9

Source Data Extended Data Fig. 10

Source Data Extended Data Fig. 11

## Data Availability

The MS proteomics and phosphoproteomics data have been deposited at the ProteomeXchange Consortium through the PRIDE partner repository^91^ under dataset identifier PXD044481. Proteomics analysis was performed using the *Mus musculus* UniProt Fasta database from March 2019. The list of phase-separating proteins was taken from PhaSepDB v1. All other data supporting the findings of this study are available from the corresponding authors on reasonable request. Source data are provided with this paper.

## Code availability

Custom image-processing codes specific to this paper have been deposited at our GitHub page (https://github.com/deriverylab/granulosityindex and https://github.com/deriverylab/nuclearsegmentation). Similarly, our general image registration and wavelet filtering codes can also be found at GitHub (https://github.com/deriverylab/GPU_registration and https://github.com/deriverylab/GPU_wavelet_a_trous). All lookup tables applied to images in this paper come from the collection from J. Manton (https://github.com/jdmanton/ImageJ_LUTs).
