# Author Correction: Macromolecular condensation buffers intracellular water potential

**DOI:** 10.1038/s41586-024-07346-8

**Published:** 2024-04-08

**Authors:** Joseph L. Watson, Estere Seinkmane, Christine T. Styles, Andrei Mihut, Lara K. Krüger, Kerrie E. McNally, Vicente Jose Planelles-Herrero, Michal Dudek, Patrick M. McCall, Silvia Barbiero, Michael Vanden Oever, Sew Yeu Peak-Chew, Benjamin T. Porebski, Aiwei Zeng, Nina M. Rzechorzek, David C. S. Wong, Andrew D. Beale, Alessandra Stangherlin, Margot Riggi, Janet Iwasa, Jörg Morf, Christos Miliotis, Alina Guna, Alison J. Inglis, Jan Brugués, Rebecca M. Voorhees, Joseph E. Chambers, Qing-Jun Meng, John S. O’Neill, Rachel S. Edgar, Emmanuel Derivery

**Affiliations:** 1 MRC Laboratory of Molecular Biology, Cambridge, UK; 2 Department of Infectious Disease, Imperial College London, London, UK; 3 Wellcome Centre for Cell Matrix Research, University of Manchester, Manchester, UK; 4 Cluster of Excellence Physics of Life, TU Dresden, Dresden, Germany; 5 Max Planck Institute of Molecular Cell Biology and Genetics, Dresden, Germany; 6 Max Planck Institute for the Physics of Complex Systems, Dresden, Germany; 7 Department of Biochemistry, University of Utah, Salt Lake City, UT USA; 8 Laboratory of Nuclear Dynamics, Babraham Institute, Cambridge, UK; 9 California Institute of Technology, Pasadena, CA USA; 10 Cambridge Institute for Medical Research, Cambridge, UK; 11 Present Address: Cluster of Excellence Cellular Stress Responses in Aging-associated Diseases (CECAD), Faculty of Medicine and University Hospital Cologne, University of Cologne, Cologne, Germany

**Keywords:** Cell biology, Protein folding, Post-translational modifications, Intrinsically disordered proteins

Correction to: *Nature* 10.1038/s41586-023-06626-z Published online 18 October 2023

In the version of the article initially published, –Ψ_π_ /RT (mOsm kg^–1^) originally appeared as –Ψ_π_ (mOsm kg^–1^) in Figs. 1a, 4b and c, 5b, c and d and Extended Data Figs. 1, 4 and 10. Also, osmolality was originally labelled as osmotic potential in Fig. 5b and Extended Data Fig. 10, and osmolarity was originally labelled as osmolality in Extended Data Fig. 8. These figures, and the Supplementary Information, have now been updated to amend this error.

