# Supplementary material for: Macromolecular condensation buffers intracellular water potential
Source: Nature. 2023 Oct 18;623(7988):842–52. doi: 10.1038/s41586-023-06626-z (PMC10665201; doi:10.1038/s41586-023-06626-z)
Supplement: Supplementary file 1 — Supplementary Discussion and references specific to the Supplementary Information. [file 41586_2023_6626_MOESM1_ESM.pdf]

nature portfolio

https://doi.org/10.1038/s41586-023-06626-z

Supplementary information

Macromolecular condensation buffers intracellular water potential

In the format provided by the authors and unedited

Supplementary discussion

Chapter I/ Fitting of osmotic potential curves

As may be appreciated from Extended Data Fig.1b,d and f, the relationship between osmotic potential and concentration of various biologically important macromolecules departs markedly from linearity. The point of this supplementary discussion is to establish an empirical effective measure for quantifying departure from linearity, so that different solutes and temperatures may be compared.

PEG offers a good avenue to evaluate how departure from linearity varies as a function of physiological parameters, such as temperature, solvent composition (D_20 or H_2O) and polymer size, since PEG has no solubility limit and does not phase separate across physiological temperatures (Fig. 4a).

The osmotic potential of dilute solutions is classically modelled by van't Hoff's law (equation 1), with i the van't Hoff factor, R the gas constant, and T the temperature in Kelvins:

$$-\Psi_\pi(C,T) = iRTC \quad (1)$$

In the more general case, $-\Psi_\pi$ is fit to the polynomial equation 2, where α & β are termed the first and second virial coefficients, respectively [1–3]:

$$-\Psi_\pi(C,T) = iRTC(1 + \alpha C + \beta C^2 + \cdots) \quad (2)$$

Note that equation (2) converges to equation (1) in dilute solutions, where molecules are sparsely distributed, and that equation (2) departs markedly from linearity at high solute concentrations, where the C^2 and C^3 components become dominant. At first, we thus attempted to use equation 2 to model our osmotic potential curves in order to employ the value of the virial coefficients as an empirical measure of departure from linearity (i.e. the higher the value of the virial coefficients, the larger the departure from linearity). For this, we constrained the values of the viral coefficient to be positive.

A priori, is appears plausible that the virial coefficients might vary as a consistent function of solute identity and temperature. Note, however, that a modest temperature 10K decrease, from 310K to 300K elicits a two-fold increase in osmotic potential of BSA (Fig.1a). This is already challenging to reconcile with equation 2, which is linear with respect to temperature (in Kelvin) and instead predicts a ~3% decrease in osmotic potential with temperature decrease, rather than the observed increase.

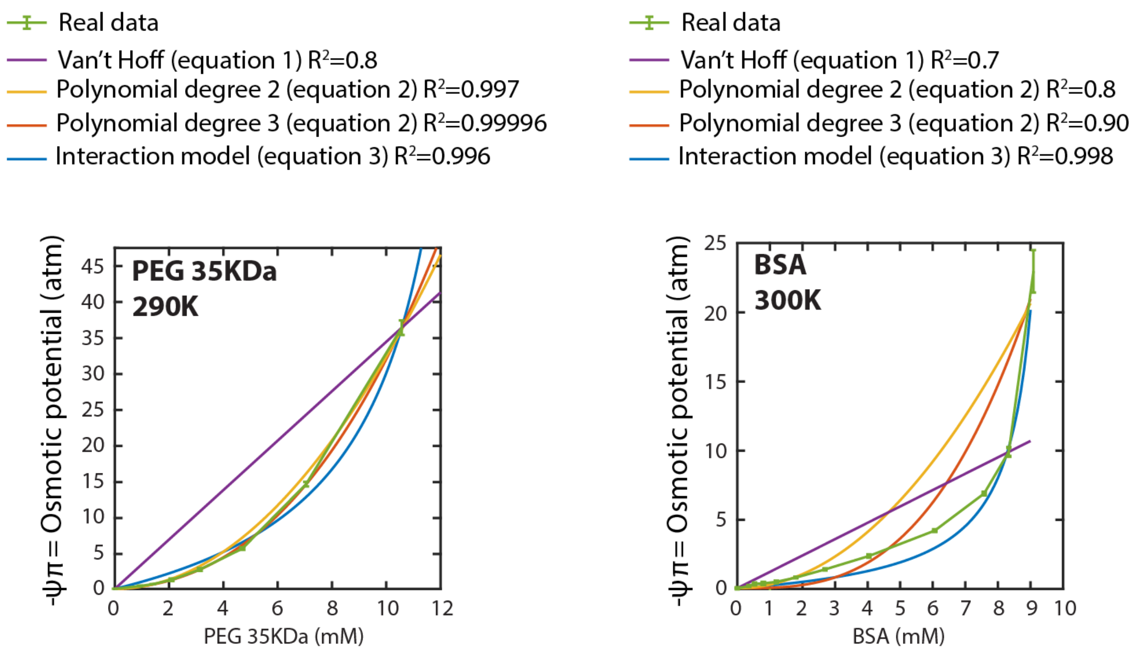

Supplementary Figure 1: Osmotic potential of indicated solutions fitted to various models.

As can be appreciated in Supplementary Figure 1, the osmotic potential of PEG 35KDa solutions as a function of concentration is poorly fit by equation (1) at 290K, and better described by equation (2). For this latter, note that the higher the order of the polynomial (i.e. the more virial coefficients allowed in the model), the better the fit.

By extensively and systematically measuring the osmotic potential of PEG solutions for different PEG sizes and different temperature (raw data in Extended Data Fig.1d), the variation in the values of the virial coefficients as a function of these parameters can be determined by simultaneously by fitting all datasets to equation 2 (see Supplementary Figure 2). We performed this analysis by considering the development of Equation 2 to the 2nd or 3rd order (that is, considering 1st or 2nd virial coefficients).

As can be observed in Supplementary Figure 2, there is no clear trend of the value of the virial coefficients as a function of PEG size nor temperature. If equation 2 was valid to model the interaction of PEG with the solvent in the ranges considered in our experiments, the virial coefficients would be expected to show consistent increase with PEG size, as larger PEG sizes display a stronger departure from linearity, which is amplified at lower temperature (Extended Data Fig.1d). Similarly, as can be observed in Supplementary Figure 1, Equation 2 fails to model the osmometry curves of BSA as a function of concentration, which exhibit a much stronger departure from linearity than PEG.

Degree 2: $-\Psi_\pi(C) = iRTC(1 + \alpha C)$

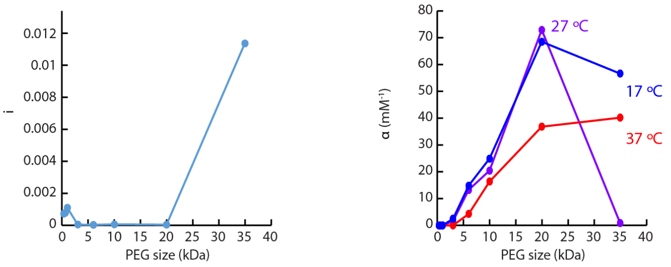

Degree 3: $-\Psi_\pi(C) = iRTC(1 + \alpha C + \beta C^2)$

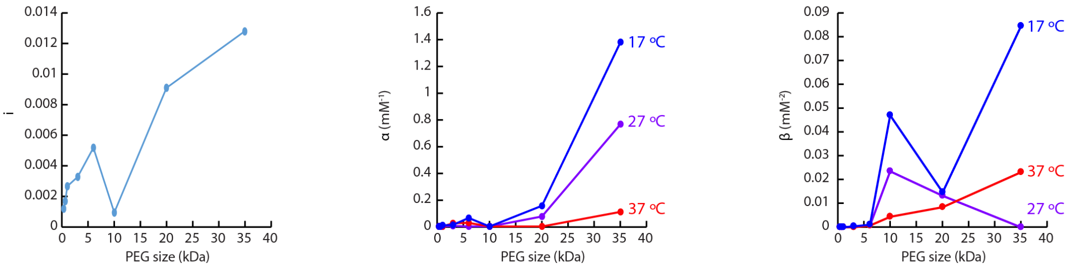

Supplementary Figure 2: Variation of the value of virial coefficient as a function of temperature and PEG size. This analysis was performed by considering a model of order two or three (that is one or two virial coefficients).

We thus considered an alternative way to model our curves, and, following the work of Fullerton and colleagues[4,5], fitted our data to equation (3), with I^s_{eff} the effective interaction term (Equation 3 corresponds to Equation 1 in the main text). Note that *a priori*, I^s_{eff} and A may be functions that vary with solute identity and temperature.

$$-\Psi_\pi(C,T) = \frac{A(T) \times C}{1 - I^s_{eff}(T) \times C} \quad (3)$$

As can be observed in Supplementary Figure 1, Equation (3) fits well the osmometry curves of PEG 35kDa, and is markedly better at fitting the osmotic potential of BSA, which exhibits a much stronger departure from linearity than PEG.

Since PEG measurements were performed at multiple temperatures, we thought to constrain the fits to avoid fitting two parameters ($A(T)$ and $I^s_{eff}(T)$) with only one curve. Indeed, an interesting limit case of equation (3) is when $I^s_{eff}(T) \times C \ll 1$. In these conditions, A tends to iRT (that is the van't Hoff equation, equation 1), thus, we hypothesized that A should be linear with temperature, and thus fitted simultaneously all curves from different temperatures to equation 4.

$$-\Psi_\pi(C,T) \approx \frac{A'T \times C}{1 - I^s_{eff}(T) \times C} \quad (4)$$

In this case, A' is a constant that is identical for all the datasets from a given macromolecule, meaning that for three datasets at three different temperatures, only four parameters are considered, rather than six. This provided similar results to fitting each curve to equation 3, albeit with better goodness of fits (for our PEG data set of 8 molecular weights and 3 temperatures, so 24 datapoints, Spearman correlation of 0.9948 and p<0.0001 between the values of I^{s}_{eff} obtained by fitting each curve independently or all curves simultaneously).

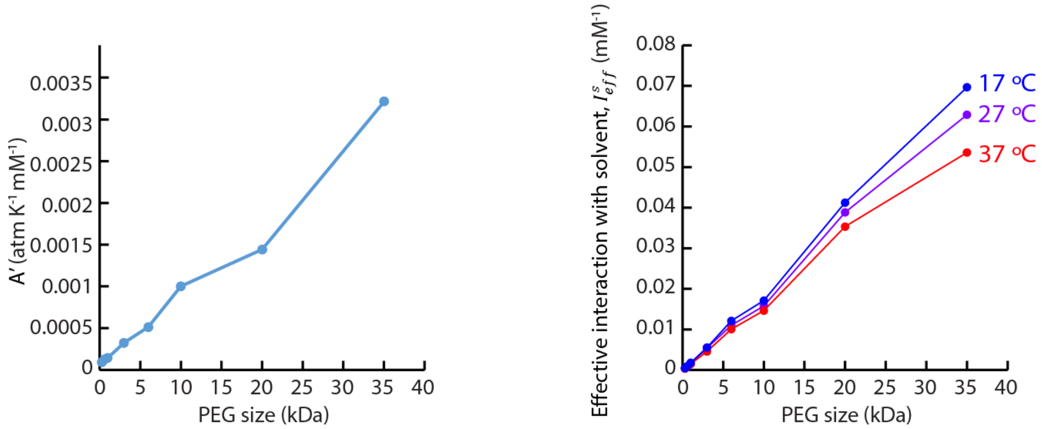

Supplementary Figure 3: Variation of the value of I^{s}_{eff} as a function of temperature and PEG size. This analysis was performed by considering equation 4.

As can be observed in Supplementary Figure 3, fitting our dataset to Equation (4) provided much more consistent results as a function of PEG size and temperature compared with Equation (2). We found that I^{s}_{eff} scales quasi-linearly with PEG size, and the slope of this curve is higher at lower temperature. Note also that A' scales quasi-linearly with PEG size, which is expected: A' is related to i, the van't Hoff factor, which is expected to increase with PEG size. Thus, throughout this paper, we systematically fit our data using equation (4) and used I^{s}_{eff} as an empirical measure of the impact of the solute upon the solvent.

Note that equation 4 can be rearranged as equation 5. This highlights the two contributions to the osmotic potential: $A'TC$ models the effect of an ideal solute on the solvent, whereas $\frac{1}{1-I^{s}_{eff}(T)C}$ models the extent of the unfavourable interactions at the solute:solvent interface.

$$-\Psi_{\pi}(C,T) \approx A'TC \times \frac{1}{1 - I^{s}_{eff}(T)C} \quad (5)$$

Chapter II/ Other frameworks for modelling polymer phase behaviour and their application to biomolecular condensation in cells.

Most theoretical frameworks for phase separation consider entropic and enthalpic effects from the standpoint of the polymer. For example, Flory-Huggins solution theory (FHT) was developed to describe the thermodynamics of polymer-solvent mixtures, expressing the free energy of mixing in terms of solvent and polymer volume fractions, temperature and a single interaction parameter χ to describe the strength of solvent-polymer interactions compared with average solvent-solvent and polymer-polymer interactions[6]. This lattice model considers the entropic and enthalpic drivers of polymer behaviour, with the value of χ predicting whether the mixture is homogeneous or forms two coexisting phases, and was subsequently extended to mixtures of two or more polymers by Scott and Tompa[7,8]. FHT has been successfully used to model the phase behaviour of, and fit data from, macromolecular solutions *in vitro,* where condensation is examined as a function of the concentration of the specific protein(s) under investigation (e.g. [9]), but makes several critical assumptions that limit its direct applicability to proteins and their interactions within cells:

- FHT assumes the polymer is made from identical (non-polar) monomers. This is not true for any proteins in cells, as the cytosol is composed from a mixture of thousands of different proteins, whose relative abundance varies by several orders of magnitude.
- Under the FHT mean field approximation, all polymer segments interact equally. This is not true for any natural polypeptides which are composed from amino acid residues with differing chemical properties (charged vs uncharged, hydrophilic vs hydrophobic, bulky vs small, aromatic vs aliphatic etc), and whose activity is further modulated by site-specific post-translational modifications such as phosphorylation.
- FHT assumes ideal polymer chain behaviour, and does not take into account chain connectivity - interactions between different polymer regions or any resulting topological constraints. Such interactions occur in almost all cellular proteins, leading to secondary and tertiary structures that are quite specific to the primary amino acid sequence, with most soluble proteins in the cytoplasm adopting compact globular structures.
- χ is used to describe all interactions in the system, and so cannot adequately account for specific interactions critical to the behaviour of specific proteins in biological systems (e.g. electrostatic interactions, hydrophobic interactions and hydrogen bonding). Indeed, the physics of

hydrophobic surface hydration itself are not sufficiently well understood that the free energy change can be predicted reliably [10].

- FHT considers a homogenous solvent, whereas the cytosol is a highly complex, heterogeneous and crowded environment, in which the activity of hydration water has been experimentally distinguished from bulk solvent by multiple independent methods. These heterogeneities and spatial constraints are not taken into consideration by FHT [10–12].
- FHT assumes that polymers and solvent molecules can move freely and do not impose constraints on each other's mobility. This is not the case for water molecules hydrating proteins and other macromolecules, which have reduced translational and rotational entropy compared with bulk solvent. Moreover, the free energy of solvent-sidechain interactions differs enormously between hydrophobic and polar/charged amino acid residues, and also varies with context [10].

Overall then, while FHT can accurately describe the phase behaviour of dilute binary polymer blends, it does not describe the unique behaviours of water as a solvent, nor does it capture the behaviour of complex macromolecular mixtures found in biological systems without substantial modifications and extensions that try to account for specific interactions and factors such as chain connectivity and molecular crowding. The review by Zaslavsky and Uversky[13] provides a more detailed perspective on these themes, highlighting that reversible condensation of natively folded proteins does not occur in any solvent besides water and its importance to this process.

More recent attempts to model the thermodynamics of protein condensation in cells have explicitly considered changes in free energy of the solvent in a more granular fashion than FHT [14], but in general, models of cellular phase separation focus on the enthalpic driving force generated by weak, multivalent interactions between macromolecules. Solvent entropy rarely receives the same level of consideration even though water is the most abundant molecular species in any biological system and thus a major factor determining its thermodynamical equilibria. To our knowledge, the multitude of different theoretical frameworks that aim to describe liquid-liquid phase separation of proteins do not predict the non-linear relationship between BSA concentration and osmotic potential, for example, nor the interaction with temperature we observe across the physiological range. By focussing on the solvent and employing the Fullerton empirical model that explicitly considers the interaction between polymers and water[4,5], as described above, our work provides a framework for understanding the effects of temperature on the behaviour of concentrated polymer solutions and the impact of manipulations of solvent thermodynamics, e.g., heavy water. Given the concentrated, colloidal intracellular environment, we hope that this approach will aid our understanding of the physiological drivers and functions of phase separation in cells.

Supplementary References

1. Scatchard, G. Physical Chemistry of Protein Solutions. I. Derivation of the Equations for the Osmotic Pressure. *Journal of the American Chemical Society* **68**, 2315–2319 (1946).

2. Vink, H. Precision measurements of osmotic pressure in concentrated polymer solutions. *European Polymer Journal* **7**, 1411–1419 (1971).

3. Mitchison, T. J. Colloid osmotic parameterization and measurement of subcellular crowding. *Molecular Biology of the Cell* **30**, 173–180 (2019).

4. Zimmerman, R. J., Kanal, K. M., Sanders, J., Cameron, I. L. & Fullerton, G. D. Osmotic pressure method to measure salt induced folding/unfolding of bovine serum albumin. *J Biochem Biophys Methods* **30**, 113–131 (1995).

5. Cameron, I. L., Kanal, K. M., Keener, C. R. & Fullerton, G. D. A mechanistic view of the non-ideal osmotic and motional behavior of intracellular water. *Cell Biol Int* **21**, 99–113 (1997).

6. Flory, P. J. Thermodynamics of High Polymer Solutions. *J Chem Phys* **10**, 51–61 (1942).

7. Tompa, H. Phase relationships in polymer solutions. *Transactions of the Faraday Society* **45**, 1142–1152 (1949).

8. Scott, R. L. The Thermodynamics of High Polymer Solutions. V. Phase Equilibria in the Ternary System: Polymer 1—Polymer 2—Solvent. *J Chem Phys* **17**, 279–284 (1949).

9. Nott, T. J. *et al.* Phase Transition of a Disordered Nuage Protein Generates Environmentally Responsive Membraneless Organelles. *Mol Cell* **57**, 936–947 (2015).

10. Rego, N. B. & Patel, A. J. Understanding Hydrophobic Effects: Insights from Water Density Fluctuations. *https://doi.org/10.1146/annurev-conmatphys-040220-045516* **13**, 303–324 (2022).

11. Persson, E. & Halle, B. Cell water dynamics on multiple time scales. *Proc Natl Acad Sci U S A* **105**, 6266–6271 (2008).

12. Ebbinghaus, S. *et al.* An extended dynamical hydration shell around proteins. *Proc Natl Acad Sci U S A* **104**, 20749–20752 (2007).

13. Zaslavsky, B. Y. & Uversky, V. N. In Aqua Veritas: The Indispensable yet Mostly Ignored Role of Water in Phase Separation and Membrane-less Organelles. *Biochemistry* **57**, 2437–2451 (2018).

14. Bartolucci, G., Michaels, T. C. T. & Weber, C. A. The interplay between molecular assembly and phase separation. *bioRxiv* 2023.04.18.537072 (2023) doi:10.1101/2023.04.18.537072.
