# Supplementary material for: Macromolecular condensation buffers intracellular water potential
Source: Nature. 2023 Oct 18;623(7988):842–52. doi: 10.1038/s41586-023-06626-z (PMC10665201; doi:10.1038/s41586-023-06626-z)
Supplement: Supplementary file 2 — Reporting Summary [file 41586_2023_6626_MOESM2_ESM.pdf]

nature portfolio

Corresponding author(s): Rachel Edgar
John O'Neill
Emmanuel Derivery

Last updated by author(s): 16/08/2023

Reporting Summary

Nature Portfolio wishes to improve the reproducibility of the work that we publish. This form provides structure for consistency and transparency in reporting. For further information on Nature Portfolio policies, see our Editorial Policies and the Editorial Policy Checklist.

Statistics

For all statistical analyses, confirm that the following items are present in the figure legend, table legend, main text, or Methods section.

n/a	Confirmed	
☐	☒	The exact sample size (*n*) for each experimental group/condition, given as a discrete number and unit of measurement
☐	☒	A statement on whether measurements were taken from distinct samples or whether the same sample was measured repeatedly
☐	☒	The statistical test(s) used AND whether they are one- or two-sided<br>*Only common tests should be described solely by name; describe more complex techniques in the Methods section.*
☐	☒	A description of all covariates tested
☐	☒	A description of any assumptions or corrections, such as tests of normality and adjustment for multiple comparisons
☐	☒	A full description of the statistical parameters including central tendency (e.g. means) or other basic estimates (e.g. regression coefficient) AND variation (e.g. standard deviation) or associated estimates of uncertainty (e.g. confidence intervals)
☐	☒	For null hypothesis testing, the test statistic (e.g. *F*, *t*, *r*) with confidence intervals, effect sizes, degrees of freedom and *P* value noted<br>*Give P values as exact values whenever suitable.*
☒	☐	For Bayesian analysis, information on the choice of priors and Markov chain Monte Carlo settings
☒	☐	For hierarchical and complex designs, identification of the appropriate level for tests and full reporting of outcomes
☒	☐	Estimates of effect sizes (e.g. Cohen's *d*, Pearson's *r*), indicating how they were calculated

Our web collection on statistics for biologists contains articles on many of the points above.

Software and code

Policy information about availability of computer code

Data collection	Microscopy data was collected using Metamorph software v7.10.1.161. or Zeiss software Aim version 4.2
Data analysis	Unless otherwise specified, image analysis was performed using Fiji (ImageJ version: 1.53f) and Matlab 2020b (Mathworks) as detailed in the methods. Our general image registration and wavelet filtering codes can also be found on our Github page (https://github.com/deriverylab/GPU_registration and https://github.com/deriverylab/GPU_wavelet _a_trous). All lookup tables applied to images in this paper come from the collection from James Manton (https://github.com/jdmanton/ImageJ_LUTs).<br>Statistical analyses were performed using GraphPad Prism v9.4.0 (673).<br>Figures were assembled in Adobe Illustrator 2021. Movies were edited in Adobe Premiere 2021.<br>Raw proteomics files from LC-MS/MS were processed using MaxQuant (v.1.6.6.0) with the integrated Andromeda search engine. MaxQuant output file was then processed with Perseus (v1.6.6.0). Further data processing was performed in R v3.6.1 with R Studio v1.2

For manuscripts utilizing custom algorithms or software that are central to the research but not yet described in published literature, software must be made available to editors and reviewers. We strongly encourage code deposition in a community repository (e.g. GitHub). See the Nature Portfolio guidelines for submitting code & software for further information.

Data

Policy information about availability of data

All manuscripts must include a data availability statement. This statement should provide the following information, where applicable:

- Accession codes, unique identifiers, or web links for publicly available datasets
- A description of any restrictions on data availability
- For clinical datasets or third party data, please ensure that the statement adheres to our policy

The mass spectrometry proteomics will be deposited to the ProteomeXchange Consortium via the PRIDE partner repository (#PXD044481 processed proteomics results are provided in supplementary tables 1 and 2). Numerical data to generate all plots in this manuscript are provided in Source data.
Custom image processing code specific to this paper can be found on our GitHub page (https://github.com/deriverylab/granulosityindex and https://github.com/deriverylab/nuclearsegmentation). All other data supporting the findings of this study are available from the corresponding authors on reasonable request.

Human research participants

Policy information about studies involving human research participants and Sex and Gender in Research.

Reporting on sex and gender	N/A
Population characteristics	N/A
Recruitment	N/A
Ethics oversight	N/A

Note that full information on the approval of the study protocol must also be provided in the manuscript.

Field-specific reporting

Please select the one below that is the best fit for your research. If you are not sure, read the appropriate sections before making your selection.

☒ Life sciences ☐ Behavioural & social sciences ☐ Ecological, evolutionary & environmental sciences

For a reference copy of the document with all sections, see nature.com/documents/nr-reporting-summary-flat.pdf

Life sciences study design

All studies must disclose on these points even when the disclosure is negative.

Sample size	Numbers of biological replicates were chosen based on preliminary experiments, so that an effect size of at least 10% could be detected between experimental groups for α=0.05 and β=0.9.
Data exclusions	No data was excluded.
Replication	All representative results shown were performed multiple times independently with similar results. Details on the replication of each experiment are provided in the respective figure legend and/or in the "Statistics and Reproducibility" dedicated chapter in the methods
Randomization	Samples and cultures were randomly allocated to each group, and subsequently handled and processed identically. No potential covariates could be identified that required any additional controls.
Blinding	Investigators were not blinded during data collection as all replicates (controls and treatments) were collected/extracted and/or analyzed using the same procedure within each experiment (objective measurements). Where practical, investigators were ignorant to the hypothesis.

Reporting for specific materials, systems and methods

We require information from authors about some types of materials, experimental systems and methods used in many studies. Here, indicate whether each material, system or method listed is relevant to your study. If you are not sure if a list item applies to your research, read the appropriate section before selecting a response.

Materials & experimental systems

n/a	Involved in the study
☐	☒ Antibodies
☐	☒ Eukaryotic cell lines
☒	☐ Palaeontology and archaeology
☐	☒ Animals and other organisms
☒	☐ Clinical data
☒	☐ Dual use research of concern

Methods

n/a	Involved in the study
☒	☐ ChIP-seq
☒	☐ Flow cytometry
☒	☐ MRI-based neuroimaging

Antibodies

Antibodies used

FusLC-GFP immunoprecipitation was performed using GFP-Trap Resin (#gta-20, Chromotek). GFP western blot was performed using primary anti-GFP antibodies (ab290, abcam,, 1:10 000 dilution) and secondary anti-rabbit HRP-conjugated secondary antibodies (A6154-1ML, Sigma, 1:10 000 dilution)

Validation

antibodies were validated by the respective manufacturers:
https://www.ptglab.com/products/GFP-Trap-Agarose-gta.htm
https://www.abcam.com/products/primary-antibodies/gfp-antibody-ab290.html
chrome-extension://efaidnbmnnnibpcajpcglclefindmkaj/https://www.sigmaaldrich.com/specification-sheets/695/205/A6154-BULK.pdf

Eukaryotic cell lines

Policy information about cell lines and Sex and Gender in Research

Cell line source(s)

Expi293F (Thermofisher) ; SH-SY5Y (ATCC #CRL-2266) ; U2OS (ATCC #HTB-96) ; human foreskin fibroblasts (ATCC) and Raji cells (gift from P. Farrell, described in Laux et al. 1988) ; Sf9 (Thermofisher)

Authentication

Cell line used was not authenticated as provided by a commercial supplier

Mycoplasma contamination

Dapi staining did not reveal the presence of Mycoplasms

Commonly misidentified lines
(See ICLAC register)

No commonly misidentified lines were used

Animals and other research organisms

Policy information about studies involving animals; ARRIVE guidelines recommended for reporting animal research, and Sex and Gender in Research

Laboratory animals

mouse

Wild animals

not relevant

Reporting on sex

not relevant

Field-collected samples

not relevant

Ethics oversight

For primary chondrocyte extraction, animal experiments were approved by the animal welfare committee at the University of Manchester. For primary mouse lung fibroblasts - mouse work was overseen by the Animal Welfare and Ethical Review Body of the MRC Laboratory of Molecular Biology. A statement is included to this effect in the methods section.

Note that full information on the approval of the study protocol must also be provided in the manuscript.
