# Supplementary figures and images for: Macromolecular condensation buffers intracellular water potential
Source: Nature. 2023 Oct 18;623(7988):842–52. doi: 10.1038/s41586-023-06626-z (PMC10665201; doi:10.1038/s41586-023-06626-z)

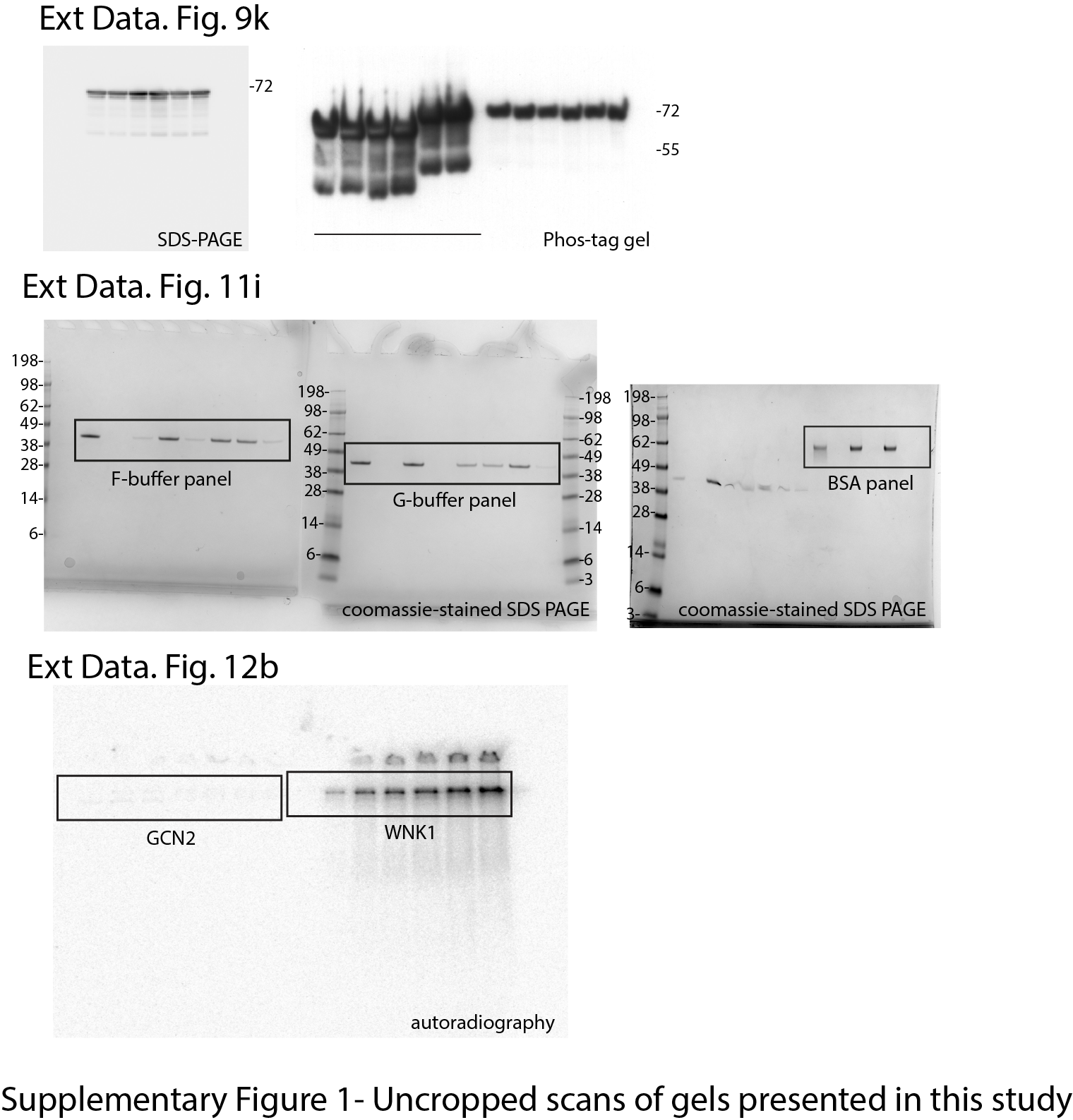

Supplement: Supplementary file 3 — Full gels for all western blot, Coomassie-stained and autoradiography gels presented in this study. [file 41586_2023_6626_MOESM3_ESM.tif]
